# An AI-based gravitrap surveillance for spatial interaction analysis in predicting aedes risk

**DOI:** 10.1186/s12942-025-00403-z

**Published:** 2025-08-06

**Authors:** Hsiang-Yu Yuan, Pei-Sheng Lin, Wei-Liang Liu, Tzai-Hung Wen, Yu-Chun Lu, Chun-Hong Chen, Li‑Wei Chen

**Affiliations:** 1 Department of Biomedical Sciences, City University of Hong Kong, College of Biomedicine, Hong Kong SAR, China; 2 Centre for Applied One Health Research and Policy Advice Jockey Club College of Veterinary Medicine and Life Sciences, City University of Hong Kong, Hong Kong SAR, China; 3 Institute of Population Health Sciences, National Health Research Institutes, Miaoli, Taiwan; 4 National Mosquito-Borne Diseases Control Research Center, National Health Research Institutes, Miaoli, Taiwan; 5 Department of Geography, National Taiwan University, Taipei, Taiwan; 6 National Institute of Infectious Diseases and Vaccinology, National Health Research Institutes, Miaoli, Taiwan; 7 Department Institute of Molecular and Cellular Biology, National Taiwan University, Taipei, Taiwan

**Keywords:** *Aedes* index, AI method, Auto-Markov model, Dengue prevention, Gravitrap, Spatial–temporal patterns

## Abstract

**Background:**

Dengue fever is transmitted to humans through bites of Aedes mosquito vectors. Therefore, controlling the Aedes population can decrease the incidence and block transmission of dengue fever and other diseases transmitted by these mosquito species. In many countries, gravitraps are used to monitor mosquito vector densities, but this approach usually underestimates the population of Aedes mosquitoes. Moreover, literature on the spatio-temporal dynamics of Aedes populations in a single city is limited. For example, in Kaohsiung of Taiwan, population densities vary substantially between villages, and the city government has relatively limited resources to deploy gravitraps. Therefore, a well-defined index should be developed to reflect the spatial–temporal dynamics of adult Aedes mosquitoes in urban environments. This would allow reduction of sources and removal of vector habitats under various situations.

**Methods:**

An artificial intelligence (AI) surveillance based on an auto-Markov model with a non-parametric permutation test is proposed. The auto-Markov model takes neighborhood effects into consideration, and can therefore adjust spatial–temporal risks dynamically in various seasons and environmental background. Information from neighboring villages is incorporated into the model to enhance precision of risk prediction.

**Results:**

The proposed AI gravitrap index integrates the auto-Markov and disease mapping models to enhance sensitivity in risk prediction for Aedes densities. Simulation studies and cross-validation analysis indicated that the AI index could be more efficient than traditional indices in assessing risk levels. This means that using the AI index could also reduce allocation cost for gravitraps. Moreover, since the auto-Markov model accommodates spatial–temporal dependence, a risk map by the AI index could reflect spatial–temporal dynamics for Aedes densities more accurate.

**Conclusions:**

The AI gravitrap index can dynamically update risk levels by the auto-Markov model with an unsupervised permutation test. The proposed index thus has flexibility to apply in various cities with different environmental background and weather conditions. Furthermore, a risk map by the AI index could provide guidance for policymakers to prevent dengue epidemics.

**Supplementary Information:**

The online version contains supplementary material available at 10.1186/s12942-025-00403-z.

## Background

Dengue fever is a rapidly-spreading mosquito-borne disease with a worldwide incidence rate that has increased 30-fold over the last 50 years [1]. Dengue virus is transmitted to humans through bites of carrier Aedes mosquito vectors, and some studies have reported a positive correlation between mosquito densities and dengue incidence [2–7]. Since a cure for dengue fever is yet to be found, vector control is important for its prevention. The control of Aedes population can also reduce the incidence rates of other diseases transmitted by these mosquito species, such as Zika, chikungunya, and yellow fever [8]. Environmental management, such as cleaning of mosquito breeding sites, is critical for vector control. Inaccurate evaluation of vector indices could lead to ineffective vector control in areas with higher risk of dengue outbreaks during environmental management efforts. In this study, we developed an artificial intelligence (AI) algorithm for gravitrap index to dynamically map the population densities of Aedes mosquitoes. This method enhances the efficacy of vector index evaluation, which traditionally excludes neighborhood effects, leading to better monitoring of the spatial–temporal dynamics of Aedes population distribution as determined using surveillance systems.

Although various methods and techniques have been developed to monitor the densities of vector mosquitoes [9–13], there are limitations to their effectiveness. For example, some host-seeking traps are simple but require electricity, limiting their deployment. Oviposition traps may underestimate Aedes populations because a single female mosquito may lay eggs in several containers. The applicability of widely used mosquito indices, such as the House Index and Breteau Index, to dengue transmission has been subject to debate [8]. For preventing dengue transmission and developing control strategies, data on adult Aedes population distribution are more important than egg- and larva-associated indices [2].

Recently, gravitraps have been used to monitor the densities of vector mosquitoes in many localities, such as Hong Kong and Singapore, to replace old systems [14, 15]. Kaohsiung, a densely populated Taiwanese city with a large seaport [16], has also adopted this method. A gravitrap is a simple black cylinder with water at the bottom that mimics mosquito breeding sites, attracting females for oviposition. The inner wall of the gravitrap is covered with adhesive to capture ovipositing female mosquitoes [17]. Unlike ovitraps, gravitraps do not become larval habitats and serve as a population control measure for Aedes mosquitoes, in addition to being tools for monitoring their numbers. The data from gravitraps help to identify areas with high population densities.

However, gravitrap indices for estimating Aedes mosquito populations have some limitations [8, 18]. First, the number of mosquitoes captured by gravitraps greatly varies depending on location and seasons. Second, gravitraps may underestimate mosquito populations. For instance, when certain locations have moderately higher densities, their traps may all indicate zero captures due to limitations of detection capability. This situation would lead to an underestimation of mosquito density in those areas. This can be particularly true in Taiwan and Hong Kong, which exhibit both tropical and temperate characteristics. Additionally, the lack of literature on the spatial–temporal dynamics of Aedes populations within individual cities adds to the difficulty of developing more effective citywide vector control strategies [8].

Because of the drawback mentioned above, a local scale of spatial–temporal units for gravitrap indices should be developed. Recent studies have shown that machine learning (ML) offers promising tools for mapping Aedes mosquito distribution with greater accuracy. High-resolution spatial simulations using ML models such as Random Forest and Gradient Boosting Machines have demonstrated strong predictive performance in global mapping efforts of Aedes aegypti and Aedes albopictus, providing critical insights for public health planning [19]. Other models, including Support Vector Machines and MaxEnt, have also been effective in predicting mosquito habitats by integrating environmental and spatial data, often outperforming traditional methods [20, 21]. Although some ML methods, such as random forest and gradient boosting, are commonly used for risk prediction in epidemiology, these approaches generally lack consideration of spatial–temporal dependence structures inherent in responses [22]. In the meantime, an auto-Markov model [23] is widely utilized to construct hierarchical structures for spatial–temporal observations. This modelling strategy can provides a network to compensate prediction accuracy for those regions without enough data information.

In our gravitrap study, the Kaohsiung city administration typically deploys gravitraps in select hot-spot areas due to limited resources and significant variations in Aedes population densities between villages. So, villages close to those with consistently high Aedes species populations must be taken into account to identify local distribution patterns of these mosquitoes. For spatial and temporal data analysis, the Markov random field model that takes neighborhood effects into account represents a useful tool. By combining auto-Markov and disease mapping models, we developed an AI gravitrap index with improved sensitivity to local changes. The study aimed to enhance the efficacies of traditional location- and time-independent gravitrap indices for risk levels and update them to depict the spatial–temporal dynamics of Aedes population densities.

## Methods

### Sample data

The environmental protection bureau of the Kaohsiung city government has been deploying gravitraps for Aedes mosquito surveillance since 2016. In this study, we used gravitrap data for 2017, when a total of 19,643 gravitraps were placed by the environment protection bureau in nine administrative districts comprising 440 villages with frequent dengue outbreaks. The gravitraps were placed in shaded areas or around potted plants which mosquitoes tend to rest on. Normally, each gravitrap was placed at a site for about 2 weeks. A total of 6,768 female Aedes mosquitoes were captured. Although both *Ae. aegypti* and *Ae. albopictus* were found in the gravitraps, the former was more abundant.

### Aedes density measurement

We first defined an area density measure similar to the gravitrap indices used in Singapore and Hong Kong (HK) [2, 24]. In this index, $${{\varvec{s}}}_{i}$$ denotes the $$i$$-th village ($$i=1,...,n$$*,* where $$n$$ = total number of villages in the study area), $$T$$ denotes the total number of sampling weeks, and $${m}_{i,t}$$ and $${g}_{i,t}$$ are the numbers of captured female Aedes mosquitoes and deployed gravitraps, respectively, in the $$i$$-th village in week $$t$$ ($$i=1,...,n$$; $$t=1,...,T)$$. The Aedes density measure (ADM) in village $${{\varvec{s}}}_{i}$$ in week $$t$$ is given as $${D}_{i,t}={m}_{i,t}/{g}_{i,t}$$. The ADM is calculated by dividing the number of captured Aedes mosquitoes by the number of deployed gravitraps.

### Significant variables for high ADMs

${H}_{0}$ Denotes a null hypothesis that each village ${{\varvec{s}}}_{i}$ in time $t$ has the same density of Aedes mosquitoes. To evaluate whether some villages had a high ADM ${D}_{i,t}$ (i.e., rejection of ${H}_{0}$) in a given time period, we conducted a permutation test. $M=\sum_{t=1}^{T}{\sum }_{i=1}^{n}{m}_{i,t}$ and $G=\sum_{t=1}^{T}{\sum }_{i=1}^{n}{g}_{i,t}$ represented the total numbers of captured Aedes mosquitoes and deployed gravitraps, respectively, in the study area across all time periods. We performed 1,000 simulations for the permutation test. The following algorithm shows the calculation of the critical value for the one-sided test with a significance threshold of $\alpha =0.05$.

**Algorithm 1:**
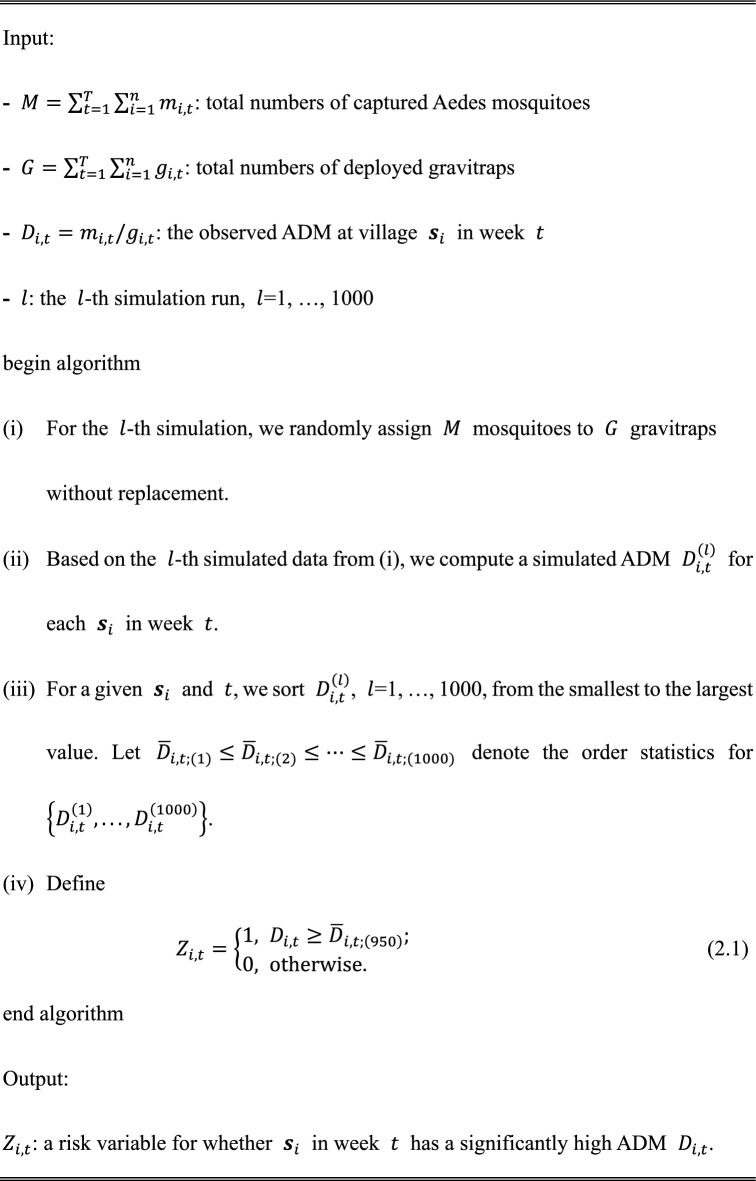
Permutation test:

${\overline{D} }_{i,t;(950)}$ in (2.1) is a simulated critical value with a significance of $\alpha =0.05$ in the permutation test. When ${Z}_{i,t}=1$, village ${{\varvec{s}}}_{i}$ in week $t$ has a high density of Aedes mosquitoes. Therefore, ${Z}_{i,t}$ is a significant variable. The algorithm for the permutation test to compute ${Z}_{i,t}$ is provided in Algorithm 1.

### Weighted risks with neighborhood effects

#### Auto-Markov models

**Fig. 1:**
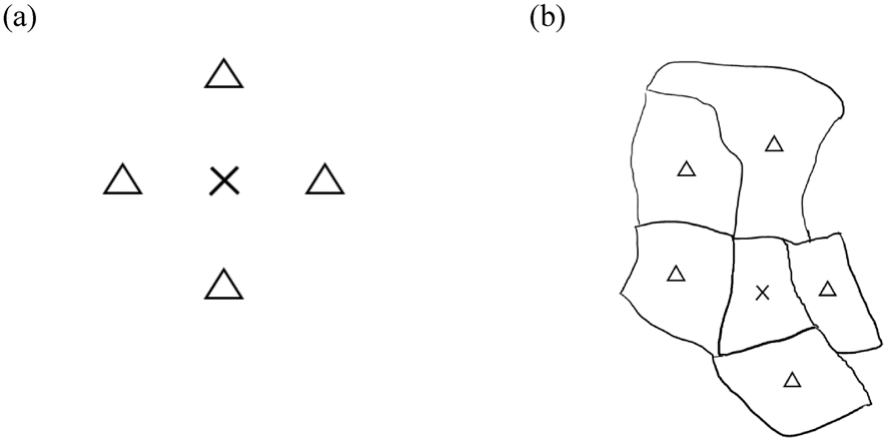
First-order neighborhood structures in the Markov random field for **a** gridded data and **b** irregular data. Notations: ×, a given village; ∆, first-order neighbors

We developed an auto-logistic model to estimate the weighted risk of high Aedes density in ${{\varvec{s}}}_{i}$ in week $t$. An auto-Markov random field is frequently used to model observations with spatial association between responses. With a suitable neighborhood structure, the Markov random field can incorporate spatial dependence into models [23]. Figure 1 depicts a first-order neighborhood structure in the Markov random field for gridded and irregular data. Specifically, ${{\varvec{s}}}_{i}$ represents a given village. For gridded data, first-order neighbors of ${{\varvec{s}}}_{i}$ are “one-step” villages to ${{\varvec{s}}}_{i}$, and in irregular data, first-order neighbors are a collection of villages that share a common border with ${{\varvec{s}}}_{i}$.

Let *B(1) i* represent a collection of first-order neighbors for${{\varvec{s}}}_{i}$, and let ${Z}_{i,t}$ denote a risk variable for significantly high ADM${D}_{i,t}$. Conditional on neighboring risk variables, we use $\theta$_*i,t*_ = *P* (*Z*_*i,t*_ = 1 | *Z*_*j,t*_: ${{\varvec{s}}}_{j}$ $\in$ *B(1) i*) to denote the risk probability that village ${{\varvec{s}}}_{i}$ in week $t$ has a significantly high ADM${D}_{i,t}$. $\eta$_*i,t*_ = exp (*β*_0_ + *β*_1_ Σ_*j*∈*B(1) i*_*Z*_*j,t*_). A first-order auto-logistic model for ${\theta }_{i,t}$ is given by:

2.2 $$\theta_{i,t} = \,{{\eta_{i,t} } \mathord{\left/ {\vphantom {{\eta_{i,t} } {\left( {1 + \eta_{i,t} } \right)}}} \right. \kern-0pt} {\left( {1 + \eta_{i,t} } \right)}}$$

In (2.2), ${\beta }_{0}$ and ${\beta }_{1}$ are estimated using a pseudo-likelihood method [20]. The algorithm for the estimation method is given below:

**Algorithm 2:**
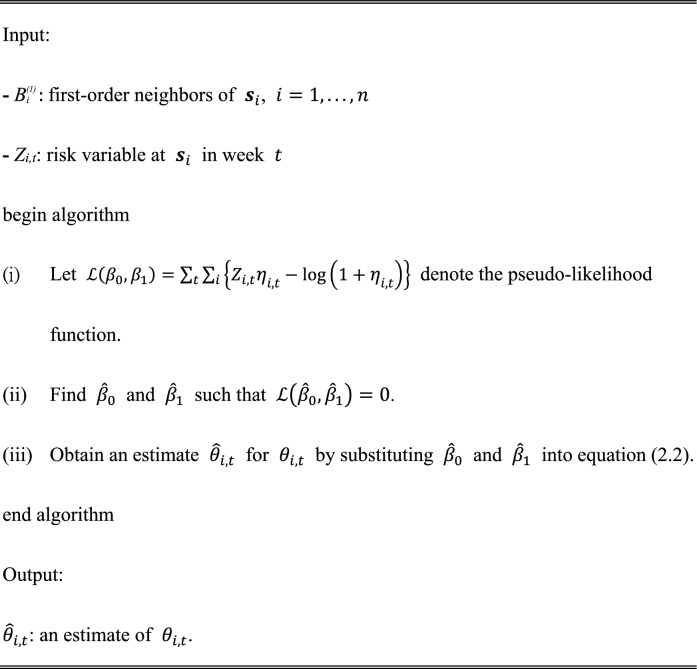
Estimation method for auto-logistic models

#### Rational for choice of the first-order neighborhood structure

In the Kaohsiung geographic structure used in this paper, we have 96,580 pairs of villages with a mean value of distances between villages about 2.7 km. (The distance between villages was computed based on distance between their administration centers.) Since the flying distance of mosquitoes is about 200 m, it is reasonable to use the first-order neighborhood structure to construct an auto-logistic model for weighted risks. Furthermore, confusion matrices for comparisons of classified results by the first- and second-order neighborhood structures were computed (see Table A1 of Supplementary Material).

#### Significance of weighted risks

${\theta }_{i,t}$ Can be considered a weighted risk for high density of Aedes mosquitoes by incorporating neighborhood information. For convenience, ${\theta }_{i,t}$ is referred to as the weighted risk for high density. ${\widehat{\theta }}_{i,t}$ denotes the estimate for ${\theta }_{i,t}$ from Algorithm 2. To decide whether ${\widehat{\theta }}_{i,t}$ is significantly high, a permutation test (with 1000 simulation runs) similar to Algorithm 1 is used to compute a simulated 100(1-$\tau$)% confidence interval, where $\tau$ denotes the level of significance for weighted probabilities. ${\overline{\theta }}_{i,t;100(1-\tau )}$ denotes the critical value simulated by the permutation test. The value of ${\overline{\theta }}_{i,t;100(1-\tau )}$ is used to evaluate the significance of ${\widehat{\theta }}_{i,t}$ as a criterion for determining the risk groups of Aedes densities.

### An AI gravitrap index

Our gravitrap index categorizes the observed ADM ${D}_{i,t}$ into three risk levels: high, moderate, and low (Table 1). If the significant variable ${Z}_{i,t}$ is equal to one, the ADM in village ${{\varvec{s}}}_{i}$ in week $t$ is significantly high, and therefore, categorized as high-risk. When the significant variable ${Z}_{i,t}$ is equal to 0, but taking neighborhood effects into account, the weighted risk ${\widehat{\theta }}_{i,t}$ for high density is beyond the simulated threshold (i.e., ${\widehat{\theta }}_{i,t}\ge {\overline{\theta }}_{i,t;100(1-\tau )}$), village ${{\varvec{s}}}_{i}$ in week $t$ is categorized as moderate-risk. If both ${Z}_{i,t}$ and ${\widehat{\theta }}_{i,t}$ are lower than the thresholds but ${D}_{i,t}>0,$ which means that gravitraps in village ${{\varvec{s}}}_{i}$ in week $t$ still catch Aedes mosquitoes, the assigned category is low-risk.

**Table 1:** Criteria for risk levels by combination of ADM ${D}_{i,t}$, significant variable ${Z}_{i,t}$ and weighted risk ${\widehat{\theta }}_{i,t}$, where ${\overline{\theta }}_{i,t;0.95}$ denotes a threshold for the weighted probability

Category	Risk level	Color
${{\varvec{Z}}}_{{\varvec{i}},{\varvec{t}}}=1$	High	Red
${{\varvec{Z}}}_{{\varvec{i}},{\varvec{t}}}=0$*,* ${\widehat{{\varvec{\theta}}}}_{{\varvec{i}},{\varvec{t}}}\ge {\overline{{\varvec{\theta}}} }_{{\varvec{i}},{\varvec{t}};0.95}$	Moderate	Yellow
${{\varvec{Z}}}_{{\varvec{i}},{\varvec{t}}}=0$*, *${\widehat{{\varvec{\theta}}}}_{{\varvec{i}},{\varvec{t}}}<{\overline{{\varvec{\theta}}} }_{{\varvec{i}},{\varvec{t}};0.95}$*,*${{\varvec{D}}}_{{\varvec{i}},{\varvec{t}}}>0$	Low	Green

In the data analysis, we set the level of significance for weighted probabilities to be $\tau =0.05$. Furthermore, confusion matrices for classified results by the AI method with $\tau =0.05$ and $\tau =0.1$ were computed for comparisons (see Table A2 of Supplementary Material).

### Simulations studies for accuracy of the AI index

We used one of Markov Chain Monte Carlo (MCMC) algorithms, the Gibbs sampling method [25], to generate spatial–temporal observations based on the auto-Markov model (2.2). In the MCMC simulation, the coefficients estimated from the gravitrap data were considered as "true" coefficients, and 5,000 MCMC runs were generated with 4,000 runs as the burn-in period. Confusion matrices with accuracy (ACC) for comparisons of classified risk levels between the true and simulated data were computed (see Table A3 of Supplementary Material).

### The HK and kernel density estimation methods

To compare the AI index with other methods, we applied two existing indices, the HK and kernel density estimation (KDE) indices, for the gravitrap data. Specifically, for the HK index, we categorized villages into three risk groups using the criteria set by the Hong Kong government for female Aedes [24]. In the KDE method, we set the bandwidth to be 200 m in accordance with the flying distance of mosquitoes. Three risk groups were then categorized using a predefined setting in the SPARR package of the R software.

### Cross-validation analysis for risk prediction

We conducted tenfold cross validation for each week to compare performance of risk prediction by the AI, HK, and a KDE methods. The KDE method can be considered as another "dynamic" index for spatial–temporal distribution of Aedes. On the other hand, the HK index is a static index for Aedes densities, which means no spatial–temporal patterns to be considered. In the cross-validation, tenth of villages that had ADM values were randomly selected as a validation set. Then, for villages in the validation set, their ADM values were estimated by taking averages of the ADM values whose corresponding villages were in the training set and were close to the villages in the validation set. With the estimated ADM values, risk levels for villages in the validation set were computed for each method. If one village in the validation set had an estimated risk level different from the original risk level, then this village was counted as a mis-classified village.

### Results for risk maps

We used the gravitrap data of Kaohsiung in 2017 to illustrate the proposed method. For the AI method, we first used the permutation test to identify villages with high Aedes population densities. Then the Markov auto-logistic model was fitted to compute the weighted risk for each spatial–temporal site. In the AI method, the first-order neighborhood structure was considered.

### Model validation

To assess appropriateness of using the first-order neighborhood structure, we also used a second-order neighborhood structure in the AI method to analyze the gravitrap data. In Supplementary Material, Table A1 shows confusion matrices to compare analysis results by the first- and second-order neighborhood structures, and Fig A1 depicts heat plots for the second-order neighborhood structure.

We found from Table A1 and Fig A1 that the choice of neighborhood structures did not substantially influence the weighted probability of the gravitrap data (The overall ACC value was about 0.85). Particularly, in the AI method with a second-order neighborhood structure, the estimate (approximately 0.3) for the second-order neighboring effect was quite small. This result was consistent with the flying distance of mosquitoes, which is about 200 m. Additionally, Table A2 of Supplementary Material indicates that analysis results by $\tau =0.05$ and $\tau =0.1$ were the same under the first-order neighborhood structure. This may also provide an evidence that using the first-order neighborhood structure could be adequate for classification of risk levels.

### Simulation results for ACC of the AI index

We used the MCMC algorithm to simulate ${Z}_{i,t}$ and ${\theta }_{i,t}$ by the auto-Markov model based on the geographic structure of Kaohsiung. Table A3 of Supplementary Material shows the simulation result. As can be seen from Table A3, the ACC for red (risk) level was 1.0 in all weeks. For the moderate and low risk levels, values of the ACC were around 0.98. This simulation result may validate that the AI index with the auto-Markov model was reliable.

### Comparisons of analysis results by the AI, HK, and KDE methods

**Fig. 2:**
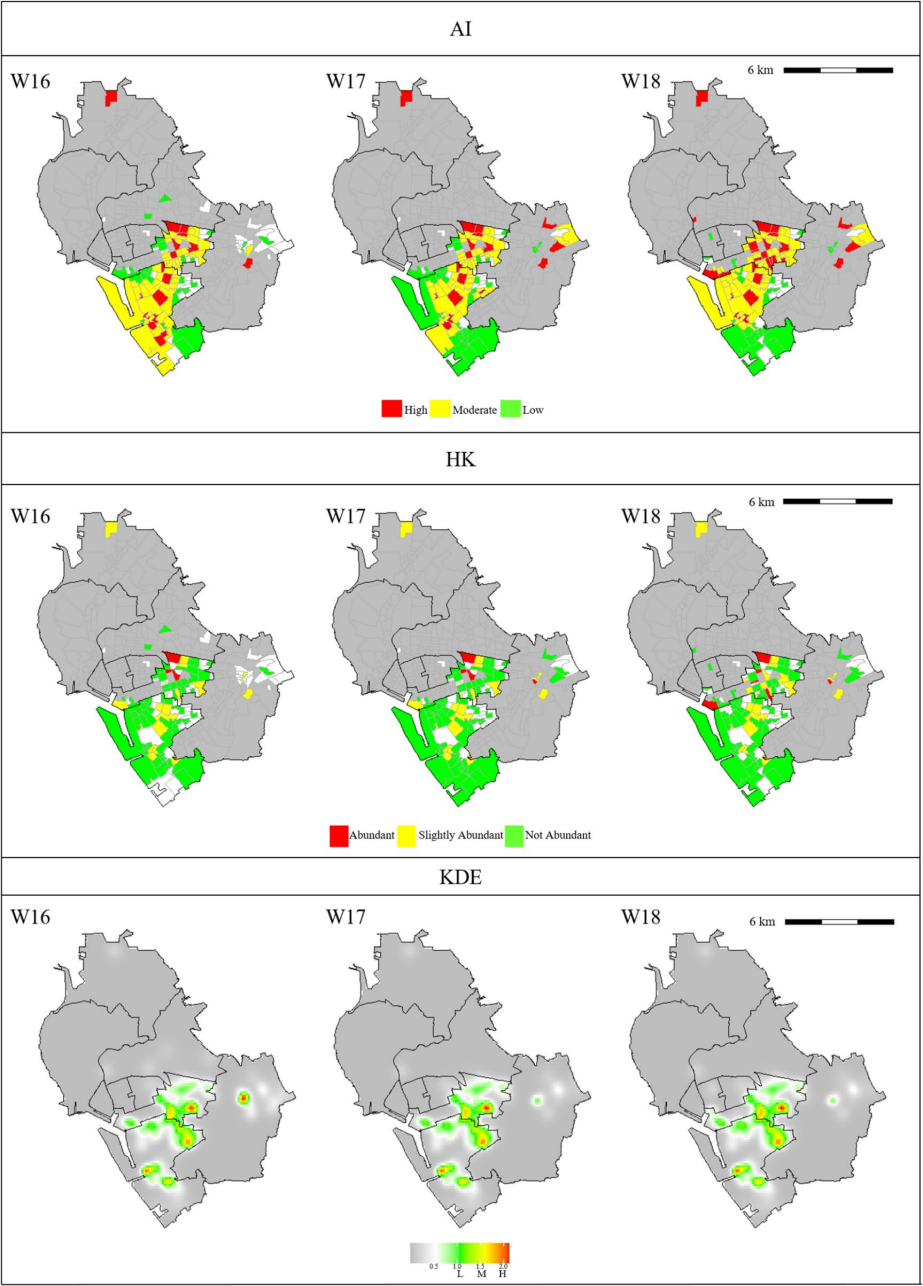

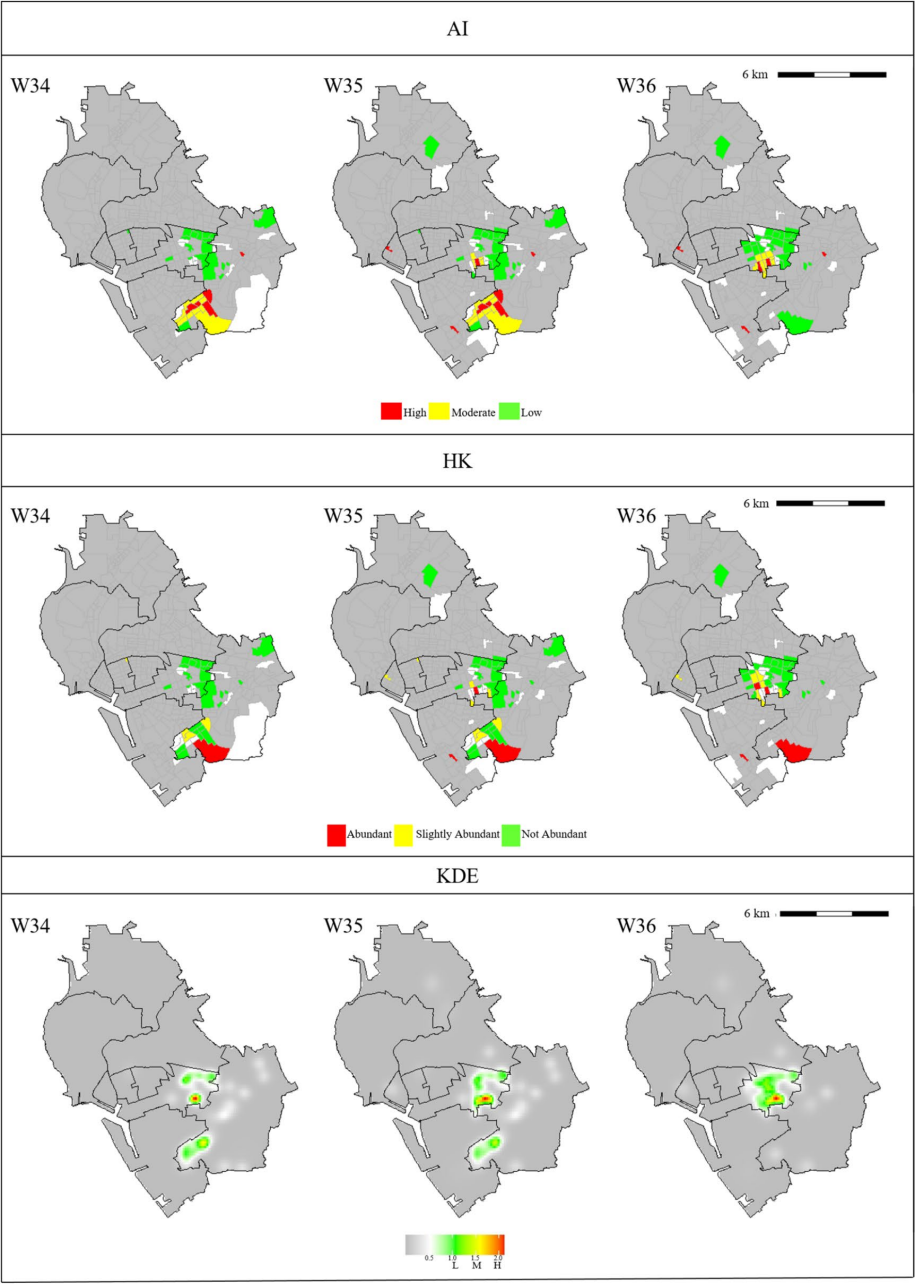

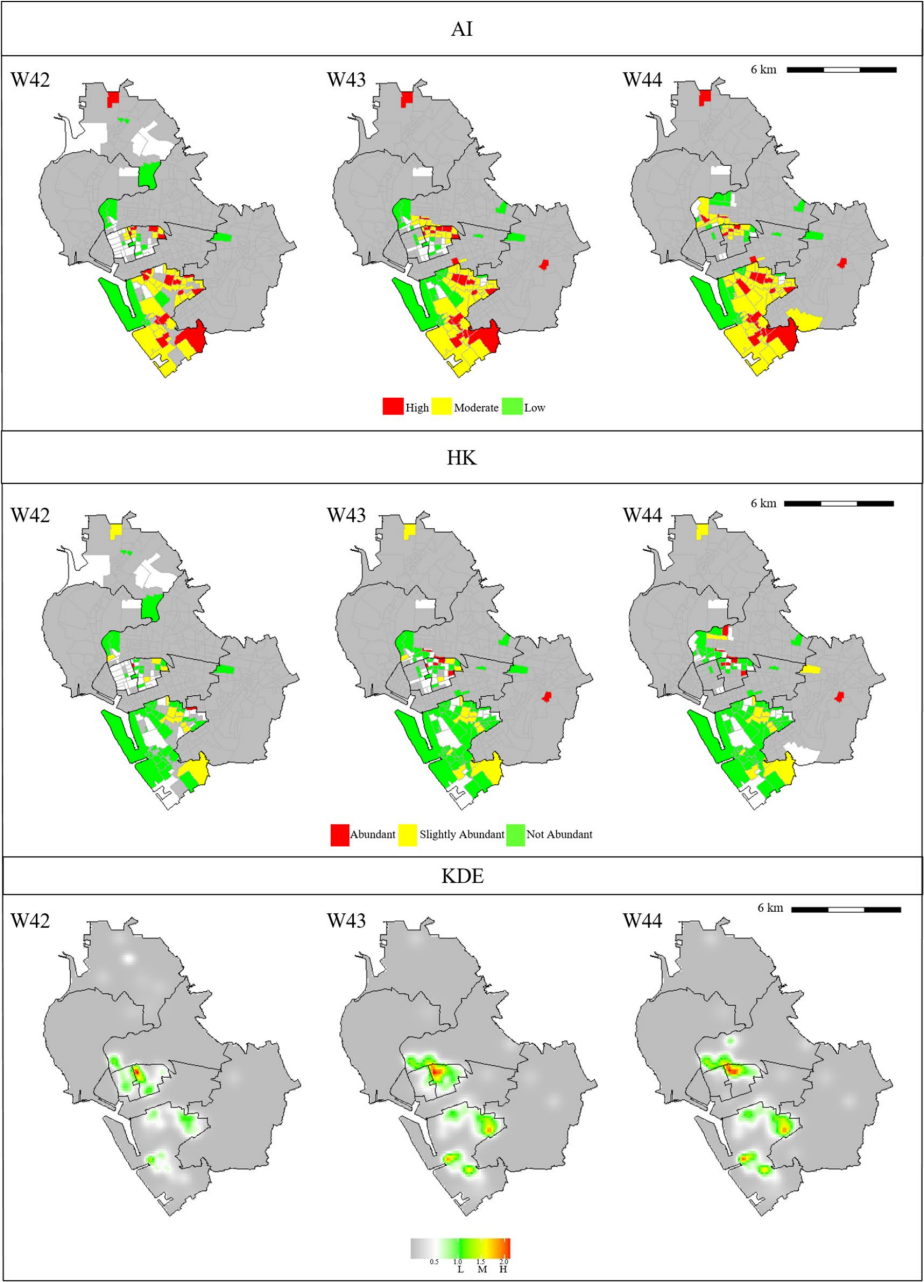
**a** Spatial–temporal patterns of risk levels computed by the AI, HK, and KDE methods for ROI 1. The time frame is from week 16 (W16) to week 18 (W18). Colors of risk levels for each method are presented in the legend. **b** Spatial–temporal patterns of risk levels computed by the AI, HK, and KDE methods for ROI 2. The time frame is from week 34 (W34) to week 36 (W36). Colors of risk levels for each method are presented in the legend. **c** Spatial–temporal patterns of risk levels computed by the AI, HK, and KDE methods for ROI 3. The time frame is from week 42 (W42) to week 44 (W44). Colors of risk levels for each method are presented in the legend

For the gravitrap data, we also used the HK and KDE methods to classify risk levels for female Aedes densities. Figure 2 depicts partial analysis results, while figures representing the whole data are shown in Fig A2 of Supplementary Material. Specifically, Fig. 2 shows heat plots for the risk groups identified by the three methods in three selected regions of interest (ROIs), ROI 1, ROI 2, and ROI 3, are shown in Fig. 2. The spatial–temporal units for ROI 1, ROI 2, and ROI 3 are 124 villages in week 16–18, 52 villages in week 34–36, and 109 villages in week 42–44, respectively. Compared to the AI and HK methods, the KDE method underestimated Aedes mosquito population densities. (Similar problems for the KDE method also happened in other bandwidths.) Moreover, the KDE method tends to smoothen the response surface neutralizing the risk levels of villages.

Compared to the HK index method, the AI method identified more villages. In ROI 1 and ROI 3, the AI method classified more villages as high- and moderate-risk than the HK index method did. However, in ROI 2, the risk classifications generated by the HK index and AI methods were similar. There were fewer villages with colors in ROI 2 than in ROI 1 and ROI 3.

### Cross-validation result for risk prediction

In the cross-validation analysis, we recorded numbers of villages that were not correctly identified into the "true" risk level. The analysis result was based on 1,000 simulation runs for each of the AI, HK, and KDE methods. The average numbers of mis-classified villages in validation sets were listed in Table A4 (for all weeks) and Table 2 (for ROIs) with sample variances. We then used two-sample *t*-tests to calculate p-values for differences of mean numbers between the three methods. Table A4 and Table 2 list p-values for the comparisons (AI versus HK and AI versus KDE). As can be seen from Table 2 and Table A4, the AI index performed significantly better than the other indices, with p-values at least less than ${10}^{-5}$ in all situations.

**Table 2:** Comparisons of risk prediction by the AI, HK, and KDE methods for the selected ROIs

	AI	HK	KDE		AI	HK	KDE		AI	HK	KDE
Week 16				Week 17				Week 18			
Mean	3.38	8.6	26	Mean	4.19	7.93	24	Mean	5.05	10.19	24
Variance	5.39	5.07	0	Variance	6.36	4.79	0	Variance	6.41	5.29	0
p-value		AI vs HK 2.25 ${\times 10}^{-38}$	AI vs KDE 8.09 ${\times 10}^{-171}$	p-value		AI vs HK 3.23 ${\times 10}^{-23}$	AI vs KDE 1.34 $\times {10}^{-152}$	p-value		AI vs HK 5.81 ${\times 10}^{-35}$	AI vs KDE 1.63 $\times {10}^{-148}$
Week 34				Week 35				Week 36			
Mean	0.67	3.2	8	Mean	1.66	5.6	12	Mean	1.15	4.57	10
Variance	0.71	1.68	0	Variance	1.7	4.73	0	Variance	1.77	4.43	0
p-value		AI vs HK 4.09 ${\times 10}^{-39}$	AI vs KDE 2.64 $\times {10}^{-161}$	p-value		AI vs HK 1.55 $\times {10}^{-36}$	AI vs KDE 2.38 $\times {10}^{-153}$	p-value		AI vs HK 5.41 $\times {10}^{-31}$	AI vs KDE 8.65 $\times {10}^{-139}$
Week 42				Week 43				Week 44			
Mean	4.41	6.22	20	Mean	5.34	7.8	24	Mean	5.99	9.15	22
Variance	4.73	4.13	0	Variance	6.19	7.03	0	Variance	4.62	6.98	0
p-value		AI vs HK 3.00 $\times {10}^{-9}$	AI vs KDE 6.49 $\times {10}^{-145}$	p-value		AI vs HK 7.12 ${\times 10}^{-11}$	AI vs KDE 1.01 $\times {10}^{-148}$	p-value		AI vs HK 1.42 ${\times 10}^{-17}$	AI vs KDE 3.74 ${\times 10}^{-148}$

The simulation result is based on tenfold cross-validation with 1,000 simulation runs for each case

Additionally, we also found that the KDE method performed significantly worse than the AI and HK indices in risk prediction. The reason for this phenomenon is because, in the training set, the KDE method could not produce a clear threshold for ADMs in each risk level. This means that, for example, the KDE method would classify two villages with a same ADM value into two different risk levels.

### Relationship between weather conditions and mosquito captures

Table 3 shows the comparisons of weather conditions and ratios of captured female Aedes in the three ROIs. The weather variables considered in our study were weekly average temperature and weekly maximum rainfall [26]. We defined daily temperature as the average of hourly temperatures per day and weekly average temperature as the average of daily temperatures in a given week. Daily rainfall was defined as the summation of hourly rainfalls over a given day, and weekly maximum rainfall was the maximum daily rainfall in a given week. The weekly ratio of captured Aedes mosquitoes was computed by dividing the number of captured individuals in a week with the total number of Aedes mosquitoes (6,768).

**Table 3:** Weather conditions, weekly average temperature (Temp) and weekly maximum rainfall (Rain), and ratio of captured Aedes mosquitoes (Ratio) between weekly and yearly data (Ratio) for the three ROIs

	ROI 1			ROI 2			ROI 3		
	W16	W17	W18	W34	W35	W36	W42	W43	W44
Ratio	3.9%	4.1%	5.4%	1.0%	1.4%	1.2%	3.1%	5.4%	5.2%
Temp (°C)	27.4	25.1	27.9	30.1	29.2	29.9	28.0	25.8	25.7
Rain (mm)	35.5	2.5	0.0	9.0	16.0	5.5	29.0	0.0	2.0

In ROI 1, ROI 2, and ROI 3, the averages of weekly average temperatures were 26.8 °C, 29.7 °C, and 26.5 °C, respectively, while the maximum values of weekly maximum rainfall were 35.5 mm, 16.0 mm, and 29.0 mm, respectively. Thus, ROI 1 and ROI 3 had more favorable weather for the growth of mosquitoes than ROI 2 did. The weekly ratios of captured female Aedes corroborate this finding. The average weekly ratios of captured female Aedes in ROI 1, ROI 2, and ROI 3 were 4.5%, 1.2%, and 4.6%, respectively. Thus, the numbers of captured Aedes mosquitoes in ROI 1 and ROI 3 were significantly higher than in ROI 2. Adjusting the weekly ratios of captured female Aedes by the number of villages, we found that the averages of the adjusted ratios were 3.6 × 10^–4^ for ROI 1, 2.3 × 10^–4^ for ROI 2, and 4.2 × 10^–4^ for ROI 3. Therefore, when weather conditions were suitable for the growth of mosquitoes, the AI index identified more high- and moderate-risk villages than the HK index did. In contrast, when the number of Aedes mosquitoes was small, the AI-based and traditional indices produced similar results.

## Discussion

Gravitrap data have been used with geographic information systems for monitoring mosquito population densities and providing a control method to reduce mosquito-borne diseases in Kaohsiung. In this study, we developed a gravitrap index using auto-model algorithms to temporally and spatially update risk levels for the severity of Aedes mosquito densities. The permutation test used in the AI index is a non-parametric test. Simulation studies based on an MCMC algorithm also validated that the AI index may upgrade traditional indices such as the HK and KDE methods.

Our AI method is capable of dynamically adjusting spatial–temporal variation by the conditional (Markov) model. So, the proposed index can be applied to cities or countries with diverse environmental conditions and distinct seasons. In Kaohsiung city, the growth of Aedes mosquitoes is affected by extreme weather conditions; high temperatures and humidity make summer suitable for the growth of Aedes mosquitoes, while the low temperatures due to continental cold air in winter could induce insect diapause. Also, the amount of rainfall in Kaohsiung changes greatly due to drought and typhoon. So, numbers of Aedes captured by gravitraps can vary considerably in different villages and seasons in Kaohsiung. Methodologically, integrating ML methods for gravitrap surveillance data also has shown strong potential for improving the mapping of Aedes aegypti populations. Recent studies have demonstrated that ML models using gravitrap-derived indices can effectively capture spatial and temporal patterns of mosquito abundance, enabling more targeted and timely vector control strategies [27]. However, the effectiveness of ML models depends heavily on the availability and consistency of high-quality trap data, which can be difficult to maintain in some regions. In addition, passive traps like gravitraps may under-sample mosquito populations in certain environments, introducing potential bias. ML models may not generalize well across different ecological settings, especially when trained on geographically limited data [14, 28]. Therefore, in our study, limited gravitrap data may have led to biased ML-derived indices; for instance, our cross-validation analyses showed that the KDE method lacked clear thresholds for classifying villages into distinct risk levels.

In terminology of spatial epidemiology, disease mapping models often refer to model for observations with positive correlation. For our gravitrap data, a Moran's I statistic [29] for testing spatial connection among ADMs gave a value of 0.023 with a p-value less than 0.001. This result provided an evidence that the Aedes density was positively correlated significantly. Therefore, it is reasonable to use the AI index, which can "borrow" information from neighboring sites to increase "resolution" for each village. The AI algorithm can efficiently use data from the vector surveillance system to compute and update risk groups based on the index criteria. This property could be useful for cities with large areas but limited resources for gravitraps deployment. In practice, the Kaohsiung city government will remove and clean Aedes breeding sites once every week, once every 2 weeks, or once every month for villages with high risk level (red), moderate risk level (yellow), or low risk level (green), respectively.

The AI and HK indices showed similar performances in identifying high-risk villages. However, for moderate-risk villages, the AI index was more effective, especially when the number of Aedes mosquitoes was relatively large. The performance of this method was also validated using the relationship between weather conditions and gravitrap indices. The AI index, being more sensitive to risk factors such as temperature, identified more high- and moderate-risk villages in areas with favorable weather conditions for mosquito proliferation. The association between weather conditions and the effectiveness of the AI-based index further emphasizes the importance of considering environmental factors in mosquito risk assessment. The findings of this study thus contribute to improved understanding of different risk assessment methodologies and their applicability in mosquito control efforts.

The study has some limitations. First, similar to an issue in most Bayesian works, the AI method associated with the Markov model is time-consuming, comparing to the KDE method. For example, using the AI method to create a risk map for 1 week could take one minute, while the KDE method only took 5 s. Second, since the AI method is associated with the disease mapping model, we found from the simulation that the AI method could over-estimate risk levels for areas with low intensities of Aedes. Third, we did not apply the same algorithm to other mosquito activity measurements, such as the Breteau index and ovitrap index, due to limited data availability. Our focus was primarily on gravitrap data because it directly represents female mosquitoes' dynamics, which are critical for assessing the risk of dengue transmission. Fourth, due to successful dengue prevention in Kaohsiung in recent years, we do not have spatial distributed dengue incidence for us to validate.

For future research, the AI index can be generalized to a Bayesian framework by using mixed effects in the auto-Markov model. Other useful works [30–32] with ML approaches can also be integrated into the Markov model to improve prediction accuracy. Future studies are required to validate how the proposed approach helps predict the disease incidence.

## Conclusions

In summary, our AI index for Aedes densities was built under the auto-Markov model, and significance of Aedes densities was evaluated by a unsupervised permutation test. Therefore, the surveillance system can dynamically adjust risk levels of Aedes for various environmental situations and seasonal change. Particaluarly, when Aedes densities were positively correlated, the AI index can be more efficient to update risk levels than other methods. Cross-validation analysis and simulation studies demonstrated that the AI index has higher sensitivity in identifying high- and moderate-risk villages compared to other methods. However, in regions with lower mosquito populations, the performance of the AI method was comparable to those of the traditional indices.

## Supplementary Information

Supplementary material 1.

## Data Availability

Data is provided within supplementary information files.

## Abbreviations

AI: Artificial intelligence
ML: Machine learning
ADM: Aedes density measure
HK: Hong Kong
MCMC: Markov Chain Monte Carlo
ACC: Accuracy
KDE: Kernel density estimation
ROI: Regions of interest
