# Supplementary material for: An AI-based gravitrap surveillance for spatial interaction analysis in predicting aedes risk
Source: Int J Health Geogr. 2025 Aug 6;24:22. doi: 10.1186/s12942-025-00403-z (PMC12329899; doi:10.1186/s12942-025-00403-z)

**Supplementary Material**

**Table A1. Confusion matrices of risk levels computed by the AI method with the first- (1st) and second-order (2nd) neighborhood structures. Notations "R", "Y", "G", and "W" are used to denote "Red", "Yellow", "Green", and "White" levels. Numbers of villages in each risk levels are presented in the table.**

| Week 7 | | | | | Week 8 | | | | |
| --- | --- | --- | --- | --- | --- | --- | --- | --- | --- |
| 1st  2nd | R | Y | G | W | 1st  2nd | R | Y | G | W |
| R | 1 | 0 | 0 | 0 | R | 1 | 0 | 0 | 0 |
| Y | 0 | 0 | 0 | 0 | Y | 0 | 0 | 0 | 0 |
| G | 0 | 0 | 11 | 0 | G | 0 | 0 | 11 | 0 |
| W | 0 | 5 | 0 | 56 | W | 0 | 5 | 0 | 57 |
| Week 9 | | | | | Week 10 | | | | |
| 1st  2nd | R | Y | G | W | 1st  2nd | R | Y | G | W |
| R | 1 | 0 | 0 | 0 | R | 1 | 0 | 0 | 0 |
| Y | 0 | 0 | 0 | 0 | Y | 0 | 0 | 0 | 0 |
| G | 0 | 0 | 14 | 0 | G | 0 | 1 | 10 | 0 |
| W | 0 | 4 | 0 | 46 | W | 0 | 0 | 0 | 21 |
| Week 11 | | | | | Week 12 | | | | |
| 1st  2nd | R | Y | G | W | 1st  2nd | R | Y | G | W |
| R | 1 | 0 | 0 | 0 | R | 3 | 0 | 0 | 0 |
| Y | 0 | 0 | 0 | 0 | Y | 0 | 0 | 3 | 0 |
| G | 0 | 2 | 21 | 0 | G | 0 | 3 | 16 | 0 |
| W | 0 | 3 | 0 | 39 | W | 0 | 11 | 0 | 37 |
| Week 13 | | | | | Week 14 | | | | |
| 1st  2nd | R | Y | G | W | 1st  2nd | R | Y | G | W |
| R | 3 | 0 | 0 | 0 | R | 2 | 0 | 0 | 0 |
| Y | 0 | 0 | 0 | 0 | Y | 0 | 0 | 0 | 0 |
| G | 0 | 2 | 23 | 0 | G | 0 | 0 | 10 | 0 |
| W | 0 | 6 | 0 | 39 | W | 0 | 1 | 0 | 23 |

| Week 15 | | | | | Week 16 | | | | |
| --- | --- | --- | --- | --- | --- | --- | --- | --- | --- |
| 1st  2nd | R | Y | G | W | 1st  2nd | R | Y | G | W |
| R | 1 | 0 | 0 | 0 | R | 15 | 0 | 0 | 0 |
| Y | 0 | 0 | 0 | 0 | Y | 0 | 32 | 8 | 5 |
| G | 0 | 0 | 6 | 0 | G | 0 | 13 | 22 | 0 |
| W | 0 | 0 | 0 | 20 | W | 0 | 4 | 0 | 31 |
| Week 17 | | | | | Week 18 | | | | |
| 1st  2nd | R | Y | G | W | 1st  2nd | R | Y | G | W |
| R | 17 | 0 | 0 | 0 | R | 26 | 0 | 0 | 0 |
| Y | 0 | 26 | 10 | 7 | Y | 0 | 40 | 6 | 6 |
| G | 0 | 16 | 24 | 0 | G | 0 | 9 | 19 | 0 |
| W | 0 | 6 | 0 | 13 | W | 0 | 5 | 0 | 13 |
| Week 19 | | | | | Week 20 | | | | |
| 1st  2nd | R | Y | G | W | 1st  2nd | R | Y | G | W |
| R | 15 | 0 | 0 | 0 | R | 4 | 0 | 0 | 0 |
| Y | 0 | 12 | 0 | 1 | Y | 0 | 0 | 0 | 0 |
| G | 0 | 2 | 7 | 0 | G | 0 | 4 | 16 | 0 |
| W | 0 | 5 | 0 | 9 | W | 0 | 3 | 0 | 29 |
| Week 21 | | | | | Week 22 | | | | |
| 1st  2nd | R | Y | G | W | 1st  2nd | R | Y | G | W |
| R | 7 | 0 | 0 | 0 | R | 6 | 0 | 0 | 0 |
| Y | 0 | 0 | 1 | 2 | Y | 0 | 0 | 1 | 2 |
| G | 0 | 8 | 18 | 0 | G | 0 | 8 | 22 | 0 |
| W | 0 | 4 | 0 | 29 | W | 0 | 4 | 0 | 29 |
| Week 23 | | | | | Week 24 | | | | |
| 1st  2nd | R | Y | G | W | 1st  2nd | R | Y | G | W |
| R | 5 | 0 | 0 | 0 | R | 5 | 0 | 0 | 0 |
| Y | 0 | 0 | 1 | 0 | Y | 0 | 0 | 0 | 2 |
| G | 0 | 2 | 15 | 0 | G | 0 | 5 | 18 | 0 |
| W | 0 | 2 | 0 | 18 | W | 0 | 3 | 0 | 22 |

| Week 25 | | | | | Week 26 | | | | |
| --- | --- | --- | --- | --- | --- | --- | --- | --- | --- |
| 1st  2nd | R | Y | G | W | 1st  2nd | R | Y | G | W |
| R | 15 | 0 | 0 | 0 | R | 17 | 0 | 0 | 0 |
| Y | 0 | 17 | 2 | 2 | Y | 0 | 20 | 3 | 1 |
| G | 0 | 8 | 6 | 0 | G | 0 | 11 | 6 | 0 |
| W | 0 | 0 | 0 | 17 | W | 0 | 1 | 0 | 17 |
| Week 27 | | | | | Week 28 | | | | |
| 1st  2nd | R | Y | G | W | 1st  2nd | R | Y | G | W |
| R | 17 | 0 | 0 | 0 | R | 9 | 0 | 0 | 0 |
| Y | 0 | 12 | 1 | 3 | Y | 0 | 6 | 7 | 1 |
| G | 0 | 11 | 6 | 0 | G | 0 | 8 | 10 | 0 |
| W | 0 | 2 | 0 | 15 | W | 0 | 5 | 0 | 21 |
| Week 29 | | | | | Week 30 | | | | |
| 1st  2nd | R | Y | G | W | 1st  2nd | R | Y | G | W |
| R | 12 | 0 | 0 | 0 | R | 10 | 0 | 0 | 0 |
|  |  |  |  |  |  |  |  |  |  |
| Y | 0 | 7 | 12 | 6 | Y | 0 | 3 | 10 | 6 |
| G | 0 | 24 | 36 | 0 | G | 0 | 16 | 38 | 0 |
| W | 0 | 12 | 0 | 33 | W | 0 | 7 | 0 | 36 |
| Week 31 | | | | | Week 32 | | | | |
| 1st  2nd | R | Y | G | W | 1st  2nd | R | Y | G | W |
| R | 7 | 0 | 0 | 0 | R | 1 | 0 | 0 | 0 |
|  |  |  |  |  |  |  |  |  |  |
| Y | 0 | 0 | 6 | 4 | Y | 0 | 0 | 0 | 0 |
| G | 0 | 13 | 34 | 0 | G | 0 | 3 | 10 | 0 |
| W | 0 | 15 | 0 | 38 | W | 0 | 1 | 0 | 28 |
| Week 33 | | | | | Week 34 | | | | |
| 1st  2nd | R | Y | G | W | 1st  2nd | R | Y | G | W |
| R | 3 | 0 | 0 | 0 | R | 5 | 0 | 0 | 0 |
| Y | 0 | 1 | 1 | 0 | Y | 0 | 7 | 0 | 2 |
| G | 0 | 9 | 10 | 0 | G | 0 | 1 | 18 | 0 |
| W | 0 | 5 | 0 | 13 | W | 0 | 1 | 0 | 10 |
| Week 35 | | | | | Week 36 | | | | |
| 1st  2nd | R | Y | G | W | 1st  2nd | R | Y | G | W |
| R | 8 | 0 | 0 | 0 | R | 5 | 0 | 0 | 0 |
| Y | 0 | 9 | 1 | 2 | Y | 0 | 0 | 2 | 0 |
| G | 0 | 2 | 20 | 0 | G | 0 | 6 | 21 | 0 |
| W | 0 | 2 | 0 | 14 | W | 0 | 3 | 0 | 16 |
| Week 37 | | | | | Week 38 | | | | |
| 1st  2nd | R | Y | G | W | 1st  2nd | R | Y | G | W |
| R | 7 | 0 | 0 | 0 | R | 8 | 0 | 0 | 0 |
| Y | 0 | 3 | 2 | 0 | Y | 0 | 9 | 2 | 0 |
| G | 0 | 8 | 16 | 0 | G | 0 | 12 | 21 | 0 |
| W | 0 | 5 | 0 | 14 | W | 0 | 3 | 0 | 11 |
| Week 39 | | | | | Week 40 | | | | |
| 1st  2nd | R | Y | G | W | 1st  2nd | R | Y | G | W |
| R | 11 | 0 | 0 | 0 | R | 9 | 0 | 0 | 0 |
| Y | 0 | 13 | 2 | 0 | Y | 0 | 15 | 3 | 2 |
| G | 0 | 8 | 20 | 0 | G | 0 | 3 | 12 | 0 |
| W | 0 | 5 | 0 | 11 | W | 0 | 3 | 0 | 21 |
|  | 11 | 26 | 22 | 11 |  | 9 | 21 | 15 | 23 |
| Week 41 | | | | | Week 42 | | | | |
| 1st  2nd | R | Y | G | W | 1st  2nd | R | Y | G | W |
| R | 5 | 0 | 0 | 0 | R | 16 | 0 | 0 | 0 |
| Y | 0 | 0 | 1 | 1 | Y | 0 | 12 | 4 | 3 |
| G | 0 | 2 | 14 | 0 | G | 0 | 15 | 14 | 0 |
| W | 0 | 3 | 0 | 33 | W | 0 | 10 | 0 | 27 |

**Table A2. Confusion matrices for risk levels conducted by the auto-Markov model with** $\boldsymbol{\tau=0.05}$ **(row) and** $\boldsymbol{\tau=0.1}$ **(column). Numbers of villages in four risk levels, red (R), yellow (Y), green (G), and white (W), with accuracy (ACC), are computed based on the gravitrap data from weeks 7 to 42 in 2027.**

| Week 7 | | | | | | Week 8 | | | | | |
| --- | --- | --- | --- | --- | --- | --- | --- | --- | --- | --- | --- |
|  | R | Y | G | W | N |  | R | Y | G | W | N |
| R | 1 | 0 | 0 | 0 | 0 | R | 1 | 0 | 0 | 0 | 0 |
| Y | 0 | 5 | 0 | 0 | 0 | Y | 0 | 5 | 0 | 0 | 0 |
| G | 0 | 0 | 11 | 0 | 0 | G | 0 | 0 | 11 | 0 | 0 |
| W | 0 | 0 | 0 | 56 | 0 | W | 0 | 0 | 0 | 57 | 0 |
| N | 0 | 0 | 0 | 0 | 367 | N | 0 | 0 | 0 | 0 | 366 |
| ACC |  |  |  |  | 100% | ACC |  |  |  |  | 100% |
| Week 9 | | | | | | Week 10 | | | | | |
|  | R | Y | G | W | N |  | R | Y | G | W | N |
| R | 1 | 0 | 0 | 0 | 0 | R | 1 | 0 | 0 | 0 | 0 |
| Y | 0 | 4 | 0 | 0 | 0 | Y | 0 | 1 | 0 | 0 | 0 |
| G | 0 | 0 | 14 | 0 | 0 | G | 0 | 0 | 10 | 0 | 0 |
| W | 0 | 0 | 0 | 46 | 0 | W | 0 | 0 | 0 | 21 | 0 |
| N | 0 | 0 | 0 | 0 | 375 | N | 0 | 0 | 0 | 0 | 407 |
| ACC |  |  |  |  | 100% | ACC |  |  |  |  | 100% |
| Week 11 | | | | | | Week 12 | | | | | |
|  | R | Y | G | W | N |  | R | Y | G | W | N |
| R | 1 | 0 | 0 | 0 | 0 | R | 3 | 0 | 0 | 0 | 0 |
| Y | 0 | 5 | 0 | 0 | 0 | Y | 0 | 14 | 0 | 0 | 0 |
| G | 0 | 0 | 21 | 0 | 0 | G | 0 | 0 | 19 | 0 | 0 |
| W | 0 | 0 | 0 | 39 | 0 | W | 0 | 0 | 0 | 37 | 0 |
| N | 0 | 0 | 0 | 0 | 374 | N | 0 | 0 | 0 | 0 | 367 |
| ACC |  |  |  |  | 100% | ACC |  |  |  |  | 100% |
| Week 13 | | | | | | Week 14 | | | | | |
|  | R | Y | G | W | N |  | R | Y | G | W | N |
| R | 3 | 0 | 0 | 0 | 0 | R | 2 | 0 | 0 | 0 | 0 |
| Y | 0 | 8 | 0 | 0 | 0 | Y | 0 | 1 | 0 | 0 | 0 |
| G | 0 | 0 | 23 | 0 | 0 | G | 0 | 0 | 10 | 0 | 0 |
| W | 0 | 0 | 0 | 39 | 0 | W | 0 | 0 | 0 | 23 | 0 |
| N | 0 | 0 | 0 | 0 | 367 | N | 0 | 0 | 0 | 0 | 404 |
| ACC |  |  |  |  | 100% | ACC |  |  |  |  | 100% |

| Week 15 | | | | | | Week 16 | | | | | |
| --- | --- | --- | --- | --- | --- | --- | --- | --- | --- | --- | --- |
|  | R | Y | G | W | N |  | R | Y | G | W | N |
| R | 1 | 0 | 0 | 0 | 0 | R | 15 | 0 | 0 | 0 | 0 |
| Y | 0 | 0 | 0 | 0 | 0 | Y | 0 | 49 | 0 | 0 | 0 |
| G | 0 | 0 | 6 | 0 | 0 | G | 0 | 0 | 30 | 0 | 0 |
| W | 0 | 0 | 0 | 20 | 0 | W | 0 | 0 | 0 | 36 | 0 |
| N | 0 | 0 | 0 | 0 | 413 | N | 0 | 0 | 0 | 0 | 310 |
| ACC |  |  |  |  | 100% | ACC |  |  |  |  | 100% |
| Week 17 | | | | | | Week 18 | | | | | |
|  | R | Y | G | W | N |  | R | Y | G | W | N |
| R | 17 | 0 | 0 | 0 | 0 | R | 26 | 0 | 0 | 0 | 0 |
| Y | 0 | 48 | 0 | 0 | 0 | Y | 0 | 54 | 0 | 0 | 0 |
| G | 0 | 0 | 34 | 0 | 0 | G | 0 | 0 | 25 | 0 | 0 |
| W | 0 | 0 | 0 | 20 | 0 | W | 0 | 0 | 0 | 19 | 0 |
| N | 0 | 0 | 0 | 0 | 321 | N | 0 | 0 | 0 | 0 | 316 |
| ACC |  |  |  |  | 100% | ACC |  |  |  |  | 100% |
| Week 19 | | | | | | Week 20 | | | | | |
|  | R | Y | G | W | N |  | R | Y | G | W | N |
| R | 15 | 0 | 0 | 0 | 0 | R | 4 | 0 | 0 | 0 | 0 |
| Y | 0 | 19 | 0 | 0 | 0 | Y | 0 | 7 | 0 | 0 | 0 |
| G | 0 | 0 | 7 | 0 | 0 | G | 0 | 0 | 16 | 0 | 0 |
| W | 0 | 0 | 0 | 10 | 0 | W | 0 | 0 | 0 | 29 | 0 |
| N | 0 | 0 | 0 | 0 | 389 | N | 0 | 0 | 0 | 0 | 384 |
| ACC |  |  |  |  | 100% | ACC |  |  |  |  | 100% |
| Week 21 | | | | | | Week 22 | | | | | |
|  | R | Y | G | W | N |  | R | Y | G | W | N |
| R | 7 | 0 | 0 | 0 | 0 | R | 6 | 0 | 0 | 0 | 0 |
| Y | 0 | 12 | 0 | 0 | 0 | Y | 0 | 12 | 0 | 0 | 0 |
| G | 0 | 0 | 19 | 0 | 0 | G | 0 | 0 | 23 | 0 | 0 |
| W | 0 | 0 | 0 | 31 | 0 | W | 0 | 0 | 0 | 31 | 0 |
| N | 0 | 0 | 0 | 0 | 371 | N | 0 | 0 | 0 | 0 | 368 |
| ACC |  |  |  |  | 100% | ACC |  |  |  |  | 100% |

| Week 23 | | | | | | Week 24 | | | | | |
| --- | --- | --- | --- | --- | --- | --- | --- | --- | --- | --- | --- |
|  | R | Y | G | W | N |  | R | Y | G | W | N |
| R | 5 | 0 | 0 | 0 | 0 | R | 5 | 0 | 0 | 0 | 0 |
| Y | 0 | 4 | 0 | 0 | 0 | Y | 0 | 8 | 0 | 0 | 0 |
| G | 0 | 0 | 16 | 0 | 0 | G | 0 | 0 | 18 | 0 | 0 |
| W | 0 | 0 | 0 | 18 | 0 | W | 0 | 0 | 0 | 24 | 0 |
| N | 0 | 0 | 0 | 0 | 397 | N | 0 | 0 | 0 | 0 | 385 |
| ACC |  |  |  |  | 100% | ACC |  |  |  |  | 100% |
| Week 25 | | | | | | Week 26 | | | | | |
|  | R | Y | G | W | N |  | R | Y | G | W | N |
| R | 15 | 0 | 0 | 0 | 0 | R | 17 | 0 | 0 | 0 | 0 |
| Y | 0 | 25 | 0 | 0 | 0 | Y | 0 | 32 | 0 | 0 | 0 |
| G | 0 | 0 | 8 | 0 | 0 | G | 0 | 0 | 9 | 0 | 0 |
| W | 0 | 0 | 0 | 19 | 0 | W | 0 | 0 | 0 | 18 | 0 |
| N | 0 | 0 | 0 | 0 | 373 | N | 0 | 0 | 0 | 0 | 364 |
| ACC |  |  |  |  | 100% | ACC |  |  |  |  | 100% |
| Week 27 | | | | | | Week 28 | | | | | |
|  | R | Y | G | W | N |  | R | Y | G | W | N |
| R | 17 | 0 | 0 | 0 | 0 | R | 9 | 0 | 0 | 0 | 0 |
| Y | 0 | 25 | 0 | 0 | 0 | Y | 0 | 19 | 0 | 0 | 0 |
| G | 0 | 0 | 7 | 0 | 0 | G | 0 | 0 | 17 | 0 | 0 |
| W | 0 | 0 | 0 | 18 | 0 | W | 0 | 0 | 0 | 22 | 0 |
| N | 0 | 0 | 0 | 0 | 373 | N | 0 | 0 | 0 | 0 | 373 |
| ACC |  |  |  |  | 100% | ACC |  |  |  |  | 100% |
| Week 29 | | | | | | Week 30 | | | | | |
|  | R | Y | G | W | N |  | R | Y | G | W | N |
| R | 12 | 0 | 0 | 0 | 0 | R | 10 | 0 | 0 | 0 | 0 |
| Y | 0 | 43 | 0 | 0 | 0 | Y | 0 | 26 | 0 | 0 | 0 |
| G | 0 | 0 | 48 | 0 | 0 | G | 0 | 0 | 48 | 0 | 0 |
| W | 0 | 0 | 0 | 39 | 0 | W | 0 | 0 | 0 | 42 | 0 |
| N | 0 | 0 | 0 | 0 | 298 | N | 0 | 0 | 0 | 0 | 314 |
| ACC |  |  |  |  | 100% | ACC |  |  |  |  | 100% |

| Week 31 | | | | | | Week 32 | | | | | |
| --- | --- | --- | --- | --- | --- | --- | --- | --- | --- | --- | --- |
|  | R | Y | G | W | N |  | R | Y | G | W | N |
| R | 7 | 0 | 0 | 0 | 0 | R | 1 | 0 | 0 | 0 | 0 |
| Y | 0 | 28 | 0 | 0 | 0 | Y | 0 | 4 | 0 | 0 | 0 |
| G | 0 | 0 | 40 | 0 | 0 | G | 0 | 0 | 10 | 0 | 0 |
| W | 0 | 0 | 0 | 42 | 0 | W | 0 | 0 | 0 | 28 | 0 |
| N | 0 | 0 | 0 | 0 | 323 | N | 0 | 0 | 0 | 0 | 397 |
| ACC |  |  |  |  | 100% | ACC |  |  |  |  | 100% |
| Week 33 | | | | | | Week 34 | | | | | |
|  | R | Y | G | W | N |  | R | Y | G | W | N |
| R | 3 | 0 | 0 | 0 | 0 | R | 5 | 0 | 0 | 0 | 0 |
| Y | 0 | 15 | 0 | 0 | 0 | Y | 0 | 9 | 0 | 0 | 0 |
| G | 0 | 0 | 11 | 0 | 0 | G | 0 | 0 | 18 | 0 | 0 |
| W | 0 | 0 | 0 | 13 | 0 | W | 0 | 0 | 0 | 12 | 0 |
| N | 0 | 0 | 0 | 0 | 398 | N | 0 | 0 | 0 | 0 | 396 |
| ACC |  |  |  |  | 100% | ACC |  |  |  |  | 100% |
| Week 35 | | | | | | Week 36 | | | | | |
|  | R | Y | G | W | N |  | R | Y | G | W | N |
| R | 8 | 0 | 0 | 0 | 0 | R | 5 | 0 | 0 | 0 | 0 |
| Y | 0 | 13 | 0 | 0 | 0 | Y | 0 | 9 | 0 | 0 | 0 |
| G | 0 | 0 | 21 | 0 | 0 | G | 0 | 0 | 23 | 0 | 0 |
| W | 0 | 0 | 0 | 16 | 0 | W | 0 | 0 | 0 | 16 | 0 |
| N | 0 | 0 | 0 | 0 | 382 | N | 0 | 0 | 0 | 0 | 387 |
| ACC |  |  |  |  | 100% | ACC |  |  |  |  | 100% |
| Week 37 | | | | | | Week 38 | | | | | |
|  | R | Y | G | W | N |  | R | Y | G | W | N |
| R | 7 | 0 | 0 | 0 | 0 | R | 8 | 0 | 0 | 0 | 0 |
| Y | 0 | 16 | 0 | 0 | 0 | Y | 0 | 24 | 0 | 0 | 0 |
| G | 0 | 0 | 18 | 0 | 0 | G | 0 | 0 | 23 | 0 | 0 |
| W | 0 | 0 | 0 | 14 | 0 | W | 0 | 0 | 0 | 11 | 0 |
| N | 0 | 0 | 0 | 0 | 385 | N | 0 | 0 | 0 | 0 | 374 |
| ACC |  |  |  |  | 100% | ACC |  |  |  |  | 100% |

| Week 39 | | | | | | Week 40 | | | | | |
| --- | --- | --- | --- | --- | --- | --- | --- | --- | --- | --- | --- |
|  | R | Y | G | W | N |  | R | Y | G | W | N |
| R | 11 | 0 | 0 | 0 | 0 | R | 9 | 0 | 0 | 0 | 0 |
| Y | 0 | 26 | 0 | 0 | 0 | Y | 0 | 21 | 0 | 0 | 0 |
| G | 0 | 0 | 22 | 0 | 0 | G | 0 | 0 | 15 | 0 | 0 |
| W | 0 | 0 | 0 | 11 | 0 | W | 0 | 0 | 0 | 23 | 0 |
| N | 0 | 0 | 0 | 0 | 370 | N | 0 | 0 | 0 | 0 | 372 |
| ACC |  |  |  |  | 100% | ACC |  |  |  |  | 100% |
| Week 41 | | | | | | Week 42 | | | | | |
|  | R | Y | G | W | N |  | R | Y | G | W | N |
| R | 5 | 0 | 0 | 0 | 0 | R | 16 | 0 | 0 | 0 | 0 |
| Y | 0 | 5 | 0 | 0 | 0 | Y | 0 | 37 | 0 | 0 | 0 |
| G | 0 | 0 | 15 | 0 | 0 | G | 0 | 0 | 18 | 0 | 0 |
| W | 0 | 0 | 0 | 34 | 0 | W | 0 | 0 | 0 | 30 | 0 |
| N | 0 | 0 | 0 | 0 | 381 | N | 0 | 0 | 0 | 0 | 339 |
| ACC |  |  |  |  | 100% | ACC |  |  |  |  | 100% |

**Table A3. Simulation studies for performance of the AI-based index by the MCMC method. (a) Comparisons for numbers of red levels between the true and MCMC methods from weeks 7 to 42.**

| Week 7 | | | | Week 8 | | | |
| --- | --- | --- | --- | --- | --- | --- | --- |
| True  MCMC | Red | Not Red |  | True  MCMC | Red | Not Red |  |
| Red | 1  (TPR:100%) | 0 |  | Red | 1  (TPR:100%) | 0 |  |
| Not Red | 0 | 439  (TNR:100%) |  | Not Red | 0 | 439  (TNR:100%) |  |
| ACC |  |  | 100% | ACC |  |  | 100% |
| Week 9 | | | | Week 10 | | | |
| True  MCMC | Red | Not Red |  | True  MCMC | Red | Not Red |  |
| Red | 1  (TPR:100%) | 0 |  | Red | 1  (TPR:100%) | 0 |  |
| Not Red | 0 | 439  (TNR:100%) |  | Not Red | 0 | 439  (TNR:100%) |  |
| ACC |  |  | 100% | ACC |  |  | 100% |
| Week 11 | | | | Week 12 | | | |
| True  MCMC | Red | Not Red |  | True  MCMC | Red | Not Red |  |
| Red | 1  (TPR:100%) | 0 |  | Red | 3  (TPR:100%) | 0 |  |
| Not Red | 0 | 439  (TNR:100%) |  | Not Red | 0 | 437  (TNR:100%) |  |
| ACC |  |  | 100% | ACC |  |  | 100% |
| Week 13 | | | | Week 14 | | | |
| True  MCMC | Red | Not Red |  | True  MCMC | Red | Not Red |  |
| Red | 3  (TPR:100%) | 0 |  | Red | 2  (TPR:100%) | 0 |  |
| Not Red | 0 | 437  (TNR:100%) |  | Not Red | 0 | 438  (TNR:100%) |  |
| ACC |  |  | 100% | ACC |  |  | 100% |

| Week 15 | | | | Week 16 | | | |
| --- | --- | --- | --- | --- | --- | --- | --- |
| True  MCMC | Red | Not Red |  | True  MCMC | Red | Not Red |  |
| Red | 1  (TPR:100%) | 0 |  | Red | 15  (TPR:100%) | 0 |  |
| Not Red | 0 | 439  (TNR:100%) |  | Not Red | 0 | 425  (TNR:100%) |  |
| ACC |  |  | 100% | ACC |  |  | 100% |
| Week 17 | | | | Week 18 | | | |
| True  MCMC | Red | Not Red |  | True  MCMC | Red | Not Red |  |
| Red | 17  (TPR:100%) | 0 |  | Red | 26  (TPR:100%) | 0 |  |
| Not Red | 0 | 423  (TNR:100%) |  | Not Red | 0 | 414  (TNR:100%) |  |
| ACC |  |  | 100% | ACC |  |  | 100% |
| Week 19 | | | | Week 20 | | | |
| True  MCMC | Red | Not Red |  | True  MCMC | Red | Not Red |  |
| Red | 15  (TPR:100%) | 0 |  | Red | 4  (TPR:100%) | 0 |  |
| Not Red | 0 | 425  (TNR:100%) |  | Not Red | 0 | 436  (TNR:100%) |  |
| ACC |  |  | 100% | ACC |  |  | 100% |
| Week 21 | | | | Week 22 | | | |
| True  MCMC | Red | Not Red |  | True  MCMC | Red | Not Red |  |
| Red | 7  (TPR:100%) | 0 |  | Red | 6  (TPR:100%) | 0 |  |
| Not Red | 0 | 433  (TNR:100%) |  | Not Red | 0 | 434  (TNR:100%) |  |
| ACC |  |  | 100% | ACC |  |  | 100% |

| Week 23 | | | | Week 24 | | | |
| --- | --- | --- | --- | --- | --- | --- | --- |
| True  MCMC | Red | Not Red |  | True  MCMC | Red | Not Red |  |
| Red | 5  (TPR:100%) | 0 |  | Red | 5  (TPR:100%) | 0 |  |
| Not Red | 0 | 435  (TNR:100%) |  | Not Red | 0 | 435  (TNR:100%) |  |
| ACC |  |  | 100% | ACC |  |  | 100% |
| Week 25 | | | | Week 26 | | | |
| True  MCMC | Red | Not Red |  | True  MCMC | Red | Not Red |  |
| Red | 15  (TPR:100%) | 0 |  | Red | 17  (TPR:100%) | 0 |  |
| Not Red | 0 | 425  (TNR:100%) |  | Not Red | 0 | 423  (TNR:100%) |  |
| ACC |  |  | 100% | ACC |  |  | 100% |
| Week 27 | | | | Week 28 | | | |
| True  MCMC | Red | Not Red |  | True  MCMC | Red | Not Red |  |
| Red | 17  (TPR:100%) | 0 |  | Red | 9  (TPR:100%) | 0 |  |
| Not Red | 0 | 423  (TNR:100%) |  | Not Red | 0 | 431  (TNR:100%) |  |
| ACC |  |  | 100% | ACC |  |  | 100% |
| Week 28 | | | | Week 30 | | | |
| True  MCMC | Red | Not Red |  | True  MCMC | Red | Not Red |  |
| Red | 12  (TPR:100%) | 0 |  | Red | 10  (TPR:100%) | 0 |  |
| Not Red | 0 | 428  (TNR:100%) |  | Not Red | 0 | 430  (TNR:100%) |  |
| ACC |  |  | 100% | ACC |  |  | 100% |

| Week 31 | | | | Week 32 | | | |
| --- | --- | --- | --- | --- | --- | --- | --- |
| True  MCMC | Red | Not Red |  | True  MCMC | Red | Not Red |  |
| Red | 7  (TPR:100%) | 0 |  | Red | 1  (TPR:100%) | 0 |  |
| Not Red | 0 | 433  (TNR:100%) |  | Not Red | 0 | 439  (TNR:100%) |  |
| ACC |  |  | 100% | ACC |  |  | 100% |
| Week 33 | | | | Week 34 | | | |
| True  MCMC | Red | Not Red |  | True  MCMC | Red | Not Red |  |
| Red | 3  (TPR:100%) | 0 |  | Red | 5  (TPR:100%) | 0 |  |
| Not Red | 0 | 437  (TNR:100%) |  | Not Red | 0 | 435  (TNR:100%) |  |
| ACC |  |  | 100% | ACC |  |  | 100% |
| Week 35 | | | | Week 36 | | | |
| True  MCMC | Red | Not Red |  | True  MCMC | Red | Not Red |  |
| Red | 8  (TPR:100%) | 0 |  | Red | 5  (TPR:100%) | 0 |  |
| Not Red | 0 | 432  (TNR:100%) |  | Not Red | 0 | 435  (TNR:100%) |  |
| ACC |  |  | 100% | ACC |  |  | 100% |
| Week 37 | | | | Week 38 | | | |
| True  MCMC | Red | Not Red |  | True  MCMC | Red | Not Red |  |
| Red | 7  (TPR:100%) | 0 |  | Red | 8  (TPR:100%) | 0 |  |
| Not Red | 0 | 433  (TNR:100%) |  | Not Red | 0 | 432(TNR:100%) |  |
| ACC |  |  | 100% | ACC |  |  | 100% |

| Week 39 | | | | Week 40 | | | |
| --- | --- | --- | --- | --- | --- | --- | --- |
| True  MCMC | Red | Not Red |  | True  MCMC | Red | Not Red |  |
| Red | 11  (TPR:100%) | 0 |  | Red | 9  (TPR:100%) | 0 |  |
| Not Red | 0 | 429  (TNR:100%) |  | Not Red | 0 | 431  (TNR:100%) |  |
| ACC |  |  | 100% | ACC |  |  | 100% |
| Week 41 | | | | Week 42 | | | |
| True  MCMC | Red | Not Red |  | True  MCMC | Red | Not Red |  |
| Red | 5  (TPR:100%) | 0 |  | Red | 16  (TPR:100%) | 0 |  |
| Not Red | 0 | 435  (TNR:100%) |  | Not Red | 0 | 424  (TNR:100%) |  |
| ACC |  |  | 100% | ACC |  |  | 100% |

**Table A3. (b) Comparisons for numbers of yellow levels between the true and MCMC methods from weeks 7 to 42.**

| Week 7 | | | | Week 8 | | | |
| --- | --- | --- | --- | --- | --- | --- | --- |
| True  MCMC | Yellow | Not Yellow |  | True  MCMC | Yellow | Not Yellow |  |
| Yellow | 5  (TPR:100%) | 23 |  | Yellow | 5  (TPR:100%) | 23 |  |
| Not Yellow | 0 | 412  (TPR:0.95) |  | Not Yellow | 0 | 412  (TPR:0.95) |  |
| ACC |  |  | 0.95 | ACC |  |  | 0.95 |
| Week 9 | | | | Week 10 | | | |
| True  MCMC | Yellow | Not Yellow |  | True  MCMC | Yellow | Not Yellow |  |
| Yellow | 4  (TPR:100%) | 18 |  | Yellow | 1  (TPR:100%) | 2 |  |
| Not Yellow | 0 | 418  (TPR:0.96) |  | Not Yellow | 0 | 437  (TPR:1) |  |
| ACC |  |  | 0.96 | ACC |  |  | 100% |
| Week 11 | | | | Week 12 | | | |
| True  MCMC | Yellow | Not Yellow |  | True  MCMC | Yellow | Not Yellow |  |
| Yellow | 5  (TPR:100%) | 18 |  | Yellow | 14  (TPR:100%) | 13 |  |
| Not Yellow | 0 | 417  (TPR:0.96) |  | Not Yellow | 0 | 413  (TPR:0.97) |  |
| ACC |  |  | 0.96 | ACC |  |  | 0.97 |
| Week 13 | | | | Week 14 | | | |
| True  MCMC | Yellow | Not Yellow |  | True  MCMC | Yellow | Not Yellow |  |
| Yellow | 8  (TPR:100%) | 17 |  | Yellow | 1  (TPR:100%) | 6 |  |
| Not Yellow | 0 | 415  (TPR:0.96) |  | Not Yellow | 0 | 433  (TPR:0.99) |  |
| ACC |  |  | 0.96 | ACC |  |  | 0.99 |

| Week 15 | | | | Week 16 | | | |
| --- | --- | --- | --- | --- | --- | --- | --- |
| True  MCMC | Yellow | Not Yellow |  | True  MCMC | Yellow | Not Yellow |  |
| Yellow | 0 | 4 |  | Yellow | 43  (TPR:0.88) | 3 |  |
| Not Yellow | 0 | 436  (TPR:0.99) |  | Not Yellow | 6 | 388  (TPR:0.99) |  |
| ACC |  |  | 0.99 | ACC |  |  | 0.98 |
| Week 17 | | | | Week 18 | | | |
| True  MCMC | Yellow | Not Yellow |  | True  MCMC | Yellow | Not Yellow |  |
| Yellow | 41  (TPR:0.85) | 3 |  | Yellow | 41  (TPR:0.76) | 0 |  |
| Not Yellow | 7 | 389  (TPR:0.99) |  | Not Yellow | 13 | 386  (TNR:100%) |  |
| ACC |  |  | 0.98 | ACC |  |  | 0.97 |
| Week 19 | | | | Week 20 | | | |
| True  MCMC | Yellow | Not Yellow |  | True  MCMC | Yellow | Not Yellow |  |
| Yellow | 9  (TPR:0.47) | 0 |  | Yellow | 7  (TPR:100%) | 8 |  |
| Not Yellow | 10 | 421  (TNR:100%) |  | Not Yellow | 0 | 425  (TPR:0.98) |  |
| ACC |  |  | 0.98 | ACC |  |  | 0.98 |
| Week 21 | | | | Week 22 | | | |
| True  MCMC | Yellow | Not Yellow |  | True  MCMC | Yellow | Not Yellow |  |
| Yellow | 11  (TPR:0.92) | 9 |  | Yellow | 12  (TPR:100%) | 12 |  |
| Not Yellow | 1 | 419  (TPR:0.98) |  | Not Yellow | 0 | 416  (TPR:0.97) |  |
| ACC |  |  | 0.98 | ACC |  |  | 0.97 |

| Week 23 | | | | Week 24 | | | |
| --- | --- | --- | --- | --- | --- | --- | --- |
| True  MCMC | Yellow | Not Yellow |  | True  MCMC | Yellow | Not Yellow |  |
| Yellow | 4  (TPR:100%) | 5 |  | Yellow | 7  (TPR:0.88) | 6 |  |
| Not Yellow | 0 | 431  (TPR:0.99) |  | Not Yellow | 1 | 426  (TPR:0.99) |  |
| ACC |  |  | 0.99 | ACC |  |  | 0.98 |
| Week 25 | | | | Week 26 | | | |
| True  MCMC | Yellow | Not Yellow |  | True  MCMC | Yellow | Not Yellow |  |
| Yellow | 16  (TPR:0.64) | 0 |  | Yellow | 19  (TPR:0.59) | 0 |  |
| Not Yellow | 9 | 415  (TNR:100%) |  | Not Yellow | 13 | 408  (TNR:100%) |  |
| ACC |  |  | 0.98 | ACC |  |  | 0.97 |
| Week 27 | | | | Week 28 | | | |
| True  MCMC | Yellow | Not Yellow |  | True  MCMC | Yellow | Not Yellow |  |
| Yellow | 14  (TPR:0.56) | 0 |  | Yellow | 15  (TPR:0.79) | 2 |  |
| Not Yellow | 11 | 415  (TNR:100%) |  | Not Yellow | 4 | 419  (TPR:0.99) |  |
| ACC |  |  | 0.98 | ACC |  |  | 0.99 |
| Week 28 | | | | Week 30 | | | |
| True  MCMC | Yellow | Not Yellow |  | True  MCMC | Yellow | Not Yellow |  |
| Yellow | 41  (TPR:0.95) | 13 |  | Yellow | 25  (TPR:0.96) | 22 |  |
| Not Yellow | 2 | 384  (TPR:0.97) |  | Not Yellow | 1 | 392  (TPR:0.95) |  |
| ACC |  |  | 0.97 | ACC |  |  | 0.95 |

| Week 31 | | | | Week 32 | | | |
| --- | --- | --- | --- | --- | --- | --- | --- |
| True  MCMC | Yellow | Not Yellow |  | True  MCMC | Yellow | Not Yellow |  |
| Yellow | 27  (TPR:0.96) | 18 |  | Yellow | 4  (TPR:100%) | 8 |  |
| Not Yellow | 1 | 394  (TPR:0.96) |  | Not Yellow | 0 | 428  (TPR:0.98) |  |
| ACC |  |  | 0.96 | ACC |  |  | 0.98 |
| Week 33 | | | | Week 34 | | | |
| True  MCMC | Yellow | Not Yellow |  | True  MCMC | Yellow | Not Yellow |  |
| Yellow | 11  (TPR:0.73) | 1 |  | Yellow | 8  (TPR:0.89) | 3 |  |
| Not Yellow | 4 | 424  (TPR:100%) |  | Not Yellow | 1 | 428  (TPR:0.99) |  |
| ACC |  |  | 0.99 | ACC |  |  | 0.99 |
| Week 35 | | | | Week 36 | | | |
| True  MCMC | Yellow | Not Yellow |  | True  MCMC | Yellow | Not Yellow |  |
| Yellow | 11  (TPR:0.85) | 3 |  | Yellow | 8  (TPR:0.89) | 7 |  |
| Not Yellow | 2 | 424  (TPR:0.99) |  | Not Yellow | 1 | 424  (TPR:0.98) |  |
| ACC |  |  | 0.99 | ACC |  |  | 0.98 |
| Week 37 | | | | Week 38 | | | |
| True  MCMC | Yellow | Not Yellow |  | True  MCMC | Yellow | Not Yellow |  |
| Yellow | 12  (TPR:0.75) | 1 |  | Yellow | 19  (TPR:0.79) | 2 |  |
| Not Yellow | 4 | 423  (TPR:1) |  | Not Yellow | 5 | 414  (TPR:100%) |  |
| ACC |  |  | 0.99 | ACC |  |  | 0.98 |

| Week 39 | | | | Week 40 | | | |
| --- | --- | --- | --- | --- | --- | --- | --- |
| True  MCMC | Yellow | Not Yellow |  | True  MCMC | Yellow | Not Yellow |  |
| Yellow | 18  (TPR:0.69) | 0 |  | Yellow | 16  (TPR:0.76) | 1 |  |
| Not Yellow | 8 | 414  (TNR:100%) |  | Not Yellow | 5 | 418  (TPR:100%) |  |
| ACC |  |  | 0.98 | ACC |  |  | 0.99 |
| Week 41 | | | | Week 42 | | | |
| True  MCMC | Yellow | Not Yellow |  | True  MCMC | Yellow | Not Yellow |  |
| Yellow | 5  (TPR:100%) | 11 |  | Yellow | 30  (TPR:0.81) | 1 |  |
| Not Yellow | 0 | 424  (TPR:0.97) |  | Not Yellow | 7 | 402  (TPR:100%) |  |
| ACC |  |  | 0.98 | ACC |  |  | 0.98 |

**Table A3. (c) Comparisons for numbers of green levels between the true and MCMC methods from weeks 7 to 42.**

| Week 7 | | | | Week 8 | | | |
| --- | --- | --- | --- | --- | --- | --- | --- |
| True  MCMC | Green | Not Green |  | True  MCMC | Green | Not Green |  |
| Green | 7  (TPR:0.64) | 0 |  | Green | 7  (TPR:0.64) | 0 |  |
| Not Green | 4 | 429  (TNR:100%) |  | Not Green | 4 | 429  (TNR:100%) |  |
| ACC |  |  | 0.99 | ACC |  |  | 0.99 |
| Week 9 | | | | Week 10 | | | |
| True  MCMC | Green | Not Green |  | True  MCMC | Green | Not Green |  |
| Green | 11  (TPR:0.79) | 0 |  | Green | 10  (TPR:100%) | 0 |  |
| Not Green | 3 | 426  (TNR:100%) |  | Not Green | 0 | 430  (TNR:100%) |  |
| ACC |  |  | 0.99 | ACC |  |  | 100% |
| Week 11 | | | | Week 12 | | | |
| True  MCMC | Green | Not Green |  | True  MCMC | Green | Not Green |  |
| Green | 15  (TPR:0.71) | 0 |  | Green | 13  (TPR:0.68) | 0 |  |
| Not Green | 6 | 419  (TNR:100%) |  | Not Green | 6 | 421  (TNR:100%) |  |
| ACC |  |  | 0.99 | ACC |  |  | :0.99 |
| Week 13 | | | | Week 14 | | | |
| True  MCMC | Green | Not Green |  | True  MCMC | Green | Not Green |  |
| Green | 16  (TPR:0.7) | 0 |  | Green | 8  (TPR:0.8) | 0 |  |
| Not Green | 7 | 417  (TNR:100%) |  | Not Green | 2 | 430  (TNR:100%) |  |
| ACC |  |  | 0.98 | ACC |  |  | 100% |

| Week 15 | | | | Week 16 | | | |
| --- | --- | --- | --- | --- | --- | --- | --- |
| True  MCMC | Green | Not Green |  | True  MCMC | Green | Not Green |  |
| Green | 5  (TPR:0.83) | 0 |  | Green | 29  (TPR:0.97) | 7 |  |
| Not Green | 1 | 434  (TNR:100%) |  | Not Green | 1 | 403  (TPR:0.98) |  |
| ACC |  |  | 100% | ACC |  |  | 0.98 |
| Week 17 | | | | Week 18 | | | |
| True  MCMC | Green | Not Green |  | True  MCMC | Green | Not Green |  |
| Green | 32  (TPR:0.94) | 5 |  | Green | 25  (TPR:100%) | 10 |  |
| Not Green | 2 | 401  (TPR:0.99) |  | Not Green | 0 | 405  (TPR:0.98) |  |
| ACC |  |  | 0.98 | ACC |  |  | 0.98 |
| Week 19 | | | | Week 20 | | | |
| True  MCMC | Green | Not Green |  | True  MCMC | Green | Not Green |  |
| Green | 7  (TPR:100%) | 5 |  | Green | 14  (TPR:0.88) | 0 |  |
| Not Green | 0 | 428  (TPR:0.99) |  | Not Green | 2 | 424  (TNR:100%) |  |
| ACC |  |  | 0.99 | ACC |  |  | 100% |
| Week 21 | | | | Week 22 | | | |
| True  MCMC | Green | Not Green |  | True  MCMC | Green | Not Green |  |
| Green | 16  (TPR:0.84) | 1 |  | Green | 19  (TPR:0.83) | 1 |  |
| Not Green | 3 | 420  (TPR:100%) |  | Not Green | 4 | 416  (TPR:100%) |  |
| ACC |  |  | 0.99 | ACC |  |  | 0.99 |

| Week 23 | | | | Week 24 | | | |
| --- | --- | --- | --- | --- | --- | --- | --- |
| True  MCMC | Green | Not Green |  | True  MCMC | Green | Not Green |  |
| Green | 14  (TPR:0.88) | 0 |  | Green | 16  (TPR:0.89) | 1 |  |
| Not Green | 2 | 424  (TNR:100%) |  | Not Green | 2 | 421  (TPR:100%) |  |
| ACC |  |  | 100% | ACC |  |  | 0.99 |
| Week 25 | | | | Week 26 | | | |
| True  MCMC | Green | Not Green |  | True  MCMC | Green | Not Green |  |
| Green | 8  (TPR:100%) | 10 |  | Green | 9  (TPR:100%) | 12 |  |
| Not Green | 0 | 422  (TPR:0.98) |  | Not Green | 0 | 419  (TPR:0.97) |  |
| ACC |  |  | ACC:0.98 | ACC |  |  | 0.97 |
| Week 27 | | | | Week 28 | | | |
| True  MCMC | Green | Not Green |  | True  MCMC | Green | Not Green |  |
| Green | 7  (TPR:100%) | 13 |  | Green | 16  (TPR:0.94) | 3 |  |
| Not Green | 0 | 420  (TPR:0.97) |  | Not Green | 1 | 420  (TPR:0.99) |  |
| ACC |  |  | 0.97 | ACC |  |  | 0.99 |
| Week 28 | | | | Week 30 | | | |
| True  MCMC | Green | Not Green |  | True  MCMC | Green | Not Green |  |
| Green | 42  (TPR:0.88) | 2 |  | Green | 36  (TPR:0.75) | 1 |  |
| Not Green | 6 | 390  (TPR:0.99) |  | Not Green | 12 | 391  (TPR:100%) |  |
| ACC |  |  | 0.98 | ACC |  |  | 0.97 |

| Week 31 | | | | Week 32 | | | |
| --- | --- | --- | --- | --- | --- | --- | --- |
| True  MCMC | Green | Not Green |  | True  MCMC | Green | Not Green |  |
| Green | 31  (TPR:0.78) | 0 |  | Green | 8  (TPR:0.8) | 0 |  |
| Not Green | 9 | 400  (TNR:100%) |  | Not Green | 2 | 430  (TNR:100%) |  |
| ACC |  |  | 0.98 | ACC |  |  | 100% |
| Week 33 | | | | Week 34 | | | |
| True  MCMC | Green | Not Green |  | True  MCMC | Green | Not Green |  |
| Green | 10  (TPR:0.91) | 3 |  | Green | 16  (TPR:0.89) | 1 |  |
| Not Green | 1 | 426  (TPR:0.99) |  | Not Green | 2 | 421  (TPR:100%) |  |
| ACC |  |  | 0.99 | ACC |  |  | 0.99 |
| Week 35 | | | | Week 36 | | | |
| True  MCMC | Green | Not Green |  | True  MCMC | Green | Not Green |  |
| Green | 19  (TPR:0.9) | 1 |  | Green | 17  (TPR:0.74) | 0 |  |
| Not Green | 2 | 418  (TPR:100%) |  | Not Green | 6 | 417  (TNR:100%) |  |
| ACC |  |  | 0.99 | ACC |  |  | 0.99 |
| Week 37 | | | | Week 38 | | | |
| True  MCMC | Green | Not Green |  | True  MCMC | Green | Not Green |  |
| Green | 17  (TPR:0.94) | 2 |  | Green | 22  (TPR:0.96) | 4 |  |
| Not Green | 1 | 420  (TPR:100%) |  | Not Green | 1 | 413  (TPR:0.99) |  |
| ACC |  |  | 0.99 | ACC |  |  | 0.99 |

| Week 39 | | | | Week 40 | | | |
| --- | --- | --- | --- | --- | --- | --- | --- |
| True  MCMC | Green | Not Green |  | True  MCMC | Green | Not Green |  |
| Green | 21  (TPR:0.95) | 5 |  | Green | 15  (TPR:100%) | 5 |  |
| Not Green | 1 | 413  (TPR:0.99) |  | Not Green | 0 | 420  (TPR:0.99) |  |
| ACC |  |  | 0.99 | ACC |  |  | 0.99 |
| Week 41 | | | | Week 42 | | | |
| True  MCMC | Green | Not Green |  | True  MCMC | Green | Not Green |  |
| Green | 12  (TPR:0.8) | 0 |  | Green | 18  (TPR:100%) | 9 |  |
| Not Green | 3 | 425  (TNR:100%) |  | Not Green | 0 | 413  (TPR:0.98) |  |
| ACC |  |  | 0.99 | ACC |  |  | 0.98 |

**Table A4. Comparisons for performance of risk prediction by the AI, HK, and KDE methods for each week. The simulation results is based on 10-fold validation with 1,000 simulation runs for each case.**

| Week 7 | | | | Week 8 | | | |
| --- | --- | --- | --- | --- | --- | --- | --- |
|  | AI | HK | KDE |  | AI | HK | KDE |
| Mean | 0.2 | 1.28 | 14 | Mean | 0.4 | 0.98 | 14 |
| Variance | 0.53 | 0.9 | 0 | Variance | 1.47 | 0.59 | 0 |
| p-value |  | AI vs HK  2.71$\times{10}^{-09}$ | AI vs KDE  6.06$\times{10}^{-115}$ | p-value |  | AI vs HK  0.002602 | AI vs KDE  2.07$\times{10}^{-92}$ |
| Week 9 | | | | Week 10 | | | |
|  | AI | HK | KDE |  | AI | HK | KDE |
| Mean | 0.22 | 1.58 | 12 | Mean | 0.38 | 1.02 | 6 |
| Variance | 0.5 | 1.06 | 0 | Variance | 0.57 | 0.63 | 0 |
| p-value |  | AI vs HK  5.41$\times{10}^{-12}$ | AI vs KDE  2.52$\times{10}^{-109}$ | p-value |  | AI vs HK  3.73$\times{10}^{-05}$ | AI vs KDE  3.95$\times{10}^{-75}$ |
| Week 11 | | | | Week 12 | | | |
|  | AI | HK | KDE |  | AI | HK | KDE |
| Mean | 0.38 | 3.8 | 14 | Mean | 0.92 | 3.1 | 14 |
| Variance | 0.98 | 2.12 | 0 | Variance | 2.77 | 2.26 | 0 |
| p-value |  | AI vs HK  4.57$\times{10}^{-25}$ | AI vs KDE  2.89$\times{10}^{-101}$ | p-value |  | AI vs HK  2.68$\times{10}^{-10}$ | AI vs KDE  2.64$\times{10}^{-77}$ |

| Week 13 | | | | Week 14 | | | |
| --- | --- | --- | --- | --- | --- | --- | --- |
|  | AI | HK | KDE |  | AI | HK | KDE |
| Mean | 0.62 | 3.36 | 14 | Mean | 0.76 | 1.36 | 8 |
| Variance | 1.51 | 2.48 | 0 | Variance | 2.06 | 1.3 | 0 |
| p-value |  | AI vs HK  2.20$\times{10}^{-16}$ | AI vs KDE  3.42$\times{10}^{-91}$ | p-value |  | AI vs HK  0.011341 | AI vs KDE  6.02$\times{10}^{-59}$ |
| Week 15 | | | | Week 16 | | | |
|  | AI | HK | KDE |  | AI | HK | KDE |
| Mean | 0.24 | 0.88 | 6 | Mean | 3.38 | 8.6 | 26 |
| Variance | 0.43 | 0.68 | 0 | Variance | 5.39 | 5.07 | 0 |
| p-value |  | AI vs HK  2.03$\times{10}^{-05}$ | AI vs KDE  6.06$\times{10}^{-82}$ | p-value |  | AI vs HK  2.25${\times10}^{-38}$ | AI vs KDE  8.09$\times{10}^{-171}$ |
| Week 17 | | | | Week 18 | | | |
|  | AI | HK | KDE |  | AI | HK | KDE |
| Mean | 4.19 | 7.93 | 24 | Mean | 5.05 | 10.19 | 24 |
| Variance | 6.36 | 4.79 | 0 | Variance | 6.41 | 5.29 | 0 |
| p-value |  | AI vs HK  3.23${\times10}^{-23}$ | AI vs KDE  1.34$\times{10}^{-152}$ | p-value |  | AI vs HK  5.81${\times10}^{-35}$ | AI vs KDE  1.63$\times{10}^{-148}$ |
| Week 19 | | | | Week 20 | | | |
|  | AI | HK | KDE |  | AI | HK | KDE |
| Mean | 3.16 | 4.62 | 10 | Mean | 0.84 | 3.56 | 12 |
| Variance | 2.18 | 2.61 | 0 | Variance | 1.93 | 3.84 | 0 |
| p-value |  | AI vs HK  3.86$\times{10}^{-6}$ | AI vs KDE  1.36$\times{10}^{-55}$ | p-value |  | AI vs HK  1.12$\times{10}^{-12}$ | AI vs KDE  3.47$\times{10}^{-78}$ |

| Week 21 | | | | Week 22 | | | |
| --- | --- | --- | --- | --- | --- | --- | --- |
|  | AI | HK | KDE |  | AI | HK | KDE |
| Mean | 1.82 | 4.68 | 14 | Mean | 1.74 | 4.76 | 14 |
| Variance | 4.11 | 4.63 | 0 | Variance | 4.65 | 3.45 | 0 |
| p-value |  | AI vs HK  3.19$\times{10}^{-10}$ | AI vs KDE  4.15$\times{10}^{-66}$ | p-value |  | AI vs HK  1.30$\times{10}^{-11}$ | AI vs KDE  7.30$\times{10}^{-64}$ |
| Week 23 | | | | Week 24 | | | |
|  | AI | HK | KDE |  | AI | HK | KDE |
| Mean | 1.42 | 2.72 | 8 | Mean | 1.24 | 4.24 | 12 |
| Variance | 2.09 | 2.53 | 0 | Variance | 2.59 | 2.59 | 0 |
| p-value |  | AI vs HK  2.16$\times{10}^{-5}$ | AI vs KDE  6.45$\times{10}^{-55}$ | p-value |  | AI vs HK  1.59$\times{10}^{-15}$ | AI vs KDE  1.69$\times{10}^{-70}$ |
| Week 25 | | | | Week 26 | | | |
|  | AI | HK | KDE |  | AI | HK | KDE |
| Mean | 3.14 | 5.22 | 14 | Mean | 4.88 | 5.54 | 16 |
| Variance | 3.02 | 5.11 | 0 | Variance | 4.8 | 4.74 | 0 |
| p-value |  | AI vs HK  6.36$\times{10}^{-7}$ | AI vs KDE  1.00$\times{10}^{-67}$ | p-value |  | AI vs HK  0.067025 | AI vs KDE  3.18$\times{10}^{-59}$ |
| Week 27 | | | | Week 28 | | | |
|  | AI | HK | KDE |  | AI | HK | KDE |
| Mean | 4.8 | 5.6 | 14 | Mean | 2.2 | 5.02 | 14 |
| Variance | 3.1 | 5.71 | 0 | Variance | 2.86 | 2.88 | 0 |
| p-value |  | AI vs HK  0.029816 | AI vs KDE  2.16$\times{10}^{-60}$ | p-value |  | AI vs HK  2.24$\times{10}^{-13}$ | AI vs KDE  2.48$\times{10}^{72}$- |

| Week 29 | | | | Week 30 | | | |
| --- | --- | --- | --- | --- | --- | --- | --- |
|  | AI | HK | KDE |  | AI | HK | KDE |
| Mean | 4.92 | 9.64 | 28 | Mean | 2.78 | 8.56 | 26 |
| Variance | 8.69 | 5.79 | 0 | Variance | 5.15 | 6.05 | 0 |
| p-valuee |  | AI vs HK  2.41$\times{10}^{-14}$ | AI vs KDE  3.82$\times{10}^{-77}$ | p-value |  | AI vs HK  7.73$\times{10}^{-22}$ | AI vs KDE  1.85$\times{10}^{-88}$ |
| Week 31 | | | | Week 32 | | | |
|  | AI | HK | KDE |  | AI | HK | KDE |
| Mean | 4.5 | 7.46 | 24 | Mean | 0.76 | 2.44 | 8 |
| Variance | 7.97 | 6.46 | 0 | Variance | 1.33 | 1.88 | 0 |
| p-value |  | AI vs HK  1.40$\times{10}^{-7}$ | AI vs KDE  6.86$\times{10}^{-72}$ | p-value |  | AI vs HK  8.75$\times{10}^{-10}$ | AI vs KDE  6.16$\times{10}^{-68}$ |
| Week 33 | | | | Week 34 | | | |
|  | AI | HK | KDE |  | AI | HK | KDE |
| Mean | 1.4 | 2.94 | 8 | Mean | 0.67 | 3.2 | 8 |
| Variance | 2.29 | 1.28 | 0 | Variance | 0.71 | 1.68 | 0 |
| p-value |  | AI vs HK  4.55$\times{10}^{-8}$ | AI vs KDE  3.18$\times{10}^{-53}$ | p-value |  | AI vs HK  4.09${\times10}^{-39}$ | AI vs KDE  2.64$\times{10}^{-161}$ |
| Week 35 | | | | Week 36 | | | |
|  | AI | HK | KDE |  | AI | HK | KDE |
| Mean | 1.66 | 5.6 | 12 | Mean | 1.15 | 4.57 | 10 |
| Variance | 1.7 | 4.73 | 0 | Variance | 1.77 | 4.43 | 0 |
| p-value |  | AI vs HK  1.55$\times{10}^{-36}$ | AI vs KDE  2.38$\times{10}^{-153}$ | p-value |  | AI vs HK  5.41$\times{10}^{-31}$ | AI vs KDE  8.65$\times{10}^{-139}$ |

| Week 37 | | | | Week 38 | | | |
| --- | --- | --- | --- | --- | --- | --- | --- |
|  | AI | HK | KDE |  | AI | HK | KDE |
| Mean | 2.08 | 5.62 | 12 | Mean | 2.12 | 4.52 | 14 |
| Variance | 4.4 | 4.69 | 0 | Variance | 3.7 | 3.15 | 0 |
| p-valuee |  | AI vs HK  2.54$\times{10}^{-13}$ | AI vs KDE  2.18$\times{10}^{-56}$ | p-value |  | AI vs HK  1.72$\times{10}^{-9}$ | AI vs KDE  3.01$\times{10}^{-67}$ |
| Week 39 | | | | Week 40 | | | |
|  | AI | HK | KDE |  | AI | HK | KDE |
| Mean | 2.8 | 5.9 | 14 | Mean | 1.98 | 4.48 | 14 |
| Variance | 3.43 | 4.17 | 0 | Variance | 1.78 | 3.15 | 0 |
| p-value |  | AI vs HK  1.45$\times{10}^{-12}$ | AI vs KDE  2.19$\times{10}^{-66}$ | p-value |  | AI vs HK  1.36$\times{10}^{-12}$ | AI vs KDE  3.98$\times{10}^{-83}$ |
| Week 41 | | | | Week 42 | | | |
|  | AI | HK | KDE |  | AI | HK | KDE |
| Mean | 1.28 | 3.68 | 12 | Mean | 4.41 | 6.22 | 20 |
| Variance | 2.08 | 3.69 | 0 | Variance | 4.73 | 4.13 | 0 |
| p-value |  | AI vs HK  1.11$\times{10}^{-10}$ | AI vs KDE  6.36$\times{10}^{-75}$ | p-value |  | AI vs HK  3.00$\times{10}^{-9}$ | AI vs KDE  6.49$\times{10}^{-145}$ |

**Table A5. Kaohsiung gravitrap data in 2017.**

| Week | Village ID | Aedes | Gravitrap | Positive Gravitrap | Week | Village ID | Aedes | Gravitrap | Positive Gravitrap |
| --- | --- | --- | --- | --- | --- | --- | --- | --- | --- |
| 7 | 6400300-030 | 0 | 20 | 0 | 7 | 6400500-052 | 0 | 21 | 0 |
| 7 | 6400300-036 | 3 | 20 | 3 | 7 | 6400500-053 | 0 | 20 | 0 |
| 7 | 6400300-037 | 0 | 10 | 0 | 7 | 6400500-054 | 0 | 2 | 0 |
| 7 | 6400300-038 | 0 | 10 | 0 | 7 | 6400500-055 | 0 | 20 | 0 |
| 7 | 6400300-039 | 1 | 20 | 1 | 7 | 6400500-056 | 0 | 20 | 0 |
| 7 | 6400300-040 | 0 | 20 | 0 | 7 | 6400500-057 | 3 | 20 | 2 |
| 7 | 6400300-041 | 0 | 20 | 0 | 7 | 6400500-058 | 0 | 20 | 0 |
| 7 | 6400300-042 | 0 | 20 | 0 | 7 | 6400500-059 | 0 | 20 | 0 |
| 7 | 6400300-043 | 0 | 20 | 0 | 7 | 6400500-060 | 1 | 20 | 1 |
| 7 | 6400500-004 | 0 | 20 | 0 | 7 | 6400500-061 | 0 | 20 | 0 |
| 7 | 6400500-006 | 0 | 20 | 0 | 7 | 6400500-062 | 0 | 20 | 0 |
| 7 | 6400500-008 | 0 | 20 | 0 | 7 | 6400500-063 | 0 | 20 | 0 |
| 7 | 6400500-020 | 0 | 20 | 0 | 7 | 6400500-064 | 2 | 40 | 2 |
| 7 | 6400500-026 | 0 | 20 | 0 | 7 | 6400500-065 | 2 | 40 | 2 |
| 7 | 6400500-028 | 0 | 20 | 0 | 7 | 6400500-066 | 0 | 20 | 0 |
| 7 | 6400500-031 | 0 | 40 | 0 | 7 | 6400500-067 | 0 | 20 | 0 |
| 7 | 6400500-032 | 0 | 20 | 0 | 7 | 6400500-068 | 0 | 20 | 0 |
| 7 | 6400500-033 | 2 | 20 | 2 | 7 | 6400500-070 | 0 | 20 | 0 |
| 7 | 6400500-034 | 0 | 20 | 0 | 7 | 6400500-072 | 0 | 1 | 0 |
| 7 | 6400500-035 | 0 | 20 | 0 | 7 | 6400500-073 | 0 | 20 | 0 |
| 7 | 6400500-036 | 2 | 20 | 2 | 7 | 6400500-074 | 0 | 20 | 0 |
| 7 | 6400500-037 | 1 | 20 | 1 | 7 | 6400500-075 | 0 | 20 | 0 |
| 7 | 6400500-038 | 0 | 20 | 0 | 7 | 6400500-076 | 0 | 19 | 0 |
| 7 | 6400500-039 | 0 | 20 | 0 | 7 | 6400500-077 | 0 | 20 | 0 |
| 7 | 6400500-040 | 0 | 20 | 0 | 7 | 6400500-078 | 0 | 20 | 0 |
| 7 | 6400500-041 | 0 | 20 | 0 | 7 | 6400500-079 | 0 | 1 | 0 |
| 7 | 6400500-042 | 0 | 20 | 0 | 7 | 6400500-081 | 0 | 20 | 0 |
| 7 | 6400500-043 | 5 | 18 | 5 | 7 | 6400500-082 | 0 | 10 | 0 |
| 7 | 6400500-044 | 0 | 20 | 0 | 7 | 6400500-083 | 0 | 10 | 0 |
| 7 | 6400500-045 | 0 | 20 | 0 | 7 | 6400500-084 | 0 | 20 | 0 |
| 7 | 6400500-046 | 0 | 20 | 0 | 7 | 6400500-085 | 0 | 20 | 0 |
| 7 | 6400500-047 | 0 | 20 | 0 | 7 | 6400500-086 | 0 | 20 | 0 |
| 7 | 6400500-048 | 0 | 20 | 0 | 7 | 6400500-087 | 0 | 20 | 0 |
| 7 | 6400500-049 | 0 | 1 | 0 | 7 | 6400500-088 | 0 | 20 | 0 |
| 7 | 6400500-051 | 0 | 20 | 0 | 7 | 6400900-031 | 0 | 1 | 0 |
| 7 | 6400900-045 | 1 | 20 | 1 | 8 | 6400500-053 | 0 | 20 | 0 |
| 7 | 6400900-048 | 3 | 20 | 2 | 8 | 6400500-054 | 0 | 18 | 0 |
| 7 | 6401200-033 | 0 | 20 | 0 | 8 | 6400500-055 | 0 | 20 | 0 |
| 8 | 6400300-040 | 0 | 20 | 0 | 8 | 6400500-056 | 0 | 20 | 0 |
| 8 | 6400300-041 | 0 | 20 | 0 | 8 | 6400500-057 | 3 | 20 | 2 |
| 8 | 6400300-042 | 0 | 20 | 0 | 8 | 6400500-058 | 0 | 20 | 0 |
| 8 | 6400300-043 | 0 | 20 | 0 | 8 | 6400500-059 | 0 | 20 | 0 |
| 8 | 6400500-004 | 0 | 20 | 0 | 8 | 6400500-060 | 1 | 20 | 1 |
| 8 | 6400500-006 | 0 | 20 | 0 | 8 | 6400500-061 | 0 | 20 | 0 |
| 8 | 6400500-008 | 0 | 20 | 0 | 8 | 6400500-062 | 0 | 20 | 0 |
| 8 | 6400500-020 | 0 | 20 | 0 | 8 | 6400500-063 | 0 | 20 | 0 |
| 8 | 6400500-022 | 1 | 20 | 1 | 8 | 6400500-064 | 2 | 40 | 2 |
| 8 | 6400500-026 | 0 | 20 | 0 | 8 | 6400500-065 | 2 | 40 | 2 |
| 8 | 6400500-028 | 0 | 20 | 0 | 8 | 6400500-066 | 0 | 20 | 0 |
| 8 | 6400500-031 | 0 | 40 | 0 | 8 | 6400500-067 | 0 | 20 | 0 |
| 8 | 6400500-032 | 0 | 20 | 0 | 8 | 6400500-068 | 0 | 20 | 0 |
| 8 | 6400500-033 | 2 | 20 | 2 | 8 | 6400500-069 | 0 | 20 | 0 |
| 8 | 6400500-034 | 0 | 20 | 0 | 8 | 6400500-070 | 0 | 20 | 0 |
| 8 | 6400500-035 | 0 | 20 | 0 | 8 | 6400500-072 | 0 | 19 | 0 |
| 8 | 6400500-036 | 2 | 20 | 2 | 8 | 6400500-073 | 0 | 20 | 0 |
| 8 | 6400500-037 | 1 | 20 | 1 | 8 | 6400500-074 | 0 | 20 | 0 |
| 8 | 6400500-038 | 0 | 20 | 0 | 8 | 6400500-075 | 0 | 20 | 0 |
| 8 | 6400500-039 | 0 | 20 | 0 | 8 | 6400500-076 | 0 | 19 | 0 |
| 8 | 6400500-040 | 0 | 20 | 0 | 8 | 6400500-077 | 0 | 20 | 0 |
| 8 | 6400500-041 | 0 | 20 | 0 | 8 | 6400500-078 | 0 | 20 | 0 |
| 8 | 6400500-042 | 0 | 20 | 0 | 8 | 6400500-079 | 0 | 19 | 0 |
| 8 | 6400500-043 | 5 | 18 | 5 | 8 | 6400500-081 | 0 | 20 | 0 |
| 8 | 6400500-044 | 0 | 20 | 0 | 8 | 6400500-082 | 0 | 10 | 0 |
| 8 | 6400500-045 | 0 | 20 | 0 | 8 | 6400500-083 | 0 | 10 | 0 |
| 8 | 6400500-046 | 0 | 20 | 0 | 8 | 6400500-084 | 0 | 20 | 0 |
| 8 | 6400500-047 | 0 | 20 | 0 | 8 | 6400500-085 | 0 | 20 | 0 |
| 8 | 6400500-048 | 0 | 20 | 0 | 8 | 6400500-086 | 0 | 20 | 0 |
| 8 | 6400500-049 | 1 | 19 | 1 | 8 | 6400500-087 | 0 | 20 | 0 |
| 8 | 6400500-050 | 0 | 20 | 0 | 8 | 6400500-088 | 0 | 20 | 0 |
| 8 | 6400500-051 | 0 | 20 | 0 | 8 | 6400900-003 | 0 | 2 | 0 |
| 8 | 6400500-052 | 0 | 21 | 0 | 8 | 6400900-008 | 1 | 20 | 1 |
| 8 | 6400900-022 | 0 | 2 | 0 | 9 | 6400500-065 | 2 | 40 | 2 |
| 8 | 6400900-031 | 0 | 1 | 0 | 9 | 6400500-066 | 0 | 20 | 0 |
| 8 | 6400900-045 | 1 | 20 | 1 | 9 | 6400500-067 | 0 | 20 | 0 |
| 8 | 6400900-051 | 0 | 2 | 0 | 9 | 6400500-068 | 0 | 20 | 0 |
| 8 | 6401200-033 | 0 | 20 | 0 | 9 | 6400500-069 | 0 | 20 | 0 |
| 8 | 6400500-058 | 0 | 20 | 0 | 9 | 6400500-070 | 0 | 20 | 0 |
| 9 | 6400200-016 | 2 | 20 | 2 | 9 | 6400500-072 | 0 | 19 | 0 |
| 9 | 6400300-040 | 0 | 20 | 0 | 9 | 6400500-073 | 0 | 20 | 0 |
| 9 | 6400500-004 | 0 | 20 | 0 | 9 | 6400500-074 | 0 | 20 | 0 |
| 9 | 6400500-013 | 0 | 20 | 0 | 9 | 6400500-075 | 0 | 20 | 0 |
| 9 | 6400500-022 | 1 | 20 | 1 | 9 | 6400500-076 | 0 | 19 | 0 |
| 9 | 6400500-033 | 2 | 20 | 2 | 9 | 6400500-077 | 0 | 20 | 0 |
| 9 | 6400500-034 | 0 | 20 | 0 | 9 | 6400500-078 | 0 | 20 | 0 |
| 9 | 6400500-035 | 0 | 20 | 0 | 9 | 6400500-079 | 0 | 19 | 0 |
| 9 | 6400500-036 | 2 | 20 | 2 | 9 | 6400500-081 | 0 | 20 | 0 |
| 9 | 6400500-042 | 0 | 20 | 0 | 9 | 6400500-082 | 0 | 10 | 0 |
| 9 | 6400500-043 | 5 | 18 | 5 | 9 | 6400500-083 | 0 | 10 | 0 |
| 9 | 6400500-046 | 0 | 20 | 0 | 9 | 6400500-084 | 0 | 20 | 0 |
| 9 | 6400500-047 | 0 | 20 | 0 | 9 | 6400500-085 | 0 | 20 | 0 |
| 9 | 6400500-048 | 0 | 20 | 0 | 9 | 6400500-086 | 0 | 20 | 0 |
| 9 | 6400500-049 | 1 | 19 | 1 | 9 | 6400500-087 | 0 | 20 | 0 |
| 9 | 6400500-050 | 0 | 20 | 0 | 9 | 6400500-088 | 0 | 20 | 0 |
| 9 | 6400500-051 | 0 | 20 | 0 | 9 | 6400900-003 | 0 | 2 | 0 |
| 9 | 6400500-052 | 0 | 19 | 0 | 9 | 6400900-004 | 0 | 1 | 0 |
| 9 | 6400500-053 | 0 | 20 | 0 | 9 | 6400900-008 | 1 | 20 | 1 |
| 9 | 6400500-054 | 0 | 18 | 0 | 9 | 6400900-010 | 1 | 20 | 1 |
| 9 | 6400500-055 | 0 | 20 | 0 | 9 | 6400900-021 | 0 | 20 | 0 |
| 9 | 6400500-056 | 0 | 20 | 0 | 9 | 6400900-022 | 0 | 2 | 0 |
| 9 | 6400500-057 | 3 | 20 | 2 | 9 | 6400900-031 | 0 | 1 | 0 |
| 9 | 6400500-058 | 0 | 20 | 0 | 9 | 6400900-032 | 1 | 20 | 1 |
| 9 | 6400500-059 | 0 | 20 | 0 | 9 | 6400900-044 | 0 | 1 | 0 |
| 9 | 6400500-060 | 1 | 20 | 1 | 9 | 6400900-045 | 1 | 20 | 1 |
| 9 | 6400500-061 | 0 | 20 | 0 | 9 | 6400900-051 | 0 | 2 | 0 |
| 9 | 6400500-062 | 0 | 20 | 0 | 9 | 6400900-055 | 1 | 20 | 1 |
| 9 | 6400500-063 | 0 | 20 | 0 | 9 | 6401200-033 | 0 | 20 | 0 |
| 9 | 6400500-064 | 2 | 40 | 2 | 10 | 6400200-016 | 2 | 20 | 2 |
| 10 | 6400300-017 | 1 | 20 | 1 | 11 | 6400900-004 | 0 | 1 | 0 |
| 10 | 6400500-013 | 0 | 20 | 0 | 11 | 6400900-010 | 1 | 20 | 1 |
| 10 | 6400500-022 | 1 | 20 | 1 | 11 | 6400900-014 | 0 | 2 | 0 |
| 10 | 6400500-050 | 0 | 20 | 0 | 11 | 6400900-015 | 0 | 1 | 0 |
| 10 | 6400500-052 | 0 | 19 | 0 | 11 | 6400900-021 | 0 | 20 | 0 |
| 10 | 6400500-069 | 0 | 20 | 0 | 11 | 6400900-022 | 2 | 18 | 2 |
| 10 | 6400800-030 | 0 | 20 | 0 | 11 | 6400900-029 | 0 | 1 | 0 |
| 10 | 6400900-003 | 0 | 2 | 0 | 11 | 6400900-032 | 1 | 20 | 1 |
| 10 | 6400900-004 | 0 | 1 | 0 | 11 | 6400900-039 | 0 | 1 | 0 |
| 10 | 6400900-008 | 1 | 20 | 1 | 11 | 6400900-044 | 0 | 1 | 0 |
| 10 | 6400900-010 | 1 | 20 | 1 | 11 | 6400900-046 | 0 | 1 | 0 |
| 10 | 6400900-014 | 0 | 2 | 0 | 11 | 6400900-050 | 0 | 1 | 0 |
| 10 | 6400900-015 | 0 | 1 | 0 | 11 | 6400900-054 | 0 | 1 | 0 |
| 10 | 6400900-021 | 0 | 20 | 0 | 11 | 6400900-055 | 1 | 20 | 1 |
| 10 | 6400900-022 | 0 | 2 | 0 | 11 | 6401200-004 | 0 | 20 | 0 |
| 10 | 6400900-031 | 0 | 1 | 0 | 11 | 6401200-005 | 1 | 20 | 1 |
| 10 | 6400900-032 | 1 | 20 | 1 | 11 | 6401200-006 | 6 | 40 | 4 |
| 10 | 6400900-044 | 0 | 1 | 0 | 11 | 6401200-008 | 0 | 20 | 0 |
| 10 | 6400900-046 | 0 | 1 | 0 | 11 | 6401200-012 | 0 | 1 | 0 |
| 10 | 6400900-051 | 0 | 2 | 0 | 11 | 6401200-013 | 0 | 20 | 0 |
| 10 | 6400900-055 | 1 | 20 | 1 | 11 | 6401200-016 | 0 | 20 | 0 |
| 10 | 6401200-005 | 1 | 20 | 1 | 11 | 6401200-017 | 3 | 20 | 1 |
| 10 | 6401200-026 | 1 | 20 | 1 | 11 | 6401200-019 | 3 | 20 | 3 |
| 10 | 6401200-033 | 0 | 20 | 0 | 11 | 6401200-020 | 0 | 20 | 0 |
| 10 | 6401200-045 | 6 | 20 | 5 | 11 | 6401200-025 | 0 | 20 | 0 |
| 10 | 6401200-049 | 0 | 1 | 0 | 11 | 6401200-026 | 1 | 20 | 1 |
| 10 | 6401200-054 | 0 | 1 | 0 | 11 | 6401200-027 | 0 | 20 | 0 |
| 10 | 6401200-062 | 1 | 20 | 1 | 11 | 6401200-028 | 1 | 20 | 1 |
| 10 | 6401200-064 | 0 | 20 | 0 | 11 | 6401200-029 | 0 | 20 | 0 |
| 10 | 6401200-065 | 0 | 3 | 0 | 11 | 6401200-032 | 0 | 20 | 0 |
| 10 | 6401200-075 | 0 | 1 | 0 | 11 | 6401200-033 | 0 | 20 | 0 |
| 10 | 6401200-076 | 1 | 20 | 1 | 11 | 6401200-034 | 0 | 20 | 0 |
| 11 | 6400200-016 | 2 | 20 | 2 | 11 | 6401200-036 | 1 | 20 | 1 |
| 11 | 6400300-017 | 1 | 20 | 1 | 11 | 6401200-037 | 0 | 20 | 0 |
| 11 | 6400500-013 | 0 | 20 | 0 | 11 | 6401200-038 | 0 | 20 | 0 |
| 11 | 6400800-030 | 0 | 20 | 0 | 11 | 6401200-039 | 0 | 1 | 0 |
| 11 | 6401200-040 | 0 | 20 | 0 | 12 | 6400900-039 | 0 | 1 | 0 |
| 11 | 6401200-042 | 1 | 20 | 1 | 12 | 6400900-040 | 0 | 1 | 0 |
| 11 | 6401200-043 | 0 | 20 | 0 | 12 | 6400900-046 | 0 | 1 | 0 |
| 11 | 6401200-045 | 6 | 20 | 5 | 12 | 6400900-050 | 0 | 1 | 0 |
| 11 | 6401200-046 | 0 | 20 | 0 | 12 | 6400900-054 | 0 | 1 | 0 |
| 11 | 6401200-049 | 0 | 1 | 0 | 12 | 6401200-004 | 0 | 20 | 0 |
| 11 | 6401200-054 | 0 | 1 | 0 | 12 | 6401200-005 | 0 | 20 | 0 |
| 11 | 6401200-055 | 0 | 20 | 0 | 12 | 6401200-006 | 6 | 40 | 4 |
| 11 | 6401200-057 | 0 | 20 | 0 | 12 | 6401200-008 | 0 | 20 | 0 |
| 11 | 6401200-062 | 1 | 20 | 1 | 12 | 6401200-009 | 2 | 20 | 2 |
| 11 | 6401200-063 | 3 | 20 | 1 | 12 | 6401200-011 | 0 | 20 | 0 |
| 11 | 6401200-064 | 0 | 20 | 0 | 12 | 6401200-012 | 0 | 1 | 0 |
| 11 | 6401200-065 | 0 | 3 | 0 | 12 | 6401200-013 | 0 | 20 | 0 |
| 11 | 6401200-066 | 1 | 20 | 1 | 12 | 6401200-016 | 0 | 20 | 0 |
| 11 | 6401200-067 | 0 | 20 | 0 | 12 | 6401200-017 | 3 | 20 | 1 |
| 11 | 6401200-068 | 2 | 20 | 1 | 12 | 6401200-019 | 3 | 20 | 3 |
| 11 | 6401200-069 | 1 | 20 | 1 | 12 | 6401200-020 | 0 | 20 | 0 |
| 11 | 6401200-070 | 2 | 20 | 2 | 12 | 6401200-024 | 1 | 20 | 1 |
| 11 | 6401200-071 | 1 | 20 | 1 | 12 | 6401200-025 | 0 | 20 | 0 |
| 11 | 6401200-072 | 0 | 20 | 0 | 12 | 6401200-026 | 1 | 20 | 1 |
| 11 | 6401200-073 | 0 | 20 | 0 | 12 | 6401200-027 | 0 | 20 | 0 |
| 11 | 6401200-074 | 0 | 20 | 0 | 12 | 6401200-028 | 1 | 20 | 1 |
| 11 | 6401200-075 | 0 | 1 | 0 | 12 | 6401200-029 | 0 | 20 | 0 |
| 11 | 6401200-076 | 1 | 20 | 1 | 12 | 6401200-030 | 0 | 2 | 0 |
| 11 | 6401200-077 | 2 | 20 | 2 | 12 | 6401200-032 | 0 | 20 | 0 |
| 11 | 6401200-078 | 0 | 20 | 0 | 12 | 6401200-033 | 0 | 20 | 0 |
| 12 | 6400300-017 | 11 | 20 | 4 | 12 | 6401200-034 | 0 | 20 | 0 |
| 12 | 6400800-030 | 0 | 20 | 0 | 12 | 6401200-036 | 1 | 20 | 1 |
| 12 | 6400900-004 | 0 | 3 | 0 | 12 | 6401200-037 | 0 | 20 | 0 |
| 12 | 6400900-005 | 0 | 1 | 0 | 12 | 6401200-038 | 0 | 20 | 0 |
| 12 | 6400900-013 | 0 | 1 | 0 | 12 | 6401200-039 | 0 | 1 | 0 |
| 12 | 6400900-014 | 0 | 18 | 0 | 12 | 6401200-040 | 0 | 20 | 0 |
| 12 | 6400900-015 | 0 | 1 | 0 | 12 | 6401200-042 | 1 | 20 | 1 |
| 12 | 6400900-022 | 2 | 18 | 2 | 12 | 6401200-043 | 0 | 20 | 0 |
| 12 | 6400900-024 | 0 | 1 | 0 | 12 | 6401200-044 | 0 | 20 | 0 |
| 12 | 6400900-029 | 0 | 1 | 0 | 12 | 6401200-045 | 6 | 20 | 5 |
| 12 | 6401200-046 | 0 | 20 | 0 | 13 | 6400900-024 | 0 | 1 | 0 |
| 12 | 6401200-047 | 5 | 20 | 3 | 13 | 6400900-029 | 0 | 1 | 0 |
| 12 | 6401200-048 | 1 | 20 | 1 | 13 | 6400900-039 | 0 | 1 | 0 |
| 12 | 6401200-049 | 0 | 19 | 0 | 13 | 6400900-040 | 0 | 1 | 0 |
| 12 | 6401200-054 | 1 | 19 | 1 | 13 | 6400900-046 | 0 | 1 | 0 |
| 12 | 6401200-055 | 0 | 20 | 0 | 13 | 6400900-050 | 0 | 1 | 0 |
| 12 | 6401200-056 | 1 | 20 | 1 | 13 | 6400900-054 | 0 | 1 | 0 |
| 12 | 6401200-057 | 0 | 20 | 0 | 13 | 6401200-004 | 0 | 20 | 0 |
| 12 | 6401200-059 | 0 | 20 | 0 | 13 | 6401200-005 | 0 | 20 | 0 |
| 12 | 6401200-060 | 0 | 20 | 0 | 13 | 6401200-006 | 6 | 40 | 4 |
| 12 | 6401200-062 | 0 | 20 | 0 | 13 | 6401200-008 | 0 | 20 | 0 |
| 12 | 6401200-063 | 3 | 20 | 1 | 13 | 6401200-009 | 2 | 20 | 2 |
| 12 | 6401200-064 | 0 | 20 | 0 | 13 | 6401200-010 | 0 | 1 | 0 |
| 12 | 6401200-065 | 1 | 17 | 1 | 13 | 6401200-011 | 0 | 20 | 0 |
| 12 | 6401200-066 | 1 | 20 | 1 | 13 | 6401200-012 | 0 | 19 | 0 |
| 12 | 6401200-067 | 0 | 20 | 0 | 13 | 6401200-013 | 0 | 20 | 0 |
| 12 | 6401200-068 | 2 | 20 | 1 | 13 | 6401200-016 | 0 | 20 | 0 |
| 12 | 6401200-069 | 1 | 20 | 1 | 13 | 6401200-017 | 3 | 20 | 1 |
| 12 | 6401200-070 | 2 | 20 | 2 | 13 | 6401200-019 | 3 | 20 | 3 |
| 12 | 6401200-071 | 1 | 20 | 1 | 13 | 6401200-020 | 0 | 20 | 0 |
| 12 | 6401200-072 | 0 | 20 | 0 | 13 | 6401200-024 | 1 | 20 | 1 |
| 12 | 6401200-073 | 0 | 20 | 0 | 13 | 6401200-025 | 0 | 20 | 0 |
| 12 | 6401200-074 | 0 | 20 | 0 | 13 | 6401200-027 | 0 | 20 | 0 |
| 12 | 6401200-075 | 2 | 19 | 2 | 13 | 6401200-028 | 1 | 20 | 1 |
| 12 | 6401200-076 | 0 | 20 | 0 | 13 | 6401200-029 | 0 | 20 | 0 |
| 12 | 6401200-077 | 2 | 20 | 2 | 13 | 6401200-030 | 0 | 2 | 0 |
| 12 | 6401200-078 | 0 | 20 | 0 | 13 | 6401200-031 | 4 | 20 | 2 |
| 13 | 6400300-017 | 11 | 20 | 4 | 13 | 6401200-032 | 0 | 20 | 0 |
| 13 | 6400500-051 | 1 | 20 | 1 | 13 | 6401200-034 | 0 | 20 | 0 |
| 13 | 6400600-002 | 1 | 20 | 1 | 13 | 6401200-036 | 1 | 20 | 1 |
| 13 | 6400800-039 | 5 | 20 | 3 | 13 | 6401200-037 | 0 | 20 | 0 |
| 13 | 6400900-004 | 0 | 1 | 0 | 13 | 6401200-038 | 0 | 20 | 0 |
| 13 | 6400900-005 | 0 | 1 | 0 | 13 | 6401200-039 | 0 | 19 | 0 |
| 13 | 6400900-013 | 0 | 1 | 0 | 13 | 6401200-040 | 0 | 20 | 0 |
| 13 | 6400900-014 | 0 | 18 | 0 | 13 | 6401200-042 | 1 | 20 | 1 |
| 13 | 6400900-022 | 2 | 18 | 2 | 13 | 6401200-043 | 0 | 20 | 0 |
| 13 | 6401200-044 | 0 | 20 | 0 | 14 | 6400900-005 | 0 | 1 | 0 |
| 13 | 6401200-046 | 0 | 20 | 0 | 14 | 6400900-013 | 0 | 1 | 0 |
| 13 | 6401200-047 | 5 | 20 | 3 | 14 | 6400900-024 | 0 | 1 | 0 |
| 13 | 6401200-048 | 1 | 20 | 1 | 14 | 6400900-040 | 0 | 1 | 0 |
| 13 | 6401200-049 | 0 | 19 | 0 | 14 | 6400900-046 | 0 | 1 | 0 |
| 13 | 6401200-053 | 1 | 20 | 1 | 14 | 6401200-007 | 0 | 20 | 0 |
| 13 | 6401200-054 | 1 | 19 | 1 | 14 | 6401200-009 | 2 | 20 | 2 |
| 13 | 6401200-055 | 0 | 20 | 0 | 14 | 6401200-010 | 0 | 1 | 0 |
| 13 | 6401200-056 | 1 | 20 | 1 | 14 | 6401200-011 | 0 | 20 | 0 |
| 13 | 6401200-057 | 0 | 20 | 0 | 14 | 6401200-012 | 0 | 19 | 0 |
| 13 | 6401200-059 | 0 | 20 | 0 | 14 | 6401200-021 | 0 | 20 | 0 |
| 13 | 6401200-060 | 0 | 20 | 0 | 14 | 6401200-024 | 1 | 20 | 1 |
| 13 | 6401200-062 | 0 | 20 | 0 | 14 | 6401200-030 | 0 | 18 | 0 |
| 13 | 6401200-063 | 3 | 20 | 1 | 14 | 6401200-031 | 4 | 20 | 2 |
| 13 | 6401200-065 | 1 | 17 | 1 | 14 | 6401200-035 | 0 | 20 | 0 |
| 13 | 6401200-066 | 1 | 20 | 1 | 14 | 6401200-039 | 0 | 19 | 0 |
| 13 | 6401200-067 | 0 | 20 | 0 | 14 | 6401200-041 | 0 | 20 | 0 |
| 13 | 6401200-068 | 2 | 20 | 1 | 14 | 6401200-044 | 0 | 20 | 0 |
| 13 | 6401200-069 | 1 | 20 | 1 | 14 | 6401200-045 | 0 | 10 | 0 |
| 13 | 6401200-070 | 2 | 20 | 2 | 14 | 6401200-047 | 5 | 20 | 3 |
| 13 | 6401200-071 | 1 | 20 | 1 | 14 | 6401200-048 | 1 | 20 | 1 |
| 13 | 6401200-072 | 0 | 20 | 0 | 14 | 6401200-050 | 0 | 20 | 0 |
| 13 | 6401200-073 | 0 | 20 | 0 | 14 | 6401200-051 | 0 | 20 | 0 |
| 13 | 6401200-074 | 0 | 20 | 0 | 14 | 6401200-053 | 1 | 20 | 1 |
| 13 | 6401200-075 | 2 | 19 | 2 | 14 | 6401200-056 | 1 | 20 | 1 |
| 13 | 6401200-076 | 0 | 20 | 0 | 14 | 6401200-059 | 0 | 20 | 0 |
| 13 | 6401200-077 | 2 | 20 | 2 | 14 | 6401200-060 | 0 | 20 | 0 |
| 13 | 6401200-078 | 0 | 20 | 0 | 14 | 6401200-061 | 0 | 20 | 0 |
| 14 | 6400300-017 | 0 | 1 | 0 | 15 | 6400300-017 | 0 | 1 | 0 |
| 14 | 6400500-014 | 0 | 20 | 0 | 15 | 6400500-014 | 0 | 20 | 0 |
| 14 | 6400500-033 | 1 | 20 | 1 | 15 | 6400500-033 | 1 | 20 | 1 |
| 14 | 6400500-051 | 1 | 20 | 1 | 15 | 6400500-051 | 1 | 20 | 1 |
| 14 | 6400500-054 | 3 | 20 | 3 | 15 | 6400500-054 | 3 | 20 | 3 |
| 14 | 6400600-002 | 1 | 20 | 1 | 15 | 6400600-002 | 1 | 20 | 1 |
| 14 | 6400800-039 | 5 | 20 | 3 | 15 | 6400800-039 | 5 | 20 | 3 |
| 14 | 6400900-004 | 0 | 1 | 0 | 15 | 6400900-001 | 0 | 1 | 0 |
| 15 | 6400900-002 | 0 | 1 | 0 | 16 | 6400800-031 | 0 | 20 | 0 |
| 15 | 6400900-029 | 0 | 1 | 0 | 16 | 6400800-032 | 0 | 20 | 0 |
| 15 | 6400900-043 | 0 | 1 | 0 | 16 | 6400800-033 | 2 | 20 | 2 |
| 15 | 6401200-001 | 0 | 1 | 0 | 16 | 6400800-034 | 1 | 20 | 1 |
| 15 | 6401200-002 | 0 | 1 | 0 | 16 | 6400800-036 | 3 | 20 | 1 |
| 15 | 6401200-007 | 0 | 20 | 0 | 16 | 6400800-037 | 3 | 20 | 2 |
| 15 | 6401200-010 | 0 | 19 | 0 | 16 | 6400800-038 | 26 | 20 | 10 |
| 15 | 6401200-018 | 0 | 1 | 0 | 16 | 6400800-040 | 0 | 20 | 0 |
| 15 | 6401200-021 | 0 | 20 | 0 | 16 | 6400800-041 | 0 | 20 | 0 |
| 15 | 6401200-030 | 0 | 18 | 0 | 16 | 6400800-042 | 8 | 20 | 3 |
| 15 | 6401200-031 | 4 | 20 | 2 | 16 | 6400800-043 | 5 | 20 | 4 |
| 15 | 6401200-035 | 0 | 20 | 0 | 16 | 6400800-044 | 1 | 20 | 1 |
| 15 | 6401200-041 | 0 | 20 | 0 | 16 | 6400800-045 | 2 | 20 | 1 |
| 15 | 6401200-045 | 0 | 10 | 0 | 16 | 6400800-046 | 0 | 20 | 0 |
| 15 | 6401200-050 | 0 | 20 | 0 | 16 | 6400800-047 | 1 | 20 | 1 |
| 15 | 6401200-051 | 0 | 20 | 0 | 16 | 6400800-048 | 1 | 20 | 1 |
| 15 | 6401200-053 | 1 | 20 | 1 | 16 | 6400800-049 | 2 | 20 | 2 |
| 15 | 6401200-058 | 0 | 2 | 0 | 16 | 6400800-050 | 2 | 20 | 2 |
| 15 | 6401200-061 | 0 | 20 | 0 | 16 | 6400800-051 | 1 | 20 | 1 |
| 16 | 6400300-017 | 31 | 20 | 13 | 16 | 6400800-052 | 0 | 20 | 0 |
| 16 | 6400500-014 | 0 | 20 | 0 | 16 | 6400800-053 | 0 | 20 | 0 |
| 16 | 6400500-033 | 1 | 20 | 1 | 16 | 6400800-054 | 2 | 20 | 2 |
| 16 | 6400500-054 | 3 | 20 | 3 | 16 | 6400800-055 | 0 | 20 | 0 |
| 16 | 6400500-083 | 0 | 20 | 0 | 16 | 6400800-056 | 2 | 20 | 1 |
| 16 | 6400600-023 | 0 | 20 | 0 | 16 | 6400800-057 | 7 | 20 | 5 |
| 16 | 6400800-005 | 1 | 20 | 1 | 16 | 6400800-059 | 5 | 20 | 3 |
| 16 | 6400800-006 | 2 | 20 | 1 | 16 | 6400800-060 | 1 | 20 | 1 |
| 16 | 6400800-007 | 0 | 20 | 0 | 16 | 6400800-061 | 1 | 20 | 1 |
| 16 | 6400800-008 | 1 | 20 | 1 | 16 | 6400800-062 | 8 | 20 | 4 |
| 16 | 6400800-010 | 1 | 20 | 1 | 16 | 6400800-063 | 1 | 20 | 1 |
| 16 | 6400800-024 | 0 | 20 | 0 | 16 | 6400800-064 | 1 | 20 | 1 |
| 16 | 6400800-025 | 1 | 20 | 1 | 16 | 6400800-065 | 3 | 20 | 3 |
| 16 | 6400800-026 | 4 | 20 | 2 | 16 | 6400800-066 | 0 | 20 | 0 |
| 16 | 6400800-027 | 0 | 20 | 0 | 16 | 6400800-067 | 0 | 20 | 0 |
| 16 | 6400800-028 | 0 | 20 | 0 | 16 | 6400800-068 | 1 | 20 | 1 |
| 16 | 6400800-029 | 1 | 20 | 1 | 16 | 6400800-069 | 0 | 20 | 0 |
| 16 | 6400800-030 | 1 | 20 | 1 | 16 | 6400900-001 | 0 | 1 | 0 |
| 16 | 6400900-002 | 0 | 1 | 0 | 16 | 6400900-040 | 0 | 20 | 0 |
| 16 | 6400900-003 | 2 | 20 | 2 | 16 | 6400900-041 | 1 | 20 | 1 |
| 16 | 6400900-004 | 1 | 20 | 1 | 16 | 6400900-042 | 2 | 20 | 1 |
| 16 | 6400900-005 | 4 | 20 | 3 | 16 | 6400900-043 | 0 | 1 | 0 |
| 16 | 6400900-006 | 3 | 20 | 3 | 16 | 6400900-044 | 8 | 20 | 5 |
| 16 | 6400900-007 | 0 | 20 | 0 | 16 | 6400900-045 | 2 | 20 | 2 |
| 16 | 6400900-008 | 1 | 20 | 1 | 16 | 6400900-046 | 2 | 20 | 2 |
| 16 | 6400900-009 | 4 | 20 | 2 | 16 | 6400900-047 | 3 | 20 | 3 |
| 16 | 6400900-010 | 2 | 20 | 2 | 16 | 6400900-048 | 1 | 20 | 1 |
| 16 | 6400900-011 | 1 | 20 | 1 | 16 | 6400900-049 | 0 | 20 | 0 |
| 16 | 6400900-012 | 4 | 20 | 3 | 16 | 6400900-050 | 2 | 20 | 2 |
| 16 | 6400900-013 | 1 | 20 | 1 | 16 | 6400900-051 | 4 | 20 | 4 |
| 16 | 6400900-014 | 6 | 20 | 3 | 16 | 6400900-052 | 0 | 20 | 0 |
| 16 | 6400900-015 | 1 | 20 | 1 | 16 | 6400900-053 | 0 | 20 | 0 |
| 16 | 6400900-016 | 1 | 20 | 1 | 16 | 6400900-054 | 1 | 20 | 1 |
| 16 | 6400900-017 | 2 | 20 | 1 | 16 | 6400900-055 | 0 | 20 | 0 |
| 16 | 6400900-018 | 5 | 20 | 3 | 16 | 6400900-056 | 0 | 20 | 0 |
| 16 | 6400900-019 | 1 | 20 | 1 | 16 | 6400900-057 | 0 | 20 | 0 |
| 16 | 6400900-020 | 4 | 20 | 4 | 16 | 6400900-058 | 0 | 20 | 0 |
| 16 | 6400900-021 | 0 | 20 | 0 | 16 | 6400900-059 | 0 | 20 | 0 |
| 16 | 6400900-022 | 1 | 20 | 1 | 16 | 6400900-060 | 0 | 20 | 0 |
| 16 | 6400900-023 | 1 | 20 | 1 | 16 | 6400900-061 | 2 | 20 | 2 |
| 16 | 6400900-024 | 2 | 20 | 2 | 16 | 6401200-001 | 0 | 1 | 0 |
| 16 | 6400900-025 | 6 | 20 | 3 | 16 | 6401200-002 | 0 | 1 | 0 |
| 16 | 6400900-028 | 2 | 20 | 2 | 16 | 6401200-003 | 2 | 20 | 1 |
| 16 | 6400900-029 | 0 | 1 | 0 | 16 | 6401200-007 | 0 | 20 | 0 |
| 16 | 6400900-030 | 1 | 20 | 1 | 16 | 6401200-010 | 0 | 19 | 0 |
| 16 | 6400900-031 | 2 | 20 | 2 | 16 | 6401200-014 | 0 | 20 | 0 |
| 16 | 6400900-032 | 1 | 20 | 1 | 16 | 6401200-015 | 2 | 20 | 2 |
| 16 | 6400900-033 | 2 | 20 | 2 | 16 | 6401200-018 | 0 | 1 | 0 |
| 16 | 6400900-034 | 1 | 20 | 1 | 16 | 6401200-021 | 0 | 20 | 0 |
| 16 | 6400900-035 | 3 | 20 | 2 | 16 | 6401200-022 | 0 | 20 | 0 |
| 16 | 6400900-036 | 0 | 20 | 0 | 16 | 6401200-035 | 0 | 20 | 0 |
| 16 | 6400900-037 | 2 | 20 | 1 | 16 | 6401200-041 | 0 | 20 | 0 |
| 16 | 6400900-038 | 0 | 20 | 0 | 16 | 6401200-045 | 15 | 20 | 7 |
| 16 | 6400900-039 | 12 | 20 | 9 | 16 | 6401200-050 | 0 | 20 | 0 |
| 16 | 6401200-051 | 0 | 20 | 0 | 17 | 6400800-051 | 1 | 20 | 1 |
| 16 | 6401200-058 | 0 | 2 | 0 | 17 | 6400800-052 | 0 | 20 | 0 |
| 16 | 6401200-061 | 0 | 20 | 0 | 17 | 6400800-053 | 0 | 20 | 0 |
| 17 | 6400300-017 | 31 | 20 | 13 | 17 | 6400800-054 | 2 | 20 | 2 |
| 17 | 6400500-083 | 0 | 20 | 0 | 17 | 6400800-055 | 0 | 20 | 0 |
| 17 | 6400600-023 | 0 | 20 | 0 | 17 | 6400800-056 | 2 | 20 | 1 |
| 17 | 6400800-005 | 1 | 20 | 1 | 17 | 6400800-057 | 7 | 20 | 5 |
| 17 | 6400800-006 | 2 | 20 | 1 | 17 | 6400800-059 | 5 | 20 | 3 |
| 17 | 6400800-007 | 0 | 20 | 0 | 17 | 6400800-060 | 1 | 20 | 1 |
| 17 | 6400800-008 | 1 | 20 | 1 | 17 | 6400800-061 | 1 | 20 | 1 |
| 17 | 6400800-010 | 1 | 20 | 1 | 17 | 6400800-062 | 8 | 20 | 4 |
| 17 | 6400800-024 | 0 | 20 | 0 | 17 | 6400800-063 | 1 | 20 | 1 |
| 17 | 6400800-025 | 1 | 20 | 1 | 17 | 6400800-064 | 1 | 20 | 1 |
| 17 | 6400800-026 | 4 | 20 | 2 | 17 | 6400800-065 | 3 | 20 | 3 |
| 17 | 6400800-027 | 0 | 20 | 0 | 17 | 6400800-066 | 0 | 20 | 0 |
| 17 | 6400800-028 | 0 | 20 | 0 | 17 | 6400800-067 | 0 | 20 | 0 |
| 17 | 6400800-029 | 1 | 20 | 1 | 17 | 6400800-068 | 1 | 20 | 1 |
| 17 | 6400800-030 | 1 | 20 | 1 | 17 | 6400800-069 | 0 | 20 | 0 |
| 17 | 6400800-031 | 0 | 20 | 0 | 17 | 6400900-001 | 1 | 21 | 1 |
| 17 | 6400800-032 | 0 | 20 | 0 | 17 | 6400900-002 | 2 | 20 | 2 |
| 17 | 6400800-033 | 2 | 20 | 2 | 17 | 6400900-003 | 2 | 20 | 2 |
| 17 | 6400800-034 | 1 | 20 | 1 | 17 | 6400900-004 | 1 | 20 | 1 |
| 17 | 6400800-036 | 3 | 20 | 1 | 17 | 6400900-005 | 4 | 20 | 3 |
| 17 | 6400800-037 | 3 | 20 | 2 | 17 | 6400900-006 | 3 | 20 | 3 |
| 17 | 6400800-038 | 26 | 20 | 10 | 17 | 6400900-007 | 0 | 20 | 0 |
| 17 | 6400800-040 | 0 | 20 | 0 | 17 | 6400900-008 | 1 | 20 | 1 |
| 17 | 6400800-041 | 0 | 20 | 0 | 17 | 6400900-009 | 4 | 20 | 2 |
| 17 | 6400800-042 | 8 | 20 | 3 | 17 | 6400900-010 | 2 | 20 | 2 |
| 17 | 6400800-043 | 5 | 20 | 4 | 17 | 6400900-011 | 1 | 20 | 1 |
| 17 | 6400800-044 | 1 | 20 | 1 | 17 | 6400900-012 | 4 | 20 | 3 |
| 17 | 6400800-045 | 2 | 20 | 1 | 17 | 6400900-013 | 1 | 20 | 1 |
| 17 | 6400800-046 | 0 | 20 | 0 | 17 | 6400900-014 | 6 | 20 | 3 |
| 17 | 6400800-047 | 1 | 20 | 1 | 17 | 6400900-015 | 1 | 20 | 1 |
| 17 | 6400800-048 | 1 | 20 | 1 | 17 | 6400900-016 | 1 | 20 | 1 |
| 17 | 6400800-049 | 2 | 20 | 2 | 17 | 6400900-017 | 2 | 20 | 1 |
| 17 | 6400800-050 | 2 | 20 | 2 | 17 | 6400900-018 | 5 | 20 | 3 |
| 17 | 6400900-019 | 1 | 20 | 1 | 17 | 6400900-057 | 0 | 20 | 0 |
| 17 | 6400900-020 | 4 | 20 | 4 | 17 | 6400900-058 | 0 | 20 | 0 |
| 17 | 6400900-021 | 0 | 20 | 0 | 17 | 6400900-059 | 0 | 20 | 0 |
| 17 | 6400900-022 | 1 | 20 | 1 | 17 | 6400900-060 | 0 | 20 | 0 |
| 17 | 6400900-023 | 1 | 20 | 1 | 17 | 6400900-061 | 2 | 20 | 2 |
| 17 | 6400900-024 | 2 | 20 | 2 | 17 | 6401200-001 | 2 | 19 | 1 |
| 17 | 6400900-025 | 6 | 20 | 3 | 17 | 6401200-002 | 3 | 19 | 1 |
| 17 | 6400900-028 | 2 | 20 | 2 | 17 | 6401200-003 | 2 | 20 | 1 |
| 17 | 6400900-029 | 2 | 20 | 2 | 17 | 6401200-014 | 0 | 20 | 0 |
| 17 | 6400900-030 | 1 | 20 | 1 | 17 | 6401200-015 | 2 | 20 | 2 |
| 17 | 6400900-031 | 2 | 20 | 2 | 17 | 6401200-018 | 5 | 19 | 4 |
| 17 | 6400900-032 | 1 | 20 | 1 | 17 | 6401200-022 | 0 | 20 | 0 |
| 17 | 6400900-033 | 2 | 20 | 2 | 17 | 6401200-045 | 15 | 20 | 7 |
| 17 | 6400900-034 | 1 | 20 | 1 | 17 | 6401200-058 | 6 | 18 | 6 |
| 17 | 6400900-035 | 3 | 20 | 2 | 18 | 6400300-017 | 31 | 20 | 13 |
| 17 | 6400900-036 | 0 | 20 | 0 | 18 | 6400500-083 | 8 | 20 | 6 |
| 17 | 6400900-037 | 2 | 20 | 1 | 18 | 6400600-023 | 0 | 20 | 0 |
| 17 | 6400900-038 | 0 | 20 | 0 | 18 | 6400700-005 | 1 | 20 | 1 |
| 17 | 6400900-039 | 12 | 20 | 9 | 18 | 6400800-001 | 3 | 20 | 3 |
| 17 | 6400900-040 | 0 | 20 | 0 | 18 | 6400800-002 | 0 | 20 | 0 |
| 17 | 6400900-041 | 1 | 20 | 1 | 18 | 6400800-005 | 1 | 20 | 1 |
| 17 | 6400900-042 | 2 | 20 | 1 | 18 | 6400800-006 | 15 | 20 | 6 |
| 17 | 6400900-043 | 0 | 20 | 0 | 18 | 6400800-007 | 2 | 20 | 2 |
| 17 | 6400900-044 | 8 | 20 | 5 | 18 | 6400800-008 | 0 | 20 | 0 |
| 17 | 6400900-045 | 2 | 20 | 2 | 18 | 6400800-010 | 5 | 20 | 4 |
| 17 | 6400900-046 | 2 | 20 | 2 | 18 | 6400800-017 | 3 | 20 | 2 |
| 17 | 6400900-047 | 3 | 20 | 3 | 18 | 6400800-022 | 2 | 20 | 1 |
| 17 | 6400900-048 | 1 | 20 | 1 | 18 | 6400800-024 | 2 | 20 | 1 |
| 17 | 6400900-049 | 0 | 20 | 0 | 18 | 6400800-025 | 0 | 20 | 0 |
| 17 | 6400900-050 | 2 | 20 | 2 | 18 | 6400800-026 | 4 | 20 | 2 |
| 17 | 6400900-051 | 4 | 20 | 4 | 18 | 6400800-027 | 0 | 20 | 0 |
| 17 | 6400900-052 | 0 | 20 | 0 | 18 | 6400800-028 | 1 | 20 | 1 |
| 17 | 6400900-053 | 0 | 20 | 0 | 18 | 6400800-029 | 6 | 20 | 4 |
| 17 | 6400900-054 | 1 | 20 | 1 | 18 | 6400800-030 | 4 | 20 | 4 |
| 17 | 6400900-055 | 0 | 20 | 0 | 18 | 6400800-031 | 0 | 20 | 0 |
| 17 | 6400900-056 | 0 | 20 | 0 | 18 | 6400800-032 | 11 | 20 | 3 |
| 18 | 6400800-033 | 7 | 20 | 5 | 18 | 6400900-002 | 2 | 20 | 2 |
| 18 | 6400800-034 | 0 | 20 | 0 | 18 | 6400900-003 | 2 | 20 | 2 |
| 18 | 6400800-036 | 3 | 20 | 1 | 18 | 6400900-004 | 1 | 20 | 1 |
| 18 | 6400800-037 | 3 | 20 | 2 | 18 | 6400900-005 | 4 | 20 | 3 |
| 18 | 6400800-038 | 26 | 20 | 14 | 18 | 6400900-006 | 3 | 20 | 3 |
| 18 | 6400800-040 | 0 | 20 | 0 | 18 | 6400900-007 | 0 | 20 | 0 |
| 18 | 6400800-041 | 0 | 20 | 0 | 18 | 6400900-008 | 1 | 20 | 1 |
| 18 | 6400800-042 | 8 | 20 | 3 | 18 | 6400900-009 | 4 | 20 | 2 |
| 18 | 6400800-043 | 8 | 20 | 4 | 18 | 6400900-010 | 2 | 20 | 2 |
| 18 | 6400800-044 | 3 | 20 | 2 | 18 | 6400900-011 | 1 | 20 | 1 |
| 18 | 6400800-045 | 6 | 20 | 2 | 18 | 6400900-012 | 4 | 20 | 3 |
| 18 | 6400800-046 | 0 | 20 | 0 | 18 | 6400900-013 | 1 | 20 | 1 |
| 18 | 6400800-047 | 1 | 20 | 1 | 18 | 6400900-014 | 0 | 1 | 0 |
| 18 | 6400800-048 | 9 | 20 | 5 | 18 | 6400900-015 | 1 | 20 | 1 |
| 18 | 6400800-049 | 4 | 20 | 3 | 18 | 6400900-016 | 1 | 20 | 1 |
| 18 | 6400800-050 | 2 | 20 | 2 | 18 | 6400900-017 | 2 | 20 | 1 |
| 18 | 6400800-051 | 3 | 20 | 3 | 18 | 6400900-018 | 5 | 20 | 3 |
| 18 | 6400800-052 | 0 | 20 | 0 | 18 | 6400900-019 | 1 | 20 | 1 |
| 18 | 6400800-053 | 0 | 20 | 0 | 18 | 6400900-020 | 4 | 20 | 4 |
| 18 | 6400800-054 | 6 | 20 | 5 | 18 | 6400900-021 | 0 | 20 | 0 |
| 18 | 6400800-055 | 0 | 20 | 0 | 18 | 6400900-022 | 1 | 20 | 1 |
| 18 | 6400800-056 | 2 | 20 | 1 | 18 | 6400900-023 | 1 | 20 | 1 |
| 18 | 6400800-057 | 18 | 20 | 9 | 18 | 6400900-024 | 2 | 20 | 2 |
| 18 | 6400800-059 | 5 | 20 | 3 | 18 | 6400900-025 | 6 | 20 | 3 |
| 18 | 6400800-060 | 1 | 20 | 1 | 18 | 6400900-028 | 2 | 20 | 2 |
| 18 | 6400800-061 | 1 | 20 | 1 | 18 | 6400900-029 | 2 | 20 | 2 |
| 18 | 6400800-062 | 8 | 20 | 4 | 18 | 6400900-030 | 1 | 20 | 1 |
| 18 | 6400800-063 | 1 | 20 | 1 | 18 | 6400900-031 | 0 | 1 | 0 |
| 18 | 6400800-064 | 1 | 20 | 1 | 18 | 6400900-032 | 1 | 20 | 1 |
| 18 | 6400800-065 | 3 | 20 | 3 | 18 | 6400900-033 | 2 | 20 | 2 |
| 18 | 6400800-066 | 0 | 20 | 0 | 18 | 6400900-034 | 1 | 20 | 1 |
| 18 | 6400800-067 | 0 | 20 | 0 | 18 | 6400900-035 | 3 | 20 | 2 |
| 18 | 6400800-068 | 1 | 20 | 1 | 18 | 6400900-036 | 0 | 20 | 0 |
| 18 | 6400800-069 | 0 | 20 | 0 | 18 | 6400900-037 | 2 | 20 | 1 |
| 18 | 6400900-001 | 0 | 1 | 0 | 18 | 6400900-038 | 0 | 20 | 0 |
| 18 | 6400900-039 | 12 | 20 | 9 | 19 | 6400600-023 | 0 | 20 | 0 |
| 18 | 6400900-040 | 0 | 20 | 0 | 19 | 6400700-005 | 1 | 20 | 1 |
| 18 | 6400900-041 | 1 | 20 | 1 | 19 | 6400700-007 | 0 | 20 | 0 |
| 18 | 6400900-042 | 2 | 20 | 1 | 19 | 6400800-001 | 3 | 20 | 3 |
| 18 | 6400900-043 | 0 | 20 | 0 | 19 | 6400800-002 | 0 | 20 | 0 |
| 18 | 6400900-044 | 8 | 20 | 5 | 19 | 6400800-005 | 1 | 20 | 1 |
| 18 | 6400900-045 | 2 | 20 | 2 | 19 | 6400800-006 | 15 | 20 | 6 |
| 18 | 6400900-046 | 2 | 20 | 2 | 19 | 6400800-007 | 2 | 20 | 2 |
| 18 | 6400900-047 | 3 | 20 | 3 | 19 | 6400800-008 | 0 | 20 | 0 |
| 18 | 6400900-048 | 1 | 20 | 1 | 19 | 6400800-010 | 5 | 20 | 4 |
| 18 | 6400900-049 | 0 | 20 | 0 | 19 | 6400800-017 | 3 | 20 | 2 |
| 18 | 6400900-050 | 2 | 20 | 2 | 19 | 6400800-018 | 2 | 20 | 2 |
| 18 | 6400900-051 | 0 | 1 | 0 | 19 | 6400800-019 | 0 | 20 | 0 |
| 18 | 6400900-052 | 0 | 20 | 0 | 19 | 6400800-020 | 1 | 20 | 1 |
| 18 | 6400900-053 | 0 | 20 | 0 | 19 | 6400800-022 | 2 | 20 | 1 |
| 18 | 6400900-054 | 1 | 20 | 1 | 19 | 6400800-023 | 0 | 20 | 0 |
| 18 | 6400900-055 | 0 | 20 | 0 | 19 | 6400800-024 | 2 | 20 | 1 |
| 18 | 6400900-056 | 0 | 20 | 0 | 19 | 6400800-025 | 0 | 20 | 0 |
| 18 | 6400900-057 | 0 | 20 | 0 | 19 | 6400800-028 | 1 | 20 | 1 |
| 18 | 6400900-058 | 0 | 20 | 0 | 19 | 6400800-029 | 6 | 20 | 4 |
| 18 | 6400900-059 | 0 | 20 | 0 | 19 | 6400800-030 | 4 | 20 | 4 |
| 18 | 6400900-060 | 0 | 20 | 0 | 19 | 6400800-031 | 0 | 20 | 0 |
| 18 | 6400900-061 | 2 | 20 | 2 | 19 | 6400800-032 | 11 | 20 | 3 |
| 18 | 6401200-001 | 2 | 19 | 1 | 19 | 6400800-033 | 7 | 20 | 5 |
| 18 | 6401200-002 | 3 | 19 | 1 | 19 | 6400800-034 | 0 | 20 | 0 |
| 18 | 6401200-003 | 2 | 20 | 1 | 19 | 6400800-035 | 0 | 20 | 0 |
| 18 | 6401200-014 | 0 | 20 | 0 | 19 | 6400800-038 | 26 | 20 | 14 |
| 18 | 6401200-015 | 2 | 20 | 2 | 19 | 6400800-043 | 8 | 20 | 4 |
| 18 | 6401200-018 | 5 | 19 | 4 | 19 | 6400800-044 | 3 | 20 | 2 |
| 18 | 6401200-022 | 0 | 20 | 0 | 19 | 6400800-045 | 6 | 20 | 2 |
| 18 | 6401200-045 | 15 | 20 | 7 | 19 | 6400800-047 | 1 | 20 | 1 |
| 18 | 6401200-058 | 6 | 18 | 6 | 19 | 6400800-048 | 9 | 20 | 5 |
| 19 | 6400300-017 | 4 | 1 | 1 | 19 | 6400800-049 | 4 | 20 | 3 |
| 19 | 6400500-012 | 1 | 20 | 1 | 19 | 6400800-051 | 3 | 20 | 3 |
| 19 | 6400500-083 | 8 | 20 | 6 | 19 | 6400800-052 | 0 | 20 | 0 |
| 19 | 6400600-005 | 0 | 1 | 0 | 19 | 6400800-053 | 0 | 20 | 0 |
| 19 | 6400800-054 | 6 | 20 | 5 | 20 | 6400700-007 | 0 | 20 | 0 |
| 19 | 6400800-057 | 18 | 20 | 9 | 20 | 6400800-001 | 3 | 20 | 3 |
| 19 | 6400900-001 | 1 | 1 | 1 | 20 | 6400800-002 | 0 | 20 | 0 |
| 19 | 6400900-007 | 0 | 1 | 0 | 20 | 6400800-003 | 2 | 20 | 2 |
| 19 | 6400900-009 | 0 | 1 | 0 | 20 | 6400800-004 | 0 | 20 | 0 |
| 19 | 6400900-014 | 0 | 1 | 0 | 20 | 6400800-009 | 0 | 20 | 0 |
| 19 | 6400900-029 | 0 | 1 | 0 | 20 | 6400800-011 | 1 | 20 | 1 |
| 19 | 6400900-031 | 0 | 2 | 0 | 20 | 6400800-012 | 0 | 20 | 0 |
| 19 | 6400900-049 | 0 | 1 | 0 | 20 | 6400800-013 | 0 | 20 | 0 |
| 19 | 6400900-051 | 0 | 1 | 0 | 20 | 6400800-014 | 0 | 20 | 0 |
| 19 | 6401200-035 | 2 | 20 | 1 | 20 | 6400800-015 | 0 | 20 | 0 |
| 20 | 6400300-017 | 4 | 1 | 1 | 20 | 6400800-016 | 1 | 20 | 1 |
| 20 | 6400500-012 | 1 | 20 | 1 | 20 | 6400800-017 | 3 | 20 | 2 |
| 20 | 6400500-013 | 0 | 1 | 0 | 20 | 6400800-018 | 2 | 20 | 2 |
| 20 | 6400500-018 | 0 | 1 | 0 | 20 | 6400800-019 | 0 | 20 | 0 |
| 20 | 6400600-002 | 0 | 20 | 0 | 20 | 6400800-020 | 1 | 20 | 1 |
| 20 | 6400600-003 | 0 | 20 | 0 | 20 | 6400800-021 | 0 | 20 | 0 |
| 20 | 6400600-004 | 1 | 20 | 1 | 20 | 6400800-022 | 2 | 20 | 1 |
| 20 | 6400600-005 | 0 | 1 | 0 | 20 | 6400800-023 | 0 | 20 | 0 |
| 20 | 6400600-006 | 2 | 10 | 2 | 20 | 6400800-035 | 0 | 20 | 0 |
| 20 | 6400600-007 | 0 | 10 | 0 | 20 | 6400800-039 | 2 | 20 | 2 |
| 20 | 6400600-008 | 0 | 20 | 0 | 20 | 6400800-058 | 9 | 20 | 7 |
| 20 | 6400600-009 | 5 | 10 | 1 | 20 | 6400900-001 | 1 | 1 | 1 |
| 20 | 6400600-010 | 1 | 10 | 1 | 20 | 6400900-007 | 0 | 1 | 0 |
| 20 | 6400600-011 | 1 | 20 | 1 | 20 | 6400900-009 | 0 | 1 | 0 |
| 20 | 6400600-012 | 0 | 20 | 0 | 20 | 6400900-029 | 0 | 1 | 0 |
| 20 | 6400600-018 | 0 | 20 | 0 | 20 | 6400900-031 | 0 | 2 | 0 |
| 20 | 6400600-019 | 4 | 20 | 1 | 20 | 6400900-049 | 0 | 1 | 0 |
| 20 | 6400600-020 | 1 | 20 | 1 | 20 | 6400900-051 | 0 | 1 | 0 |
| 20 | 6400600-021 | 0 | 20 | 0 | 20 | 6401200-035 | 2 | 20 | 1 |
| 20 | 6400600-022 | 0 | 20 | 0 | 21 | 6400300-017 | 4 | 1 | 1 |
| 20 | 6400600-024 | 0 | 20 | 0 | 21 | 6400300-019 | 36 | 20 | 10 |
| 20 | 6400600-025 | 0 | 20 | 0 | 21 | 6400500-001 | 0 | 4 | 0 |
| 20 | 6400600-027 | 1 | 20 | 1 | 21 | 6400500-012 | 1 | 20 | 1 |
| 20 | 6400600-028 | 0 | 20 | 0 | 21 | 6400500-013 | 0 | 1 | 0 |
| 20 | 6400600-031 | 1 | 20 | 1 | 21 | 6400500-015 | 2 | 20 | 2 |
| 20 | 6400700-005 | 1 | 20 | 1 | 21 | 6400500-018 | 0 | 1 | 0 |
| 21 | 6400500-021 | 0 | 20 | 0 | 21 | 6400600-030 | 3 | 20 | 2 |
| 21 | 6400500-023 | 0 | 20 | 0 | 21 | 6400600-031 | 1 | 20 | 1 |
| 21 | 6400500-024 | 0 | 20 | 0 | 21 | 6400600-032 | 4 | 20 | 4 |
| 21 | 6400500-025 | 2 | 20 | 2 | 21 | 6400700-007 | 0 | 20 | 0 |
| 21 | 6400500-030 | 3 | 20 | 2 | 21 | 6400800-003 | 2 | 20 | 2 |
| 21 | 6400500-052 | 1 | 20 | 1 | 21 | 6400800-004 | 0 | 20 | 0 |
| 21 | 6400500-055 | 0 | 20 | 0 | 21 | 6400800-009 | 0 | 20 | 0 |
| 21 | 6400500-068 | 2 | 20 | 2 | 21 | 6400800-011 | 1 | 20 | 1 |
| 21 | 6400500-070 | 1 | 20 | 1 | 21 | 6400800-012 | 0 | 20 | 0 |
| 21 | 6400500-072 | 3 | 20 | 2 | 21 | 6400800-013 | 0 | 20 | 0 |
| 21 | 6400500-073 | 11 | 20 | 4 | 21 | 6400800-014 | 0 | 20 | 0 |
| 21 | 6400600-002 | 0 | 20 | 0 | 21 | 6400800-015 | 0 | 20 | 0 |
| 21 | 6400600-003 | 0 | 20 | 0 | 21 | 6400800-016 | 1 | 20 | 1 |
| 21 | 6400600-004 | 1 | 20 | 1 | 21 | 6400800-018 | 2 | 20 | 2 |
| 21 | 6400600-005 | 2 | 19 | 1 | 21 | 6400800-019 | 0 | 20 | 0 |
| 21 | 6400600-006 | 2 | 10 | 2 | 21 | 6400800-020 | 0 | 20 | 0 |
| 21 | 6400600-007 | 0 | 10 | 0 | 21 | 6400800-021 | 0 | 20 | 0 |
| 21 | 6400600-008 | 0 | 20 | 0 | 21 | 6400800-023 | 0 | 20 | 0 |
| 21 | 6400600-009 | 5 | 10 | 1 | 21 | 6400800-035 | 0 | 20 | 0 |
| 21 | 6400600-010 | 1 | 10 | 1 | 21 | 6400800-039 | 2 | 20 | 2 |
| 21 | 6400600-011 | 1 | 20 | 1 | 21 | 6400800-058 | 9 | 20 | 7 |
| 21 | 6400600-012 | 0 | 20 | 0 | 21 | 6400900-001 | 1 | 1 | 1 |
| 21 | 6400600-013 | 7 | 20 | 5 | 21 | 6400900-009 | 0 | 1 | 0 |
| 21 | 6400600-014 | 0 | 20 | 0 | 21 | 6400900-013 | 0 | 1 | 0 |
| 21 | 6400600-015 | 2 | 20 | 2 | 21 | 6400900-029 | 0 | 1 | 0 |
| 21 | 6400600-018 | 0 | 20 | 0 | 21 | 6401200-035 | 2 | 20 | 1 |
| 21 | 6400600-019 | 4 | 20 | 1 | 22 | 6400100-006 | 1 | 20 | 1 |
| 21 | 6400600-020 | 1 | 20 | 1 | 22 | 6400300-019 | 36 | 20 | 10 |
| 21 | 6400600-021 | 0 | 20 | 0 | 22 | 6400500-001 | 0 | 4 | 0 |
| 21 | 6400600-022 | 0 | 20 | 0 | 22 | 6400500-005 | 0 | 20 | 0 |
| 21 | 6400600-024 | 0 | 20 | 0 | 22 | 6400500-013 | 0 | 19 | 0 |
| 21 | 6400600-025 | 0 | 20 | 0 | 22 | 6400500-015 | 2 | 20 | 2 |
| 21 | 6400600-026 | 2 | 20 | 2 | 22 | 6400500-016 | 0 | 20 | 0 |
| 21 | 6400600-027 | 1 | 20 | 1 | 22 | 6400500-017 | 2 | 20 | 2 |
| 21 | 6400600-028 | 0 | 20 | 0 | 22 | 6400500-018 | 3 | 19 | 3 |
| 21 | 6400600-029 | 0 | 20 | 0 | 22 | 6400500-019 | 2 | 20 | 2 |
| 22 | 6400500-021 | 0 | 20 | 0 | 22 | 6400600-025 | 0 | 20 | 0 |
| 22 | 6400500-023 | 0 | 20 | 0 | 22 | 6400600-026 | 2 | 20 | 2 |
| 22 | 6400500-024 | 0 | 20 | 0 | 22 | 6400600-027 | 1 | 20 | 1 |
| 22 | 6400500-025 | 2 | 20 | 2 | 22 | 6400600-028 | 0 | 20 | 0 |
| 22 | 6400500-027 | 0 | 20 | 0 | 22 | 6400600-029 | 0 | 20 | 0 |
| 22 | 6400500-029 | 3 | 20 | 3 | 22 | 6400600-030 | 3 | 20 | 2 |
| 22 | 6400500-030 | 3 | 20 | 2 | 22 | 6400600-031 | 1 | 20 | 1 |
| 22 | 6400500-046 | 0 | 20 | 0 | 22 | 6400600-032 | 4 | 20 | 4 |
| 22 | 6400500-052 | 1 | 20 | 1 | 22 | 6400800-003 | 2 | 20 | 2 |
| 22 | 6400500-053 | 0 | 20 | 0 | 22 | 6400800-004 | 0 | 20 | 0 |
| 22 | 6400500-055 | 0 | 20 | 0 | 22 | 6400800-009 | 0 | 20 | 0 |
| 22 | 6400500-068 | 2 | 20 | 2 | 22 | 6400800-011 | 1 | 20 | 1 |
| 22 | 6400500-070 | 1 | 20 | 1 | 22 | 6400800-012 | 0 | 20 | 0 |
| 22 | 6400500-072 | 3 | 20 | 2 | 22 | 6400800-013 | 0 | 20 | 0 |
| 22 | 6400500-073 | 11 | 20 | 4 | 22 | 6400800-014 | 0 | 20 | 0 |
| 22 | 6400600-001 | 2 | 20 | 1 | 22 | 6400800-015 | 0 | 20 | 0 |
| 22 | 6400600-002 | 0 | 20 | 0 | 22 | 6400800-016 | 1 | 20 | 1 |
| 22 | 6400600-003 | 0 | 20 | 0 | 22 | 6400800-020 | 0 | 20 | 0 |
| 22 | 6400600-004 | 1 | 20 | 1 | 22 | 6400800-021 | 0 | 20 | 0 |
| 22 | 6400600-005 | 2 | 19 | 1 | 22 | 6400800-039 | 2 | 20 | 2 |
| 22 | 6400600-006 | 2 | 10 | 2 | 22 | 6400800-044 | 1 | 20 | 1 |
| 22 | 6400600-007 | 0 | 10 | 0 | 22 | 6400800-058 | 9 | 20 | 7 |
| 22 | 6400600-008 | 0 | 20 | 0 | 22 | 6400900-009 | 0 | 1 | 0 |
| 22 | 6400600-009 | 5 | 10 | 1 | 22 | 6400900-013 | 0 | 1 | 0 |
| 22 | 6400600-010 | 1 | 10 | 1 | 22 | 6400900-014 | 0 | 2 | 0 |
| 22 | 6400600-011 | 1 | 20 | 1 | 22 | 6401200-044 | 11 | 20 | 9 |
| 22 | 6400600-012 | 0 | 20 | 0 | 23 | 6400100-006 | 1 | 20 | 1 |
| 22 | 6400600-013 | 7 | 20 | 5 | 23 | 6400300-019 | 36 | 20 | 10 |
| 22 | 6400600-014 | 0 | 20 | 0 | 23 | 6400500-001 | 0 | 16 | 0 |
| 22 | 6400600-015 | 2 | 20 | 2 | 23 | 6400500-002 | 0 | 1 | 0 |
| 22 | 6400600-018 | 0 | 20 | 0 | 23 | 6400500-005 | 0 | 20 | 0 |
| 22 | 6400600-019 | 4 | 20 | 1 | 23 | 6400500-013 | 0 | 19 | 0 |
| 22 | 6400600-020 | 1 | 20 | 1 | 23 | 6400500-015 | 2 | 20 | 2 |
| 22 | 6400600-021 | 0 | 20 | 0 | 23 | 6400500-016 | 0 | 20 | 0 |
| 22 | 6400600-022 | 0 | 20 | 0 | 23 | 6400500-017 | 2 | 20 | 2 |
| 22 | 6400600-024 | 0 | 20 | 0 | 23 | 6400500-018 | 3 | 19 | 3 |
| 23 | 6400500-019 | 2 | 20 | 2 | 24 | 6400500-004 | 3 | 20 | 2 |
| 23 | 6400500-021 | 0 | 20 | 0 | 24 | 6400500-005 | 0 | 20 | 0 |
| 23 | 6400500-023 | 0 | 20 | 0 | 24 | 6400500-006 | 0 | 20 | 0 |
| 23 | 6400500-024 | 0 | 20 | 0 | 24 | 6400500-007 | 2 | 20 | 1 |
| 23 | 6400500-025 | 2 | 20 | 2 | 24 | 6400500-009 | 0 | 20 | 0 |
| 23 | 6400500-026 | 0 | 1 | 0 | 24 | 6400500-010 | 0 | 20 | 0 |
| 23 | 6400500-027 | 0 | 20 | 0 | 24 | 6400500-016 | 0 | 20 | 0 |
| 23 | 6400500-029 | 3 | 20 | 3 | 24 | 6400500-017 | 2 | 20 | 2 |
| 23 | 6400500-030 | 3 | 20 | 2 | 24 | 6400500-019 | 2 | 20 | 2 |
| 23 | 6400500-046 | 0 | 20 | 0 | 24 | 6400500-022 | 0 | 20 | 0 |
| 23 | 6400500-052 | 1 | 20 | 1 | 24 | 6400500-026 | 0 | 1 | 0 |
| 23 | 6400500-053 | 0 | 20 | 0 | 24 | 6400500-027 | 0 | 20 | 0 |
| 23 | 6400500-055 | 0 | 20 | 0 | 24 | 6400500-029 | 3 | 20 | 3 |
| 23 | 6400500-068 | 2 | 20 | 2 | 24 | 6401200-044 | 11 | 20 | 9 |
| 23 | 6400500-070 | 1 | 20 | 1 | 24 | 6401200-045 | 8 | 1 | 1 |
| 23 | 6400500-072 | 3 | 20 | 2 | 24 | 6401200-071 | 0 | 20 | 0 |
| 23 | 6400500-073 | 11 | 20 | 4 | 25 | 6400500-002 | 0 | 19 | 0 |
| 23 | 6400600-001 | 2 | 20 | 1 | 25 | 6400500-004 | 3 | 20 | 2 |
| 23 | 6400600-013 | 7 | 20 | 5 | 25 | 6400500-006 | 0 | 20 | 0 |
| 23 | 6400600-014 | 0 | 20 | 0 | 25 | 6400500-007 | 2 | 20 | 1 |
| 23 | 6400600-015 | 2 | 20 | 2 | 25 | 6400500-009 | 0 | 20 | 0 |
| 23 | 6400600-026 | 2 | 20 | 2 | 25 | 6400500-010 | 0 | 20 | 0 |
| 23 | 6400600-029 | 0 | 20 | 0 | 25 | 6400500-012 | 1 | 20 | 1 |
| 23 | 6400600-030 | 3 | 20 | 2 | 25 | 6400500-014 | 0 | 20 | 0 |
| 23 | 6400600-032 | 4 | 20 | 4 | 25 | 6400500-020 | 6 | 20 | 2 |
| 23 | 6400800-044 | 1 | 20 | 1 | 25 | 6400500-022 | 0 | 20 | 0 |
| 23 | 6400900-006 | 0 | 1 | 0 | 25 | 6400500-026 | 0 | 19 | 0 |
| 23 | 6400900-013 | 0 | 1 | 0 | 25 | 6400500-028 | 2 | 20 | 2 |
| 23 | 6400900-014 | 0 | 2 | 0 | 25 | 6400500-031 | 2 | 20 | 2 |
| 23 | 6400900-017 | 0 | 1 | 0 | 25 | 6400500-032 | 0 | 20 | 0 |
| 23 | 6401200-044 | 11 | 20 | 9 | 25 | 6400500-033 | 1 | 20 | 1 |
| 23 | 6401200-045 | 8 | 1 | 1 | 25 | 6400500-034 | 2 | 20 | 2 |
| 23 | 6401200-071 | 0 | 20 | 0 | 25 | 6400500-036 | 1 | 20 | 1 |
| 24 | 6400100-006 | 1 | 20 | 1 | 25 | 6400500-037 | 1 | 20 | 1 |
| 24 | 6400500-001 | 0 | 16 | 0 | 25 | 6400500-038 | 0 | 20 | 0 |
| 24 | 6400500-002 | 0 | 1 | 0 | 25 | 6400500-039 | 1 | 20 | 1 |
| 24 | 6400500-031 | 2 | 20 | 2 | 25 | 6400500-040 | 6 | 20 | 6 |
| 24 | 6400500-032 | 0 | 20 | 0 | 25 | 6400500-041 | 0 | 20 | 0 |
| 24 | 6400500-034 | 2 | 20 | 2 | 25 | 6400500-042 | 1 | 20 | 1 |
| 24 | 6400500-036 | 1 | 20 | 1 | 25 | 6400500-043 | 6 | 20 | 4 |
| 24 | 6400500-038 | 0 | 20 | 0 | 25 | 6400500-044 | 1 | 20 | 1 |
| 24 | 6400500-039 | 1 | 20 | 1 | 25 | 6400500-045 | 2 | 20 | 2 |
| 24 | 6400500-040 | 6 | 20 | 6 | 25 | 6400500-047 | 12 | 20 | 4 |
| 24 | 6400500-041 | 0 | 20 | 0 | 25 | 6400500-048 | 1 | 20 | 1 |
| 24 | 6400500-044 | 1 | 20 | 1 | 25 | 6400500-049 | 4 | 20 | 3 |
| 24 | 6400500-045 | 2 | 20 | 2 | 25 | 6400500-050 | 1 | 20 | 1 |
| 24 | 6400500-046 | 0 | 20 | 0 | 25 | 6400500-051 | 0 | 20 | 0 |
| 24 | 6400500-047 | 12 | 20 | 4 | 25 | 6400500-054 | 0 | 20 | 0 |
| 24 | 6400500-048 | 1 | 20 | 1 | 25 | 6400500-056 | 6 | 20 | 6 |
| 24 | 6400500-049 | 4 | 20 | 3 | 25 | 6400500-057 | 2 | 20 | 2 |
| 24 | 6400500-050 | 1 | 20 | 1 | 25 | 6400500-058 | 4 | 20 | 3 |
| 24 | 6400500-051 | 0 | 20 | 0 | 25 | 6400500-059 | 1 | 20 | 1 |
| 24 | 6400500-053 | 0 | 20 | 0 | 25 | 6400500-060 | 0 | 20 | 0 |
| 24 | 6400500-054 | 0 | 20 | 0 | 25 | 6400500-061 | 0 | 20 | 0 |
| 24 | 6400500-059 | 1 | 20 | 1 | 25 | 6400500-062 | 4 | 20 | 3 |
| 24 | 6400500-060 | 0 | 20 | 0 | 25 | 6400500-063 | 0 | 20 | 0 |
| 24 | 6400500-061 | 0 | 20 | 0 | 25 | 6400500-064 | 1 | 20 | 1 |
| 24 | 6400500-062 | 4 | 20 | 3 | 25 | 6400500-065 | 1 | 20 | 1 |
| 24 | 6400500-063 | 0 | 20 | 0 | 25 | 6400500-066 | 4 | 20 | 4 |
| 24 | 6400500-064 | 1 | 20 | 1 | 25 | 6400500-067 | 0 | 1 | 0 |
| 24 | 6400500-067 | 0 | 1 | 0 | 25 | 6400500-074 | 10 | 20 | 3 |
| 24 | 6400500-088 | 0 | 1 | 0 | 25 | 6400500-075 | 1 | 20 | 1 |
| 24 | 6400600-001 | 2 | 20 | 1 | 25 | 6400500-076 | 3 | 20 | 3 |
| 24 | 6400700-005 | 2 | 20 | 2 | 25 | 6400500-077 | 9 | 20 | 4 |
| 24 | 6400700-011 | 8 | 20 | 3 | 25 | 6400500-078 | 7 | 20 | 3 |
| 24 | 6400800-044 | 1 | 20 | 1 | 25 | 6400500-079 | 14 | 20 | 11 |
| 24 | 6400800-051 | 1 | 20 | 1 | 25 | 6400500-085 | 3 | 20 | 2 |
| 24 | 6400800-056 | 3 | 20 | 2 | 25 | 6400500-088 | 0 | 1 | 0 |
| 24 | 6400900-006 | 0 | 1 | 0 | 25 | 6400600-006 | 14 | 10 | 5 |
| 24 | 6400900-013 | 0 | 1 | 0 | 25 | 6400700-005 | 2 | 20 | 2 |
| 24 | 6400900-014 | 0 | 2 | 0 | 25 | 6400700-011 | 8 | 20 | 3 |
| 24 | 6400900-017 | 0 | 1 | 0 | 25 | 6400800-051 | 1 | 20 | 1 |
| 25 | 6400800-056 | 3 | 20 | 2 | 26 | 6400500-039 | 1 | 20 | 1 |
| 25 | 6400800-061 | 5 | 20 | 3 | 26 | 6400500-040 | 6 | 20 | 6 |
| 25 | 6400900-002 | 0 | 1 | 0 | 26 | 6400500-041 | 0 | 20 | 0 |
| 25 | 6400900-006 | 0 | 1 | 0 | 26 | 6400500-042 | 1 | 20 | 1 |
| 25 | 6400900-013 | 0 | 1 | 0 | 26 | 6400500-043 | 6 | 20 | 4 |
| 25 | 6400900-015 | 0 | 1 | 0 | 26 | 6400500-044 | 1 | 20 | 1 |
| 25 | 6400900-017 | 0 | 1 | 0 | 26 | 6400500-045 | 2 | 20 | 2 |
| 25 | 6400900-048 | 0 | 1 | 0 | 26 | 6400500-047 | 12 | 20 | 4 |
| 25 | 6400900-049 | 0 | 1 | 0 | 26 | 6400500-048 | 1 | 20 | 1 |
| 25 | 6401200-045 | 8 | 1 | 1 | 26 | 6400500-049 | 4 | 20 | 3 |
| 25 | 6401200-071 | 0 | 20 | 0 | 26 | 6400500-050 | 1 | 20 | 1 |
| 26 | 6400200-013 | 0 | 1 | 0 | 26 | 6400500-051 | 0 | 20 | 0 |
| 26 | 6400200-017 | 0 | 20 | 0 | 26 | 6400500-054 | 0 | 20 | 0 |
| 26 | 6400200-018 | 5 | 20 | 5 | 26 | 6400500-056 | 6 | 20 | 6 |
| 26 | 6400200-020 | 3 | 20 | 3 | 26 | 6400500-057 | 2 | 20 | 2 |
| 26 | 6400500-002 | 0 | 19 | 0 | 26 | 6400500-058 | 4 | 20 | 3 |
| 26 | 6400500-004 | 3 | 20 | 2 | 26 | 6400500-059 | 1 | 20 | 1 |
| 26 | 6400500-006 | 0 | 20 | 0 | 26 | 6400500-060 | 0 | 20 | 0 |
| 26 | 6400500-007 | 2 | 20 | 1 | 26 | 6400500-061 | 0 | 20 | 0 |
| 26 | 6400500-009 | 0 | 20 | 0 | 26 | 6400500-062 | 4 | 20 | 3 |
| 26 | 6400500-010 | 0 | 20 | 0 | 26 | 6400500-063 | 0 | 20 | 0 |
| 26 | 6400500-012 | 1 | 20 | 1 | 26 | 6400500-064 | 1 | 20 | 1 |
| 26 | 6400500-014 | 0 | 20 | 0 | 26 | 6400500-065 | 1 | 20 | 1 |
| 26 | 6400500-020 | 6 | 20 | 2 | 26 | 6400500-066 | 4 | 20 | 4 |
| 26 | 6400500-022 | 0 | 20 | 0 | 26 | 6400500-067 | 6 | 19 | 3 |
| 26 | 6400500-026 | 0 | 19 | 0 | 26 | 6400500-074 | 10 | 20 | 3 |
| 26 | 6400500-027 | 1 | 20 | 1 | 26 | 6400500-075 | 1 | 20 | 1 |
| 26 | 6400500-028 | 2 | 20 | 2 | 26 | 6400500-076 | 3 | 20 | 3 |
| 26 | 6400500-031 | 2 | 20 | 2 | 26 | 6400500-077 | 9 | 20 | 4 |
| 26 | 6400500-032 | 0 | 20 | 0 | 26 | 6400500-078 | 7 | 20 | 3 |
| 26 | 6400500-033 | 1 | 20 | 1 | 26 | 6400500-079 | 14 | 20 | 11 |
| 26 | 6400500-034 | 2 | 20 | 2 | 26 | 6400500-081 | 1 | 20 | 1 |
| 26 | 6400500-036 | 1 | 20 | 1 | 26 | 6400500-082 | 0 | 10 | 0 |
| 26 | 6400500-037 | 1 | 20 | 1 | 26 | 6400500-083 | 5 | 20 | 4 |
| 26 | 6400500-038 | 0 | 20 | 0 | 26 | 6400500-084 | 1 | 20 | 1 |
| 26 | 6400500-085 | 3 | 20 | 2 | 27 | 6400500-012 | 1 | 20 | 1 |
| 26 | 6400500-086 | 5 | 20 | 3 | 27 | 6400500-014 | 0 | 20 | 0 |
| 26 | 6400500-087 | 3 | 20 | 2 | 27 | 6400500-020 | 6 | 20 | 2 |
| 26 | 6400500-088 | 0 | 19 | 0 | 27 | 6400500-027 | 1 | 20 | 1 |
| 26 | 6400600-006 | 14 | 10 | 5 | 27 | 6400500-028 | 2 | 20 | 2 |
| 26 | 6400700-005 | 2 | 20 | 2 | 27 | 6400500-033 | 1 | 20 | 1 |
| 26 | 6400700-011 | 8 | 20 | 3 | 27 | 6400500-037 | 1 | 20 | 1 |
| 26 | 6400800-051 | 1 | 20 | 1 | 27 | 6400500-042 | 1 | 20 | 1 |
| 26 | 6400800-056 | 3 | 20 | 2 | 27 | 6400500-043 | 6 | 20 | 4 |
| 26 | 6400800-061 | 5 | 20 | 3 | 27 | 6400500-056 | 6 | 20 | 6 |
| 26 | 6400900-001 | 0 | 1 | 0 | 27 | 6400500-057 | 2 | 20 | 2 |
| 26 | 6400900-002 | 0 | 1 | 0 | 27 | 6400500-058 | 4 | 20 | 3 |
| 26 | 6400900-013 | 0 | 1 | 0 | 27 | 6400500-065 | 1 | 20 | 1 |
| 26 | 6400900-015 | 0 | 1 | 0 | 27 | 6400500-066 | 4 | 20 | 4 |
| 26 | 6400900-044 | 0 | 1 | 0 | 27 | 6400500-067 | 6 | 19 | 3 |
| 26 | 6400900-048 | 0 | 1 | 0 | 27 | 6400500-074 | 10 | 20 | 3 |
| 26 | 6400900-049 | 0 | 1 | 0 | 27 | 6400500-075 | 1 | 20 | 1 |
| 27 | 6400200-001 | 1 | 20 | 1 | 27 | 6400500-076 | 3 | 20 | 3 |
| 27 | 6400200-002 | 35 | 20 | 12 | 27 | 6400500-077 | 9 | 20 | 4 |
| 27 | 6400200-003 | 0 | 20 | 0 | 27 | 6400500-078 | 7 | 20 | 3 |
| 27 | 6400200-004 | 1 | 20 | 1 | 27 | 6400500-079 | 14 | 20 | 11 |
| 27 | 6400200-005 | 3 | 20 | 3 | 27 | 6400500-081 | 1 | 20 | 1 |
| 27 | 6400200-006 | 2 | 20 | 2 | 27 | 6400500-082 | 0 | 10 | 0 |
| 27 | 6400200-007 | 7 | 20 | 4 | 27 | 6400500-083 | 5 | 20 | 4 |
| 27 | 6400200-013 | 0 | 1 | 0 | 27 | 6400500-084 | 1 | 20 | 1 |
| 27 | 6400200-015 | 2 | 20 | 1 | 27 | 6400500-085 | 3 | 20 | 2 |
| 27 | 6400200-016 | 2 | 20 | 1 | 27 | 6400500-086 | 5 | 20 | 3 |
| 27 | 6400200-017 | 0 | 20 | 0 | 27 | 6400500-087 | 3 | 20 | 2 |
| 27 | 6400200-018 | 5 | 20 | 5 | 27 | 6400500-088 | 0 | 19 | 0 |
| 27 | 6400200-019 | 4 | 20 | 4 | 27 | 6400600-006 | 14 | 10 | 5 |
| 27 | 6400200-020 | 3 | 20 | 3 | 27 | 6400800-015 | 3 | 20 | 2 |
| 27 | 6400200-022 | 0 | 20 | 0 | 27 | 6400800-061 | 5 | 20 | 3 |
| 27 | 6400200-023 | 1 | 20 | 1 | 27 | 6400900-001 | 0 | 1 | 0 |
| 27 | 6400200-027 | 2 | 20 | 1 | 27 | 6400900-002 | 0 | 1 | 0 |
| 27 | 6400200-028 | 0 | 20 | 0 | 27 | 6400900-003 | 0 | 1 | 0 |
| 27 | 6400200-030 | 0 | 1 | 0 | 27 | 6400900-012 | 0 | 1 | 0 |
| 27 | 6400900-013 | 0 | 1 | 0 | 28 | 6400200-026 | 1 | 20 | 1 |
| 27 | 6400900-015 | 0 | 1 | 0 | 28 | 6400200-027 | 2 | 20 | 1 |
| 27 | 6400900-023 | 0 | 1 | 0 | 28 | 6400200-028 | 0 | 20 | 0 |
| 27 | 6400900-041 | 0 | 1 | 0 | 28 | 6400200-029 | 0 | 20 | 0 |
| 27 | 6400900-044 | 0 | 1 | 0 | 28 | 6400200-030 | 0 | 1 | 0 |
| 27 | 6400900-048 | 0 | 1 | 0 | 28 | 6400200-031 | 0 | 20 | 0 |
| 27 | 6400900-049 | 0 | 1 | 0 | 28 | 6400200-032 | 1 | 20 | 1 |
| 27 | 6400900-050 | 0 | 1 | 0 | 28 | 6400200-033 | 2 | 20 | 1 |
| 27 | 6401200-024 | 2 | 3 | 1 | 28 | 6400200-034 | 0 | 20 | 0 |
| 27 | 6401200-037 | 1 | 20 | 1 | 28 | 6400200-035 | 2 | 20 | 2 |
| 27 | 6401200-045 | 5 | 1 | 1 | 28 | 6400200-037 | 2 | 20 | 1 |
| 27 | 6401200-056 | 0 | 1 | 0 | 28 | 6400500-002 | 0 | 20 | 0 |
| 28 | 6400200-001 | 1 | 20 | 1 | 28 | 6400500-027 | 1 | 20 | 1 |
| 28 | 6400200-002 | 35 | 20 | 12 | 28 | 6400500-081 | 1 | 20 | 1 |
| 28 | 6400200-003 | 0 | 20 | 0 | 28 | 6400500-082 | 0 | 10 | 0 |
| 28 | 6400200-004 | 1 | 20 | 1 | 28 | 6400500-083 | 5 | 20 | 4 |
| 28 | 6400200-005 | 3 | 20 | 3 | 28 | 6400500-084 | 1 | 20 | 1 |
| 28 | 6400200-006 | 2 | 20 | 2 | 28 | 6400500-086 | 5 | 20 | 3 |
| 28 | 6400200-007 | 7 | 20 | 4 | 28 | 6400500-087 | 3 | 20 | 2 |
| 28 | 6400200-008 | 0 | 20 | 0 | 28 | 6400800-015 | 3 | 20 | 2 |
| 28 | 6400200-009 | 0 | 20 | 0 | 28 | 6400800-019 | 1 | 20 | 1 |
| 28 | 6400200-010 | 1 | 20 | 1 | 28 | 6400800-039 | 0 | 20 | 0 |
| 28 | 6400200-012 | 3 | 20 | 3 | 28 | 6400900-001 | 0 | 1 | 0 |
| 28 | 6400200-013 | 3 | 19 | 3 | 28 | 6400900-003 | 0 | 1 | 0 |
| 28 | 6400200-014 | 3 | 20 | 2 | 28 | 6400900-012 | 0 | 1 | 0 |
| 28 | 6400200-015 | 2 | 20 | 1 | 28 | 6400900-013 | 0 | 1 | 0 |
| 28 | 6400200-016 | 2 | 20 | 1 | 28 | 6400900-023 | 0 | 1 | 0 |
| 28 | 6400200-017 | 0 | 20 | 0 | 28 | 6400900-041 | 0 | 1 | 0 |
| 28 | 6400200-018 | 5 | 20 | 5 | 28 | 6400900-044 | 0 | 1 | 0 |
| 28 | 6400200-019 | 4 | 20 | 4 | 28 | 6400900-048 | 0 | 2 | 0 |
| 28 | 6400200-020 | 3 | 20 | 3 | 28 | 6400900-049 | 0 | 1 | 0 |
| 28 | 6400200-021 | 8 | 20 | 4 | 28 | 6400900-050 | 0 | 1 | 0 |
| 28 | 6400200-022 | 0 | 20 | 0 | 28 | 6400900-057 | 0 | 1 | 0 |
| 28 | 6400200-023 | 1 | 20 | 1 | 28 | 6401200-011 | 4 | 20 | 4 |
| 28 | 6400200-024 | 4 | 20 | 3 | 28 | 6401200-012 | 0 | 20 | 0 |
| 28 | 6400200-025 | 2 | 20 | 2 | 28 | 6401200-024 | 2 | 3 | 1 |
| 28 | 6401200-037 | 1 | 20 | 1 | 29 | 6400200-033 | 2 | 20 | 1 |
| 28 | 6401200-040 | 0 | 20 | 0 | 29 | 6400200-034 | 0 | 20 | 0 |
| 28 | 6401200-045 | 5 | 1 | 1 | 29 | 6400200-035 | 2 | 20 | 2 |
| 28 | 6401200-047 | 0 | 2 | 0 | 29 | 6400200-036 | 1 | 20 | 1 |
| 28 | 6401200-056 | 0 | 1 | 0 | 29 | 6400200-037 | 2 | 20 | 1 |
| 28 | 6401200-058 | 1 | 20 | 1 | 29 | 6400200-038 | 12 | 20 | 12 |
| 28 | 6401200-060 | 0 | 20 | 0 | 29 | 6400300-017 | 57 | 30 | 19 |
| 29 | 6400100-006 | 2 | 20 | 2 | 29 | 6400500-002 | 0 | 20 | 0 |
| 29 | 6400200-001 | 1 | 20 | 1 | 29 | 6400500-087 | 0 | 20 | 0 |
| 29 | 6400200-002 | 35 | 20 | 12 | 29 | 6400800-007 | 0 | 20 | 0 |
| 29 | 6400200-003 | 0 | 20 | 0 | 29 | 6400800-015 | 3 | 20 | 2 |
| 29 | 6400200-004 | 1 | 20 | 1 | 29 | 6400800-017 | 1 | 20 | 1 |
| 29 | 6400200-005 | 3 | 20 | 3 | 29 | 6400800-019 | 1 | 20 | 1 |
| 29 | 6400200-006 | 2 | 20 | 2 | 29 | 6400800-035 | 0 | 20 | 0 |
| 29 | 6400200-007 | 7 | 20 | 4 | 29 | 6400800-039 | 0 | 20 | 0 |
| 29 | 6400200-008 | 0 | 20 | 0 | 29 | 6400800-045 | 0 | 20 | 0 |
| 29 | 6400200-009 | 0 | 20 | 0 | 29 | 6400900-001 | 0 | 20 | 0 |
| 29 | 6400200-010 | 1 | 20 | 1 | 29 | 6400900-002 | 0 | 20 | 0 |
| 29 | 6400200-011 | 2 | 20 | 2 | 29 | 6400900-003 | 0 | 1 | 0 |
| 29 | 6400200-012 | 3 | 20 | 3 | 29 | 6400900-004 | 0 | 20 | 0 |
| 29 | 6400200-013 | 3 | 19 | 3 | 29 | 6400900-005 | 2 | 20 | 2 |
| 29 | 6400200-014 | 3 | 20 | 2 | 29 | 6400900-006 | 1 | 20 | 1 |
| 29 | 6400200-015 | 2 | 20 | 1 | 29 | 6400900-007 | 0 | 20 | 0 |
| 29 | 6400200-016 | 2 | 20 | 1 | 29 | 6400900-008 | 1 | 20 | 1 |
| 29 | 6400200-019 | 4 | 20 | 4 | 29 | 6400900-009 | 2 | 20 | 1 |
| 29 | 6400200-021 | 8 | 20 | 4 | 29 | 6400900-010 | 0 | 20 | 0 |
| 29 | 6400200-022 | 0 | 20 | 0 | 29 | 6400900-011 | 2 | 20 | 1 |
| 29 | 6400200-023 | 1 | 20 | 1 | 29 | 6400900-012 | 3 | 20 | 3 |
| 29 | 6400200-024 | 4 | 20 | 3 | 29 | 6400900-013 | 0 | 20 | 0 |
| 29 | 6400200-025 | 2 | 20 | 2 | 29 | 6400900-014 | 0 | 20 | 0 |
| 29 | 6400200-026 | 1 | 20 | 1 | 29 | 6400900-015 | 1 | 20 | 1 |
| 29 | 6400200-027 | 2 | 20 | 1 | 29 | 6400900-016 | 1 | 20 | 1 |
| 29 | 6400200-028 | 0 | 20 | 0 | 29 | 6400900-017 | 4 | 20 | 3 |
| 29 | 6400200-029 | 0 | 20 | 0 | 29 | 6400900-018 | 1 | 20 | 1 |
| 29 | 6400200-030 | 1 | 19 | 1 | 29 | 6400900-019 | 2 | 20 | 2 |
| 29 | 6400200-031 | 0 | 20 | 0 | 29 | 6400900-020 | 1 | 20 | 1 |
| 29 | 6400200-032 | 1 | 20 | 1 | 29 | 6400900-058 | 1 | 20 | 1 |
| 29 | 6400900-021 | 3 | 20 | 1 | 29 | 6400900-059 | 1 | 20 | 1 |
| 29 | 6400900-022 | 0 | 20 | 0 | 29 | 6400900-060 | 1 | 20 | 1 |
| 29 | 6400900-023 | 0 | 1 | 0 | 29 | 6400900-061 | 0 | 20 | 0 |
| 29 | 6400900-024 | 3 | 20 | 2 | 29 | 6401200-004 | 0 | 20 | 0 |
| 29 | 6400900-025 | 0 | 20 | 0 | 29 | 6401200-006 | 5 | 20 | 3 |
| 29 | 6400900-028 | 0 | 20 | 0 | 29 | 6401200-007 | 5 | 20 | 2 |
| 29 | 6400900-029 | 1 | 20 | 1 | 29 | 6401200-008 | 0 | 20 | 0 |
| 29 | 6400900-030 | 2 | 20 | 1 | 29 | 6401200-010 | 1 | 20 | 1 |
| 29 | 6400900-031 | 1 | 20 | 1 | 29 | 6401200-011 | 4 | 20 | 4 |
| 29 | 6400900-032 | 0 | 20 | 0 | 29 | 6401200-012 | 0 | 20 | 0 |
| 29 | 6400900-033 | 0 | 20 | 0 | 29 | 6401200-013 | 3 | 20 | 2 |
| 29 | 6400900-034 | 1 | 20 | 1 | 29 | 6401200-014 | 1 | 20 | 1 |
| 29 | 6400900-035 | 4 | 20 | 3 | 29 | 6401200-015 | 0 | 20 | 0 |
| 29 | 6400900-036 | 0 | 20 | 0 | 29 | 6401200-018 | 0 | 20 | 0 |
| 29 | 6400900-037 | 1 | 20 | 1 | 29 | 6401200-021 | 2 | 20 | 2 |
| 29 | 6400900-038 | 1 | 20 | 1 | 29 | 6401200-022 | 1 | 20 | 1 |
| 29 | 6400900-039 | 0 | 20 | 0 | 29 | 6401200-024 | 0 | 17 | 0 |
| 29 | 6400900-040 | 0 | 20 | 0 | 29 | 6401200-030 | 3 | 20 | 3 |
| 29 | 6400900-041 | 0 | 1 | 0 | 29 | 6401200-031 | 0 | 20 | 0 |
| 29 | 6400900-042 | 1 | 20 | 1 | 29 | 6401200-035 | 6 | 20 | 2 |
| 29 | 6400900-043 | 0 | 20 | 0 | 29 | 6401200-037 | 1 | 20 | 1 |
| 29 | 6400900-044 | 6 | 20 | 6 | 29 | 6401200-039 | 1 | 20 | 1 |
| 29 | 6400900-045 | 1 | 20 | 1 | 29 | 6401200-040 | 0 | 20 | 0 |
| 29 | 6400900-046 | 4 | 20 | 3 | 29 | 6401200-041 | 2 | 20 | 2 |
| 29 | 6400900-047 | 1 | 20 | 1 | 29 | 6401200-044 | 1 | 20 | 1 |
| 29 | 6400900-048 | 0 | 2 | 0 | 29 | 6401200-045 | 5 | 1 | 1 |
| 29 | 6400900-049 | 0 | 1 | 0 | 29 | 6401200-046 | 0 | 20 | 0 |
| 29 | 6400900-050 | 4 | 20 | 3 | 29 | 6401200-047 | 0 | 2 | 0 |
| 29 | 6400900-051 | 0 | 20 | 0 | 29 | 6401200-048 | 2 | 20 | 1 |
| 29 | 6400900-052 | 0 | 20 | 0 | 29 | 6401200-050 | 3 | 20 | 3 |
| 29 | 6400900-053 | 0 | 20 | 0 | 29 | 6401200-051 | 2 | 20 | 1 |
| 29 | 6400900-054 | 1 | 20 | 1 | 29 | 6401200-053 | 2 | 20 | 2 |
| 29 | 6400900-055 | 0 | 20 | 0 | 29 | 6401200-056 | 1 | 19 | 1 |
| 29 | 6400900-056 | 0 | 20 | 0 | 29 | 6401200-057 | 3 | 20 | 3 |
| 29 | 6400900-057 | 0 | 1 | 0 | 29 | 6401200-058 | 1 | 20 | 1 |
| 29 | 6401200-059 | 0 | 20 | 0 | 30 | 6400900-003 | 0 | 20 | 0 |
| 29 | 6401200-060 | 0 | 20 | 0 | 30 | 6400900-004 | 0 | 20 | 0 |
| 29 | 6401200-061 | 4 | 20 | 3 | 30 | 6400900-005 | 2 | 20 | 2 |
| 29 | 6401200-062 | 1 | 20 | 1 | 30 | 6400900-006 | 1 | 20 | 1 |
| 29 | 6401200-063 | 4 | 20 | 4 | 30 | 6400900-007 | 0 | 20 | 0 |
| 30 | 6400100-006 | 2 | 20 | 2 | 30 | 6400900-008 | 1 | 20 | 1 |
| 30 | 6400200-008 | 0 | 20 | 0 | 30 | 6400900-009 | 2 | 20 | 1 |
| 30 | 6400200-009 | 0 | 20 | 0 | 30 | 6400900-010 | 0 | 20 | 0 |
| 30 | 6400200-010 | 1 | 20 | 1 | 30 | 6400900-011 | 2 | 20 | 1 |
| 30 | 6400200-011 | 2 | 20 | 2 | 30 | 6400900-012 | 3 | 20 | 3 |
| 30 | 6400200-012 | 3 | 20 | 3 | 30 | 6400900-013 | 0 | 20 | 0 |
| 30 | 6400200-014 | 3 | 20 | 2 | 30 | 6400900-014 | 0 | 20 | 0 |
| 30 | 6400200-021 | 8 | 20 | 4 | 30 | 6400900-015 | 1 | 20 | 1 |
| 30 | 6400200-024 | 4 | 20 | 3 | 30 | 6400900-016 | 1 | 20 | 1 |
| 30 | 6400200-025 | 2 | 20 | 2 | 30 | 6400900-017 | 4 | 20 | 3 |
| 30 | 6400200-026 | 1 | 20 | 1 | 30 | 6400900-018 | 1 | 20 | 1 |
| 30 | 6400200-029 | 0 | 20 | 0 | 30 | 6400900-019 | 2 | 20 | 2 |
| 30 | 6400200-030 | 1 | 19 | 1 | 30 | 6400900-020 | 1 | 20 | 1 |
| 30 | 6400200-031 | 0 | 20 | 0 | 30 | 6400900-021 | 3 | 20 | 1 |
| 30 | 6400200-032 | 1 | 20 | 1 | 30 | 6400900-022 | 0 | 20 | 0 |
| 30 | 6400200-033 | 2 | 20 | 1 | 30 | 6400900-023 | 0 | 20 | 0 |
| 30 | 6400200-034 | 0 | 20 | 0 | 30 | 6400900-024 | 3 | 20 | 2 |
| 30 | 6400200-035 | 2 | 20 | 2 | 30 | 6400900-025 | 0 | 20 | 0 |
| 30 | 6400200-036 | 1 | 20 | 1 | 30 | 6400900-028 | 0 | 20 | 0 |
| 30 | 6400200-037 | 2 | 20 | 1 | 30 | 6400900-029 | 1 | 20 | 1 |
| 30 | 6400200-038 | 12 | 20 | 12 | 30 | 6400900-030 | 2 | 20 | 1 |
| 30 | 6400300-017 | 57 | 30 | 19 | 30 | 6400900-031 | 1 | 20 | 1 |
| 30 | 6400500-002 | 0 | 20 | 0 | 30 | 6400900-032 | 0 | 20 | 0 |
| 30 | 6400500-087 | 0 | 20 | 0 | 30 | 6400900-033 | 0 | 20 | 0 |
| 30 | 6400800-007 | 0 | 20 | 0 | 30 | 6400900-034 | 1 | 20 | 1 |
| 30 | 6400800-017 | 1 | 20 | 1 | 30 | 6400900-035 | 4 | 20 | 3 |
| 30 | 6400800-019 | 1 | 20 | 1 | 30 | 6400900-036 | 0 | 20 | 0 |
| 30 | 6400800-035 | 0 | 20 | 0 | 30 | 6400900-037 | 1 | 20 | 1 |
| 30 | 6400800-039 | 0 | 20 | 0 | 30 | 6400900-038 | 1 | 20 | 1 |
| 30 | 6400800-045 | 0 | 20 | 0 | 30 | 6400900-039 | 0 | 20 | 0 |
| 30 | 6400900-001 | 0 | 20 | 0 | 30 | 6400900-040 | 0 | 20 | 0 |
| 30 | 6400900-002 | 0 | 20 | 0 | 30 | 6400900-041 | 0 | 20 | 0 |
| 30 | 6400900-042 | 1 | 20 | 1 | 30 | 6401200-031 | 0 | 20 | 0 |
| 30 | 6400900-043 | 0 | 20 | 0 | 30 | 6401200-035 | 6 | 20 | 2 |
| 30 | 6400900-044 | 6 | 20 | 6 | 30 | 6401200-039 | 1 | 20 | 1 |
| 30 | 6400900-045 | 1 | 20 | 1 | 30 | 6401200-040 | 0 | 20 | 0 |
| 30 | 6400900-046 | 4 | 20 | 3 | 30 | 6401200-041 | 2 | 20 | 2 |
| 30 | 6400900-047 | 1 | 20 | 1 | 30 | 6401200-044 | 1 | 20 | 1 |
| 30 | 6400900-048 | 0 | 20 | 0 | 30 | 6401200-045 | 15 | 30 | 6 |
| 30 | 6400900-049 | 0 | 20 | 0 | 30 | 6401200-046 | 0 | 20 | 0 |
| 30 | 6400900-050 | 4 | 20 | 3 | 30 | 6401200-047 | 6 | 18 | 6 |
| 30 | 6400900-051 | 0 | 20 | 0 | 30 | 6401200-048 | 2 | 20 | 1 |
| 30 | 6400900-052 | 0 | 20 | 0 | 30 | 6401200-050 | 3 | 20 | 3 |
| 30 | 6400900-053 | 0 | 20 | 0 | 30 | 6401200-051 | 2 | 20 | 1 |
| 30 | 6400900-054 | 1 | 20 | 1 | 30 | 6401200-053 | 2 | 20 | 2 |
| 30 | 6400900-055 | 0 | 20 | 0 | 30 | 6401200-056 | 1 | 19 | 1 |
| 30 | 6400900-056 | 0 | 20 | 0 | 30 | 6401200-057 | 3 | 20 | 3 |
| 30 | 6400900-057 | 0 | 20 | 0 | 30 | 6401200-058 | 1 | 20 | 1 |
| 30 | 6400900-058 | 1 | 20 | 1 | 30 | 6401200-059 | 0 | 20 | 0 |
| 30 | 6400900-059 | 1 | 20 | 1 | 30 | 6401200-060 | 0 | 20 | 0 |
| 30 | 6400900-060 | 1 | 20 | 1 | 30 | 6401200-061 | 4 | 20 | 3 |
| 30 | 6400900-061 | 0 | 20 | 0 | 30 | 6401200-062 | 1 | 20 | 1 |
| 30 | 6401200-004 | 0 | 20 | 0 | 30 | 6401200-063 | 4 | 20 | 4 |
| 30 | 6401200-006 | 5 | 20 | 3 | 31 | 6400100-006 | 1 | 20 | 1 |
| 30 | 6401200-007 | 5 | 20 | 2 | 31 | 6400200-011 | 2 | 20 | 2 |
| 30 | 6401200-008 | 0 | 20 | 0 | 31 | 6400200-036 | 1 | 20 | 1 |
| 30 | 6401200-010 | 1 | 20 | 1 | 31 | 6400200-038 | 12 | 20 | 12 |
| 30 | 6401200-011 | 4 | 20 | 4 | 31 | 6400300-017 | 57 | 30 | 19 |
| 30 | 6401200-012 | 0 | 20 | 0 | 31 | 6400500-031 | 0 | 20 | 0 |
| 30 | 6401200-013 | 3 | 20 | 2 | 31 | 6400500-087 | 0 | 20 | 0 |
| 30 | 6401200-014 | 1 | 20 | 1 | 31 | 6400800-007 | 0 | 20 | 0 |
| 30 | 6401200-015 | 0 | 20 | 0 | 31 | 6400800-017 | 1 | 20 | 1 |
| 30 | 6401200-018 | 0 | 20 | 0 | 31 | 6400800-035 | 0 | 20 | 0 |
| 30 | 6401200-021 | 2 | 20 | 2 | 31 | 6400800-045 | 0 | 20 | 0 |
| 30 | 6401200-022 | 1 | 20 | 1 | 31 | 6400900-001 | 0 | 20 | 0 |
| 30 | 6401200-024 | 0 | 17 | 0 | 31 | 6400900-002 | 0 | 20 | 0 |
| 30 | 6401200-026 | 0 | 2 | 0 | 31 | 6400900-003 | 0 | 20 | 0 |
| 30 | 6401200-030 | 3 | 20 | 3 | 31 | 6400900-004 | 0 | 20 | 0 |
| 31 | 6400900-005 | 2 | 20 | 2 | 31 | 6400900-043 | 0 | 20 | 0 |
| 31 | 6400900-006 | 1 | 20 | 1 | 31 | 6400900-044 | 6 | 20 | 6 |
| 31 | 6400900-007 | 0 | 20 | 0 | 31 | 6400900-045 | 0 | 1 | 0 |
| 31 | 6400900-008 | 1 | 20 | 1 | 31 | 6400900-046 | 4 | 20 | 3 |
| 31 | 6400900-009 | 2 | 20 | 1 | 31 | 6400900-047 | 1 | 20 | 1 |
| 31 | 6400900-010 | 0 | 20 | 0 | 31 | 6400900-048 | 0 | 20 | 0 |
| 31 | 6400900-011 | 2 | 20 | 1 | 31 | 6400900-049 | 0 | 20 | 0 |
| 31 | 6400900-012 | 3 | 20 | 3 | 31 | 6400900-050 | 4 | 20 | 3 |
| 31 | 6400900-013 | 0 | 1 | 0 | 31 | 6400900-051 | 0 | 20 | 0 |
| 31 | 6400900-014 | 0 | 20 | 0 | 31 | 6400900-052 | 0 | 20 | 0 |
| 31 | 6400900-015 | 1 | 20 | 1 | 31 | 6400900-053 | 0 | 20 | 0 |
| 31 | 6400900-016 | 1 | 20 | 1 | 31 | 6400900-054 | 1 | 20 | 1 |
| 31 | 6400900-017 | 4 | 20 | 3 | 31 | 6400900-055 | 0 | 20 | 0 |
| 31 | 6400900-018 | 1 | 20 | 1 | 31 | 6400900-056 | 0 | 20 | 0 |
| 31 | 6400900-019 | 2 | 20 | 2 | 31 | 6400900-057 | 0 | 20 | 0 |
| 31 | 6400900-020 | 1 | 20 | 1 | 31 | 6400900-058 | 1 | 20 | 1 |
| 31 | 6400900-021 | 3 | 20 | 1 | 31 | 6400900-059 | 1 | 20 | 1 |
| 31 | 6400900-022 | 0 | 20 | 0 | 31 | 6400900-060 | 1 | 20 | 1 |
| 31 | 6400900-023 | 0 | 20 | 0 | 31 | 6400900-061 | 0 | 20 | 0 |
| 31 | 6400900-024 | 3 | 20 | 2 | 31 | 6401200-001 | 0 | 20 | 0 |
| 31 | 6400900-025 | 0 | 20 | 0 | 31 | 6401200-003 | 0 | 20 | 0 |
| 31 | 6400900-028 | 0 | 20 | 0 | 31 | 6401200-004 | 1 | 20 | 1 |
| 31 | 6400900-029 | 1 | 20 | 1 | 31 | 6401200-005 | 2 | 20 | 1 |
| 31 | 6400900-030 | 2 | 20 | 1 | 31 | 6401200-006 | 0 | 20 | 0 |
| 31 | 6400900-031 | 1 | 20 | 1 | 31 | 6401200-007 | 5 | 20 | 2 |
| 31 | 6400900-032 | 0 | 20 | 0 | 31 | 6401200-008 | 0 | 20 | 0 |
| 31 | 6400900-033 | 0 | 20 | 0 | 31 | 6401200-010 | 0 | 20 | 0 |
| 31 | 6400900-034 | 1 | 20 | 1 | 31 | 6401200-013 | 0 | 20 | 0 |
| 31 | 6400900-035 | 4 | 20 | 3 | 31 | 6401200-014 | 1 | 20 | 1 |
| 31 | 6400900-036 | 0 | 20 | 0 | 31 | 6401200-015 | 0 | 20 | 0 |
| 31 | 6400900-037 | 1 | 20 | 1 | 31 | 6401200-017 | 2 | 20 | 1 |
| 31 | 6400900-038 | 1 | 20 | 1 | 31 | 6401200-018 | 0 | 20 | 0 |
| 31 | 6400900-039 | 0 | 20 | 0 | 31 | 6401200-020 | 2 | 20 | 1 |
| 31 | 6400900-040 | 0 | 20 | 0 | 31 | 6401200-021 | 0 | 20 | 0 |
| 31 | 6400900-041 | 0 | 20 | 0 | 31 | 6401200-022 | 1 | 20 | 1 |
| 31 | 6400900-042 | 1 | 20 | 1 | 31 | 6401200-025 | 1 | 19 | 1 |
| 31 | 6401200-026 | 0 | 2 | 0 | 32 | 6400900-013 | 0 | 1 | 0 |
| 31 | 6401200-030 | 3 | 20 | 3 | 32 | 6400900-045 | 0 | 1 | 0 |
| 31 | 6401200-031 | 0 | 20 | 0 | 32 | 6401200-001 | 0 | 20 | 0 |
| 31 | 6401200-035 | 0 | 20 | 0 | 32 | 6401200-003 | 0 | 20 | 0 |
| 31 | 6401200-036 | 1 | 20 | 1 | 32 | 6401200-004 | 1 | 20 | 1 |
| 31 | 6401200-038 | 0 | 20 | 0 | 32 | 6401200-005 | 2 | 20 | 1 |
| 31 | 6401200-039 | 1 | 20 | 1 | 32 | 6401200-006 | 0 | 20 | 0 |
| 31 | 6401200-041 | 2 | 20 | 2 | 32 | 6401200-010 | 0 | 20 | 0 |
| 31 | 6401200-042 | 1 | 20 | 1 | 32 | 6401200-013 | 0 | 20 | 0 |
| 31 | 6401200-044 | 1 | 20 | 1 | 32 | 6401200-015 | 0 | 20 | 0 |
| 31 | 6401200-045 | 0 | 10 | 0 | 32 | 6401200-017 | 2 | 20 | 1 |
| 31 | 6401200-046 | 0 | 17 | 0 | 32 | 6401200-020 | 2 | 20 | 1 |
| 31 | 6401200-047 | 6 | 18 | 6 | 32 | 6401200-021 | 0 | 20 | 0 |
| 31 | 6401200-048 | 2 | 20 | 1 | 32 | 6401200-025 | 1 | 19 | 1 |
| 31 | 6401200-050 | 3 | 20 | 3 | 32 | 6401200-026 | 2 | 18 | 2 |
| 31 | 6401200-051 | 0 | 20 | 0 | 32 | 6401200-035 | 0 | 20 | 0 |
| 31 | 6401200-053 | 2 | 20 | 2 | 32 | 6401200-036 | 1 | 20 | 1 |
| 31 | 6401200-057 | 3 | 20 | 3 | 32 | 6401200-038 | 0 | 20 | 0 |
| 31 | 6401200-059 | 0 | 20 | 0 | 32 | 6401200-040 | 0 | 20 | 0 |
| 31 | 6401200-061 | 0 | 20 | 0 | 32 | 6401200-042 | 1 | 20 | 1 |
| 31 | 6401200-062 | 0 | 20 | 0 | 32 | 6401200-045 | 0 | 10 | 0 |
| 31 | 6401200-063 | 0 | 20 | 0 | 32 | 6401200-046 | 0 | 17 | 0 |
| 31 | 6401200-064 | 0 | 20 | 0 | 32 | 6401200-051 | 0 | 20 | 0 |
| 31 | 6401200-066 | 1 | 20 | 1 | 32 | 6401200-054 | 0 | 3 | 0 |
| 31 | 6401200-067 | 5 | 20 | 2 | 32 | 6401200-061 | 0 | 20 | 0 |
| 31 | 6401200-068 | 0 | 20 | 0 | 32 | 6401200-062 | 0 | 20 | 0 |
| 31 | 6401200-069 | 3 | 20 | 2 | 32 | 6401200-063 | 0 | 20 | 0 |
| 31 | 6401200-070 | 4 | 20 | 4 | 32 | 6401200-064 | 0 | 20 | 0 |
| 31 | 6401200-071 | 0 | 20 | 0 | 32 | 6401200-066 | 1 | 20 | 1 |
| 31 | 6401200-078 | 2 | 20 | 2 | 32 | 6401200-067 | 5 | 20 | 2 |
| 32 | 6400100-006 | 1 | 20 | 1 | 32 | 6401200-068 | 0 | 20 | 0 |
| 32 | 6400500-031 | 0 | 20 | 0 | 32 | 6401200-069 | 3 | 20 | 2 |
| 32 | 6400500-087 | 0 | 20 | 0 | 32 | 6401200-070 | 4 | 20 | 4 |
| 32 | 6400800-035 | 0 | 20 | 0 | 32 | 6401200-071 | 0 | 20 | 0 |
| 32 | 6400800-045 | 0 | 20 | 0 | 32 | 6401200-073 | 0 | 1 | 0 |
| 32 | 6400900-009 | 0 | 1 | 0 | 32 | 6401200-078 | 2 | 20 | 2 |
| 32 | 6400900-012 | 0 | 1 | 0 | 33 | 6400500-031 | 0 | 20 | 0 |
| 33 | 6400700-001 | 2 | 10 | 1 | 33 | 6401200-073 | 0 | 1 | 0 |
| 33 | 6400900-009 | 0 | 1 | 0 | 33 | 6401200-074 | 1 | 20 | 1 |
| 33 | 6400900-012 | 0 | 1 | 0 | 33 | 6401200-075 | 1 | 20 | 1 |
| 33 | 6401200-001 | 0 | 20 | 0 | 33 | 6401200-076 | 0 | 20 | 0 |
| 33 | 6401200-003 | 0 | 20 | 0 | 33 | 6401200-077 | 0 | 20 | 0 |
| 33 | 6401200-005 | 2 | 20 | 1 | 33 | 6401200-078 | 2 | 20 | 2 |
| 33 | 6401200-009 | 2 | 20 | 2 | 34 | 6400700-001 | 2 | 10 | 1 |
| 33 | 6401200-016 | 6 | 20 | 3 | 34 | 6400800-030 | 1 | 20 | 1 |
| 33 | 6401200-017 | 2 | 20 | 1 | 34 | 6400800-040 | 0 | 20 | 0 |
| 33 | 6401200-019 | 0 | 20 | 0 | 34 | 6400800-051 | 1 | 20 | 1 |
| 33 | 6401200-020 | 2 | 20 | 1 | 34 | 6400800-052 | 0 | 20 | 0 |
| 33 | 6401200-022 | 0 | 20 | 0 | 34 | 6400800-053 | 0 | 20 | 0 |
| 33 | 6401200-025 | 1 | 19 | 1 | 34 | 6400800-054 | 0 | 20 | 0 |
| 33 | 6401200-026 | 2 | 18 | 2 | 34 | 6400800-056 | 3 | 20 | 2 |
| 33 | 6401200-027 | 4 | 20 | 2 | 34 | 6400800-058 | 1 | 20 | 1 |
| 33 | 6401200-028 | 1 | 20 | 1 | 34 | 6400800-059 | 1 | 20 | 1 |
| 33 | 6401200-029 | 1 | 20 | 1 | 34 | 6400800-060 | 0 | 20 | 0 |
| 33 | 6401200-033 | 1 | 20 | 1 | 34 | 6400800-062 | 1 | 20 | 1 |
| 33 | 6401200-034 | 0 | 20 | 0 | 34 | 6400800-063 | 1 | 20 | 1 |
| 33 | 6401200-036 | 1 | 20 | 1 | 34 | 6400800-064 | 1 | 20 | 1 |
| 33 | 6401200-038 | 0 | 20 | 0 | 34 | 6400800-065 | 1 | 20 | 1 |
| 33 | 6401200-040 | 0 | 20 | 0 | 34 | 6400800-066 | 3 | 20 | 2 |
| 33 | 6401200-042 | 1 | 20 | 1 | 34 | 6400800-067 | 1 | 20 | 1 |
| 33 | 6401200-043 | 4 | 20 | 3 | 34 | 6400800-068 | 1 | 20 | 1 |
| 33 | 6401200-049 | 0 | 20 | 0 | 34 | 6400800-069 | 0 | 20 | 0 |
| 33 | 6401200-054 | 0 | 3 | 0 | 34 | 6400900-009 | 0 | 1 | 0 |
| 33 | 6401200-064 | 0 | 20 | 0 | 34 | 6401200-002 | 5 | 20 | 4 |
| 33 | 6401200-065 | 2 | 20 | 2 | 34 | 6401200-009 | 2 | 20 | 2 |
| 33 | 6401200-066 | 1 | 20 | 1 | 34 | 6401200-016 | 6 | 20 | 3 |
| 33 | 6401200-067 | 5 | 20 | 2 | 34 | 6401200-019 | 0 | 20 | 0 |
| 33 | 6401200-068 | 0 | 20 | 0 | 34 | 6401200-022 | 0 | 20 | 0 |
| 33 | 6401200-069 | 3 | 20 | 2 | 34 | 6401200-027 | 4 | 20 | 2 |
| 33 | 6401200-070 | 4 | 20 | 4 | 34 | 6401200-028 | 1 | 20 | 1 |
| 33 | 6401200-071 | 0 | 20 | 0 | 34 | 6401200-029 | 1 | 20 | 1 |
| 33 | 6401200-072 | 7 | 20 | 6 | 34 | 6401200-032 | 0 | 20 | 0 |
| 34 | 6401200-033 | 1 | 20 | 1 | 35 | 6400800-059 | 1 | 20 | 1 |
| 34 | 6401200-034 | 0 | 20 | 0 | 35 | 6400800-060 | 0 | 20 | 0 |
| 34 | 6401200-037 | 3 | 20 | 3 | 35 | 6400800-062 | 1 | 20 | 1 |
| 34 | 6401200-040 | 0 | 20 | 0 | 35 | 6400800-063 | 1 | 20 | 1 |
| 34 | 6401200-043 | 4 | 20 | 3 | 35 | 6400800-064 | 1 | 20 | 1 |
| 34 | 6401200-049 | 0 | 20 | 0 | 35 | 6400800-065 | 1 | 20 | 1 |
| 34 | 6401200-054 | 7 | 17 | 6 | 35 | 6400800-066 | 3 | 20 | 2 |
| 34 | 6401200-055 | 3 | 20 | 1 | 35 | 6400800-067 | 1 | 20 | 1 |
| 34 | 6401200-065 | 2 | 20 | 2 | 35 | 6400800-068 | 1 | 20 | 1 |
| 34 | 6401200-072 | 7 | 20 | 6 | 35 | 6400800-069 | 0 | 20 | 0 |
| 34 | 6401200-073 | 2 | 19 | 1 | 35 | 6400900-003 | 0 | 1 | 0 |
| 34 | 6401200-074 | 1 | 20 | 1 | 35 | 6400900-011 | 3 | 7 | 1 |
| 34 | 6401200-075 | 1 | 20 | 1 | 35 | 6400900-048 | 0 | 1 | 0 |
| 34 | 6401200-076 | 0 | 20 | 0 | 35 | 6401200-002 | 5 | 20 | 4 |
| 34 | 6401200-077 | 0 | 20 | 0 | 35 | 6401200-009 | 2 | 20 | 2 |
| 35 | 6400100-006 | 5 | 20 | 3 | 35 | 6401200-016 | 6 | 20 | 3 |
| 35 | 6400300-040 | 0 | 20 | 0 | 35 | 6401200-019 | 0 | 20 | 0 |
| 35 | 6400300-042 | 4 | 20 | 4 | 35 | 6401200-022 | 0 | 20 | 0 |
| 35 | 6400500-018 | 0 | 20 | 0 | 35 | 6401200-027 | 4 | 20 | 2 |
| 35 | 6400500-023 | 1 | 20 | 1 | 35 | 6401200-028 | 1 | 20 | 1 |
| 35 | 6400700-001 | 2 | 10 | 1 | 35 | 6401200-029 | 1 | 20 | 1 |
| 35 | 6400800-030 | 1 | 20 | 1 | 35 | 6401200-032 | 0 | 20 | 0 |
| 35 | 6400800-039 | 0 | 20 | 0 | 35 | 6401200-033 | 1 | 20 | 1 |
| 35 | 6400800-040 | 0 | 20 | 0 | 35 | 6401200-034 | 0 | 20 | 0 |
| 35 | 6400800-045 | 0 | 20 | 0 | 35 | 6401200-037 | 3 | 20 | 3 |
| 35 | 6400800-046 | 2 | 20 | 1 | 35 | 6401200-043 | 4 | 20 | 3 |
| 35 | 6400800-047 | 2 | 20 | 1 | 35 | 6401200-045 | 0 | 1 | 0 |
| 35 | 6400800-048 | 0 | 20 | 0 | 35 | 6401200-049 | 0 | 20 | 0 |
| 35 | 6400800-049 | 5 | 20 | 2 | 35 | 6401200-054 | 7 | 17 | 6 |
| 35 | 6400800-051 | 1 | 20 | 1 | 35 | 6401200-055 | 3 | 20 | 1 |
| 35 | 6400800-052 | 0 | 20 | 0 | 35 | 6401200-065 | 2 | 20 | 2 |
| 35 | 6400800-053 | 0 | 20 | 0 | 35 | 6401200-072 | 7 | 20 | 6 |
| 35 | 6400800-054 | 0 | 20 | 0 | 35 | 6401200-073 | 2 | 19 | 1 |
| 35 | 6400800-055 | 2 | 20 | 1 | 35 | 6401200-074 | 1 | 20 | 1 |
| 35 | 6400800-056 | 3 | 20 | 2 | 35 | 6401200-075 | 1 | 20 | 1 |
| 35 | 6400800-058 | 1 | 20 | 1 | 35 | 6401200-076 | 0 | 20 | 0 |
| 35 | 6401200-077 | 0 | 20 | 0 | 36 | 6400800-062 | 1 | 20 | 1 |
| 36 | 6400100-006 | 5 | 20 | 3 | 36 | 6400800-063 | 1 | 20 | 1 |
| 36 | 6400300-040 | 0 | 20 | 0 | 36 | 6400800-064 | 1 | 20 | 1 |
| 36 | 6400300-042 | 4 | 20 | 4 | 36 | 6400800-065 | 1 | 20 | 1 |
| 36 | 6400500-018 | 0 | 20 | 0 | 36 | 6400800-066 | 3 | 20 | 2 |
| 36 | 6400500-023 | 1 | 20 | 1 | 36 | 6400800-067 | 1 | 20 | 1 |
| 36 | 6400800-028 | 1 | 20 | 1 | 36 | 6400800-068 | 1 | 20 | 1 |
| 36 | 6400800-030 | 1 | 20 | 1 | 36 | 6400800-069 | 0 | 20 | 0 |
| 36 | 6400800-033 | 1 | 20 | 1 | 36 | 6400900-003 | 0 | 1 | 0 |
| 36 | 6400800-034 | 3 | 20 | 3 | 36 | 6400900-011 | 3 | 7 | 1 |
| 36 | 6400800-035 | 2 | 20 | 1 | 36 | 6400900-013 | 0 | 1 | 0 |
| 36 | 6400800-036 | 3 | 20 | 2 | 36 | 6400900-031 | 0 | 1 | 0 |
| 36 | 6400800-037 | 0 | 20 | 0 | 36 | 6400900-048 | 0 | 1 | 0 |
| 36 | 6400800-038 | 2 | 20 | 1 | 36 | 6401200-002 | 5 | 20 | 4 |
| 36 | 6400800-039 | 0 | 20 | 0 | 36 | 6401200-032 | 0 | 20 | 0 |
| 36 | 6400800-040 | 0 | 20 | 0 | 36 | 6401200-037 | 3 | 20 | 3 |
| 36 | 6400800-041 | 1 | 20 | 1 | 36 | 6401200-045 | 0 | 1 | 0 |
| 36 | 6400800-042 | 0 | 20 | 0 | 36 | 6401200-055 | 3 | 20 | 1 |
| 36 | 6400800-043 | 3 | 20 | 1 | 37 | 6400100-006 | 5 | 20 | 3 |
| 36 | 6400800-044 | 11 | 20 | 6 | 37 | 6400300-040 | 0 | 20 | 0 |
| 36 | 6400800-045 | 0 | 20 | 0 | 37 | 6400300-042 | 4 | 20 | 4 |
| 36 | 6400800-046 | 2 | 20 | 1 | 37 | 6400500-018 | 0 | 20 | 0 |
| 36 | 6400800-047 | 2 | 20 | 1 | 37 | 6400500-023 | 1 | 20 | 1 |
| 36 | 6400800-048 | 0 | 20 | 0 | 37 | 6400800-001 | 3 | 20 | 2 |
| 36 | 6400800-049 | 5 | 20 | 2 | 37 | 6400800-002 | 3 | 20 | 2 |
| 36 | 6400800-050 | 2 | 20 | 2 | 37 | 6400800-003 | 4 | 20 | 3 |
| 36 | 6400800-051 | 1 | 20 | 1 | 37 | 6400800-004 | 0 | 20 | 0 |
| 36 | 6400800-052 | 0 | 20 | 0 | 37 | 6400800-008 | 7 | 20 | 6 |
| 36 | 6400800-053 | 0 | 20 | 0 | 37 | 6400800-009 | 2 | 20 | 1 |
| 36 | 6400800-054 | 0 | 20 | 0 | 37 | 6400800-010 | 0 | 20 | 0 |
| 36 | 6400800-055 | 2 | 20 | 1 | 37 | 6400800-011 | 3 | 20 | 3 |
| 36 | 6400800-056 | 3 | 20 | 2 | 37 | 6400800-012 | 2 | 20 | 2 |
| 36 | 6400800-058 | 1 | 20 | 1 | 37 | 6400800-013 | 0 | 20 | 0 |
| 36 | 6400800-059 | 1 | 20 | 1 | 37 | 6400800-014 | 0 | 20 | 0 |
| 36 | 6400800-060 | 0 | 20 | 0 | 37 | 6400800-015 | 3 | 20 | 1 |
| 36 | 6400800-061 | 1 | 20 | 1 | 37 | 6400800-016 | 2 | 20 | 2 |
| 37 | 6400800-017 | 1 | 20 | 1 | 37 | 6401200-045 | 0 | 1 | 0 |
| 37 | 6400800-018 | 0 | 20 | 0 | 38 | 6400300-001 | 1 | 20 | 1 |
| 37 | 6400800-019 | 3 | 20 | 2 | 38 | 6400300-003 | 2 | 20 | 1 |
| 37 | 6400800-020 | 3 | 20 | 3 | 38 | 6400300-004 | 0 | 20 | 0 |
| 37 | 6400800-021 | 2 | 20 | 2 | 38 | 6400300-005 | 0 | 20 | 0 |
| 37 | 6400800-022 | 3 | 20 | 3 | 38 | 6400300-006 | 5 | 20 | 3 |
| 37 | 6400800-024 | 3 | 20 | 2 | 38 | 6400300-007 | 1 | 20 | 1 |
| 37 | 6400800-025 | 0 | 20 | 0 | 38 | 6400300-009 | 1 | 20 | 1 |
| 37 | 6400800-026 | 4 | 20 | 2 | 38 | 6400300-011 | 1 | 20 | 1 |
| 37 | 6400800-028 | 1 | 20 | 1 | 38 | 6400300-013 | 4 | 10 | 2 |
| 37 | 6400800-032 | 10 | 20 | 3 | 38 | 6400300-017 | 2 | 10 | 2 |
| 37 | 6400800-033 | 1 | 20 | 1 | 38 | 6400300-019 | 1 | 20 | 1 |
| 37 | 6400800-034 | 3 | 20 | 3 | 38 | 6400300-021 | 1 | 20 | 1 |
| 37 | 6400800-035 | 2 | 20 | 1 | 38 | 6400300-022 | 3 | 20 | 3 |
| 37 | 6400800-036 | 3 | 20 | 2 | 38 | 6400300-023 | 2 | 20 | 1 |
| 37 | 6400800-037 | 0 | 20 | 0 | 38 | 6400300-024 | 8 | 20 | 5 |
| 37 | 6400800-038 | 2 | 20 | 1 | 38 | 6400300-026 | 7 | 20 | 4 |
| 37 | 6400800-039 | 0 | 20 | 0 | 38 | 6400300-027 | 2 | 20 | 2 |
| 37 | 6400800-041 | 1 | 20 | 1 | 38 | 6400300-028 | 8 | 20 | 6 |
| 37 | 6400800-042 | 0 | 20 | 0 | 38 | 6400500-017 | 1 | 20 | 1 |
| 37 | 6400800-043 | 3 | 20 | 1 | 38 | 6400800-001 | 3 | 20 | 2 |
| 37 | 6400800-044 | 11 | 20 | 6 | 38 | 6400800-002 | 3 | 20 | 2 |
| 37 | 6400800-045 | 0 | 20 | 0 | 38 | 6400800-003 | 4 | 20 | 3 |
| 37 | 6400800-046 | 2 | 20 | 1 | 38 | 6400800-004 | 0 | 20 | 0 |
| 37 | 6400800-047 | 2 | 20 | 1 | 38 | 6400800-005 | 3 | 20 | 3 |
| 37 | 6400800-048 | 0 | 20 | 0 | 38 | 6400800-006 | 2 | 20 | 2 |
| 37 | 6400800-049 | 5 | 20 | 2 | 38 | 6400800-007 | 1 | 20 | 1 |
| 37 | 6400800-050 | 2 | 20 | 2 | 38 | 6400800-008 | 7 | 20 | 6 |
| 37 | 6400800-055 | 2 | 20 | 1 | 38 | 6400800-009 | 2 | 20 | 1 |
| 37 | 6400800-061 | 1 | 20 | 1 | 38 | 6400800-010 | 0 | 20 | 0 |
| 37 | 6400900-003 | 0 | 1 | 0 | 38 | 6400800-011 | 3 | 20 | 3 |
| 37 | 6400900-011 | 3 | 7 | 1 | 38 | 6400800-012 | 2 | 20 | 2 |
| 37 | 6400900-013 | 0 | 1 | 0 | 38 | 6400800-013 | 0 | 20 | 0 |
| 37 | 6400900-031 | 0 | 1 | 0 | 38 | 6400800-014 | 0 | 20 | 0 |
| 37 | 6400900-048 | 0 | 1 | 0 | 38 | 6400800-015 | 3 | 20 | 1 |
| 37 | 6400900-049 | 0 | 1 | 0 | 38 | 6400800-016 | 2 | 20 | 2 |
| 38 | 6400800-017 | 1 | 20 | 1 | 39 | 6400300-006 | 5 | 20 | 3 |
| 38 | 6400800-018 | 0 | 20 | 0 | 39 | 6400300-007 | 1 | 20 | 1 |
| 38 | 6400800-019 | 3 | 20 | 2 | 39 | 6400300-009 | 1 | 20 | 1 |
| 38 | 6400800-020 | 3 | 20 | 3 | 39 | 6400300-011 | 1 | 20 | 1 |
| 38 | 6400800-021 | 2 | 20 | 2 | 39 | 6400300-012 | 5 | 10 | 3 |
| 38 | 6400800-022 | 3 | 20 | 3 | 39 | 6400300-013 | 4 | 10 | 2 |
| 38 | 6400800-023 | 3 | 20 | 3 | 39 | 6400300-015 | 0 | 20 | 0 |
| 38 | 6400800-024 | 3 | 20 | 2 | 39 | 6400300-016 | 4 | 20 | 2 |
| 38 | 6400800-025 | 0 | 20 | 0 | 39 | 6400300-017 | 2 | 10 | 2 |
| 38 | 6400800-026 | 4 | 20 | 2 | 39 | 6400300-019 | 1 | 20 | 1 |
| 38 | 6400800-028 | 1 | 20 | 1 | 39 | 6400300-021 | 1 | 20 | 1 |
| 38 | 6400800-031 | 3 | 20 | 3 | 39 | 6400300-022 | 3 | 20 | 3 |
| 38 | 6400800-032 | 10 | 20 | 3 | 39 | 6400300-023 | 2 | 20 | 1 |
| 38 | 6400800-033 | 1 | 20 | 1 | 39 | 6400300-024 | 8 | 20 | 5 |
| 38 | 6400800-034 | 3 | 20 | 3 | 39 | 6400300-025 | 2 | 20 | 2 |
| 38 | 6400800-035 | 2 | 20 | 1 | 39 | 6400300-026 | 7 | 20 | 4 |
| 38 | 6400800-036 | 3 | 20 | 2 | 39 | 6400300-027 | 2 | 20 | 2 |
| 38 | 6400800-037 | 0 | 20 | 0 | 39 | 6400300-028 | 8 | 20 | 6 |
| 38 | 6400800-038 | 2 | 20 | 1 | 39 | 6400300-031 | 1 | 20 | 1 |
| 38 | 6400800-041 | 1 | 20 | 1 | 39 | 6400300-032 | 0 | 20 | 0 |
| 38 | 6400800-042 | 0 | 20 | 0 | 39 | 6400300-035 | 3 | 10 | 2 |
| 38 | 6400800-043 | 3 | 20 | 1 | 39 | 6400300-036 | 3 | 20 | 1 |
| 38 | 6400800-044 | 11 | 20 | 6 | 39 | 6400300-037 | 0 | 10 | 0 |
| 38 | 6400800-050 | 2 | 20 | 2 | 39 | 6400300-038 | 3 | 10 | 1 |
| 38 | 6400800-057 | 1 | 20 | 1 | 39 | 6400300-039 | 1 | 20 | 1 |
| 38 | 6400800-061 | 1 | 20 | 1 | 39 | 6400300-041 | 2 | 20 | 1 |
| 38 | 6400900-013 | 0 | 1 | 0 | 39 | 6400300-044 | 1 | 20 | 1 |
| 38 | 6400900-031 | 0 | 1 | 0 | 39 | 6400500-007 | 1 | 20 | 1 |
| 38 | 6400900-048 | 0 | 1 | 0 | 39 | 6400500-017 | 1 | 20 | 1 |
| 38 | 6400900-049 | 0 | 1 | 0 | 39 | 6400500-046 | 0 | 20 | 0 |
| 38 | 6401200-068 | 0 | 20 | 0 | 39 | 6400600-028 | 0 | 20 | 0 |
| 39 | 6400200-026 | 2 | 20 | 2 | 39 | 6400800-001 | 3 | 20 | 2 |
| 39 | 6400300-001 | 1 | 20 | 1 | 39 | 6400800-002 | 3 | 20 | 2 |
| 39 | 6400300-003 | 2 | 20 | 1 | 39 | 6400800-003 | 4 | 20 | 3 |
| 39 | 6400300-004 | 0 | 20 | 0 | 39 | 6400800-004 | 0 | 20 | 0 |
| 39 | 6400300-005 | 0 | 20 | 0 | 39 | 6400800-005 | 3 | 20 | 3 |
| 39 | 6400800-006 | 2 | 20 | 2 | 40 | 6400300-007 | 1 | 20 | 1 |
| 39 | 6400800-007 | 1 | 20 | 1 | 40 | 6400300-008 | 3 | 20 | 2 |
| 39 | 6400800-008 | 7 | 20 | 6 | 40 | 6400300-009 | 1 | 20 | 1 |
| 39 | 6400800-009 | 2 | 20 | 1 | 40 | 6400300-011 | 1 | 20 | 1 |
| 39 | 6400800-010 | 0 | 20 | 0 | 40 | 6400300-012 | 5 | 10 | 3 |
| 39 | 6400800-011 | 3 | 20 | 3 | 40 | 6400300-013 | 4 | 10 | 2 |
| 39 | 6400800-012 | 2 | 20 | 2 | 40 | 6400300-015 | 0 | 20 | 0 |
| 39 | 6400800-013 | 0 | 20 | 0 | 40 | 6400300-016 | 4 | 20 | 2 |
| 39 | 6400800-014 | 0 | 20 | 0 | 40 | 6400300-017 | 2 | 10 | 2 |
| 39 | 6400800-015 | 3 | 20 | 1 | 40 | 6400300-019 | 1 | 20 | 1 |
| 39 | 6400800-016 | 2 | 20 | 2 | 40 | 6400300-021 | 1 | 20 | 1 |
| 39 | 6400800-017 | 1 | 20 | 1 | 40 | 6400300-022 | 3 | 20 | 3 |
| 39 | 6400800-018 | 0 | 20 | 0 | 40 | 6400300-023 | 2 | 20 | 1 |
| 39 | 6400800-019 | 3 | 20 | 2 | 40 | 6400300-024 | 8 | 20 | 5 |
| 39 | 6400800-020 | 3 | 20 | 3 | 40 | 6400300-025 | 2 | 20 | 2 |
| 39 | 6400800-021 | 2 | 20 | 2 | 40 | 6400300-026 | 7 | 20 | 4 |
| 39 | 6400800-022 | 3 | 20 | 3 | 40 | 6400300-027 | 2 | 20 | 2 |
| 39 | 6400800-023 | 3 | 20 | 3 | 40 | 6400300-028 | 8 | 20 | 6 |
| 39 | 6400800-024 | 3 | 20 | 2 | 40 | 6400300-031 | 1 | 20 | 1 |
| 39 | 6400800-025 | 0 | 20 | 0 | 40 | 6400300-032 | 0 | 20 | 0 |
| 39 | 6400800-026 | 4 | 20 | 2 | 40 | 6400300-034 | 0 | 20 | 0 |
| 39 | 6400800-031 | 3 | 20 | 3 | 40 | 6400300-035 | 3 | 10 | 2 |
| 39 | 6400800-032 | 10 | 20 | 3 | 40 | 6400300-036 | 3 | 20 | 1 |
| 39 | 6400800-057 | 1 | 20 | 1 | 40 | 6400300-037 | 0 | 10 | 0 |
| 39 | 6400900-014 | 0 | 1 | 0 | 40 | 6400300-038 | 3 | 10 | 1 |
| 39 | 6400900-048 | 0 | 1 | 0 | 40 | 6400300-039 | 1 | 20 | 1 |
| 39 | 6400900-049 | 0 | 1 | 0 | 40 | 6400300-040 | 1 | 20 | 1 |
| 39 | 6401200-045 | 0 | 1 | 0 | 40 | 6400300-041 | 2 | 20 | 1 |
| 39 | 6401200-068 | 0 | 20 | 0 | 40 | 6400300-042 | 0 | 20 | 0 |
| 40 | 6400200-026 | 2 | 20 | 2 | 40 | 6400300-043 | 0 | 20 | 0 |
| 40 | 6400300-001 | 1 | 20 | 1 | 40 | 6400300-044 | 1 | 20 | 1 |
| 40 | 6400300-002 | 1 | 20 | 1 | 40 | 6400500-007 | 1 | 20 | 1 |
| 40 | 6400300-003 | 2 | 20 | 1 | 40 | 6400500-017 | 1 | 20 | 1 |
| 40 | 6400300-004 | 0 | 20 | 0 | 40 | 6400500-046 | 0 | 20 | 0 |
| 40 | 6400300-005 | 0 | 20 | 0 | 40 | 6400600-004 | 0 | 20 | 0 |
| 40 | 6400300-006 | 5 | 20 | 3 | 40 | 6400600-005 | 0 | 20 | 0 |
| 40 | 6400600-028 | 0 | 20 | 0 | 41 | 6400300-035 | 3 | 10 | 2 |
| 40 | 6400700-001 | 0 | 10 | 0 | 41 | 6400300-036 | 3 | 20 | 1 |
| 40 | 6400700-002 | 0 | 10 | 0 | 41 | 6400300-037 | 0 | 10 | 0 |
| 40 | 6400700-003 | 0 | 10 | 0 | 41 | 6400300-038 | 3 | 10 | 1 |
| 40 | 6400700-004 | 0 | 10 | 0 | 41 | 6400300-039 | 1 | 20 | 1 |
| 40 | 6400700-006 | 0 | 20 | 0 | 41 | 6400300-040 | 1 | 20 | 1 |
| 40 | 6400700-007 | 0 | 20 | 0 | 41 | 6400300-041 | 2 | 20 | 1 |
| 40 | 6400700-012 | 0 | 20 | 0 | 41 | 6400300-042 | 0 | 20 | 0 |
| 40 | 6400700-014 | 0 | 10 | 0 | 41 | 6400300-043 | 0 | 20 | 0 |
| 40 | 6400700-015 | 0 | 10 | 0 | 41 | 6400300-044 | 1 | 20 | 1 |
| 40 | 6400700-016 | 0 | 10 | 0 | 41 | 6400500-007 | 1 | 20 | 1 |
| 40 | 6400700-018 | 0 | 10 | 0 | 41 | 6400500-046 | 0 | 20 | 0 |
| 40 | 6400800-005 | 3 | 20 | 3 | 41 | 6400600-001 | 0 | 20 | 0 |
| 40 | 6400800-006 | 2 | 20 | 2 | 41 | 6400600-002 | 2 | 20 | 2 |
| 40 | 6400800-007 | 1 | 20 | 1 | 41 | 6400600-003 | 0 | 20 | 0 |
| 40 | 6400800-023 | 3 | 20 | 3 | 41 | 6400600-004 | 0 | 20 | 0 |
| 40 | 6400800-031 | 3 | 20 | 3 | 41 | 6400600-005 | 0 | 20 | 0 |
| 40 | 6400800-057 | 1 | 20 | 1 | 41 | 6400600-008 | 0 | 20 | 0 |
| 40 | 6400900-014 | 0 | 1 | 0 | 41 | 6400600-011 | 2 | 20 | 1 |
| 40 | 6400900-028 | 2 | 20 | 2 | 41 | 6400600-012 | 0 | 10 | 0 |
| 40 | 6400900-029 | 0 | 20 | 0 | 41 | 6400600-025 | 0 | 20 | 0 |
| 40 | 6400900-047 | 0 | 1 | 0 | 41 | 6400600-028 | 0 | 20 | 0 |
| 40 | 6400900-048 | 0 | 1 | 0 | 41 | 6400600-030 | 0 | 20 | 0 |
| 40 | 6401200-045 | 0 | 1 | 0 | 41 | 6400600-031 | 3 | 20 | 2 |
| 40 | 6401200-068 | 0 | 20 | 0 | 41 | 6400700-001 | 0 | 10 | 0 |
| 41 | 6400200-026 | 2 | 20 | 2 | 41 | 6400700-002 | 0 | 10 | 0 |
| 41 | 6400300-002 | 1 | 20 | 1 | 41 | 6400700-003 | 0 | 10 | 0 |
| 41 | 6400300-008 | 3 | 20 | 2 | 41 | 6400700-004 | 0 | 10 | 0 |
| 41 | 6400300-012 | 5 | 10 | 3 | 41 | 6400700-006 | 0 | 20 | 0 |
| 41 | 6400300-015 | 0 | 20 | 0 | 41 | 6400700-007 | 0 | 20 | 0 |
| 41 | 6400300-016 | 4 | 20 | 2 | 41 | 6400700-008 | 0 | 10 | 0 |
| 41 | 6400300-025 | 2 | 20 | 2 | 41 | 6400700-009 | 0 | 10 | 0 |
| 41 | 6400300-031 | 1 | 20 | 1 | 41 | 6400700-010 | 1 | 10 | 1 |
| 41 | 6400300-032 | 0 | 20 | 0 | 41 | 6400700-011 | 0 | 10 | 0 |
| 41 | 6400300-034 | 0 | 20 | 0 | 41 | 6400700-012 | 0 | 20 | 0 |
| 41 | 6400700-013 | 0 | 20 | 0 | 42 | 6400600-013 | 4 | 20 | 3 |
| 41 | 6400700-014 | 0 | 10 | 0 | 42 | 6400600-014 | 8 | 20 | 4 |
| 41 | 6400700-015 | 0 | 10 | 0 | 42 | 6400600-016 | 2 | 20 | 2 |
| 41 | 6400700-016 | 0 | 10 | 0 | 42 | 6400600-017 | 5 | 20 | 3 |
| 41 | 6400700-017 | 0 | 20 | 0 | 42 | 6400600-021 | 1 | 20 | 1 |
| 41 | 6400700-018 | 0 | 10 | 0 | 42 | 6400600-024 | 4 | 20 | 4 |
| 41 | 6400900-005 | 1 | 3 | 1 | 42 | 6400600-025 | 0 | 20 | 0 |
| 41 | 6400900-014 | 0 | 1 | 0 | 42 | 6400600-026 | 0 | 20 | 0 |
| 41 | 6400900-028 | 2 | 20 | 2 | 42 | 6400600-027 | 5 | 20 | 2 |
| 41 | 6400900-029 | 0 | 20 | 0 | 42 | 6400600-028 | 0 | 20 | 0 |
| 41 | 6400900-047 | 0 | 1 | 0 | 42 | 6400600-029 | 9 | 20 | 6 |
| 41 | 6400900-051 | 0 | 2 | 0 | 42 | 6400600-030 | 0 | 20 | 0 |
| 41 | 6400900-058 | 3 | 2 | 1 | 42 | 6400600-031 | 3 | 20 | 2 |
| 41 | 6401200-045 | 0 | 1 | 0 | 42 | 6400600-032 | 3 | 20 | 3 |
| 42 | 6400200-017 | 0 | 20 | 0 | 42 | 6400700-001 | 0 | 10 | 0 |
| 42 | 6400300-002 | 1 | 20 | 1 | 42 | 6400700-002 | 0 | 10 | 0 |
| 42 | 6400300-008 | 3 | 20 | 2 | 42 | 6400700-003 | 0 | 10 | 0 |
| 42 | 6400300-017 | 22 | 20 | 10 | 42 | 6400700-004 | 0 | 10 | 0 |
| 42 | 6400300-034 | 0 | 20 | 0 | 42 | 6400700-006 | 0 | 20 | 0 |
| 42 | 6400300-040 | 1 | 20 | 1 | 42 | 6400700-007 | 0 | 20 | 0 |
| 42 | 6400300-042 | 0 | 20 | 0 | 42 | 6400700-008 | 0 | 10 | 0 |
| 42 | 6400300-043 | 0 | 20 | 0 | 42 | 6400700-009 | 0 | 10 | 0 |
| 42 | 6400500-079 | 1 | 19 | 1 | 42 | 6400700-010 | 1 | 10 | 1 |
| 42 | 6400500-081 | 0 | 20 | 0 | 42 | 6400700-011 | 0 | 10 | 0 |
| 42 | 6400500-082 | 2 | 10 | 2 | 42 | 6400700-012 | 0 | 20 | 0 |
| 42 | 6400500-083 | 3 | 20 | 2 | 42 | 6400700-013 | 0 | 20 | 0 |
| 42 | 6400500-085 | 0 | 20 | 0 | 42 | 6400700-014 | 0 | 10 | 0 |
| 42 | 6400500-087 | 0 | 20 | 0 | 42 | 6400700-015 | 0 | 10 | 0 |
| 42 | 6400500-088 | 2 | 20 | 1 | 42 | 6400700-016 | 0 | 10 | 0 |
| 42 | 6400600-001 | 0 | 20 | 0 | 42 | 6400700-017 | 0 | 20 | 0 |
| 42 | 6400600-002 | 2 | 20 | 2 | 42 | 6400700-018 | 0 | 10 | 0 |
| 42 | 6400600-003 | 0 | 20 | 0 | 42 | 6400900-002 | 0 | 20 | 0 |
| 42 | 6400600-004 | 0 | 20 | 0 | 42 | 6400900-003 | 5 | 20 | 3 |
| 42 | 6400600-005 | 0 | 20 | 0 | 42 | 6400900-004 | 2 | 21 | 2 |
| 42 | 6400600-008 | 0 | 20 | 0 | 42 | 6400900-005 | 1 | 3 | 1 |
| 42 | 6400600-011 | 2 | 20 | 1 | 42 | 6400900-006 | 0 | 20 | 0 |
| 42 | 6400600-012 | 0 | 10 | 0 | 42 | 6400900-007 | 3 | 20 | 3 |
| 42 | 6400900-010 | 3 | 20 | 3 | 42 | 6400900-039 | 2 | 20 | 1 |
| 42 | 6400900-011 | 4 | 20 | 3 | 42 | 6400900-040 | 5 | 20 | 3 |
| 42 | 6400900-013 | 1 | 20 | 1 | 42 | 6400900-041 | 3 | 20 | 2 |
| 42 | 6400900-014 | 1 | 20 | 1 | 42 | 6400900-042 | 4 | 20 | 2 |
| 42 | 6400900-015 | 2 | 40 | 2 | 42 | 6400900-043 | 3 | 20 | 2 |
| 42 | 6400900-016 | 1 | 20 | 1 | 42 | 6400900-044 | 11 | 20 | 5 |
| 42 | 6400900-018 | 6 | 20 | 4 | 42 | 6400900-045 | 3 | 20 | 3 |
| 42 | 6400900-019 | 5 | 20 | 4 | 42 | 6400900-047 | 0 | 1 | 0 |
| 42 | 6400900-021 | 0 | 20 | 0 | 42 | 6400900-048 | 15 | 20 | 7 |
| 42 | 6400900-022 | 4 | 20 | 3 | 42 | 6400900-049 | 2 | 20 | 1 |
| 42 | 6400900-023 | 1 | 20 | 1 | 42 | 6400900-051 | 0 | 2 | 0 |
| 42 | 6400900-025 | 1 | 20 | 1 | 42 | 6400900-052 | 3 | 20 | 3 |
| 42 | 6400900-028 | 2 | 20 | 2 | 42 | 6400900-053 | 6 | 20 | 4 |
| 42 | 6400900-029 | 2 | 20 | 2 | 42 | 6400900-054 | 0 | 20 | 0 |
| 42 | 6400900-030 | 0 | 20 | 0 | 42 | 6400900-055 | 3 | 20 | 3 |
| 42 | 6400900-031 | 1 | 20 | 1 | 42 | 6400900-056 | 1 | 20 | 1 |
| 42 | 6400900-032 | 4 | 20 | 3 | 42 | 6400900-058 | 3 | 2 | 1 |
| 42 | 6400900-033 | 4 | 20 | 3 | 42 | 6400900-059 | 0 | 20 | 0 |
| 42 | 6400900-034 | 4 | 20 | 3 | 42 | 6400900-060 | 0 | 20 | 0 |
| 42 | 6400900-035 | 3 | 20 | 2 | 42 | 6401200-044 | 2 | 20 | 2 |
| 42 | 6400900-038 | 0 | 20 | 0 |  |  |  |  |  |


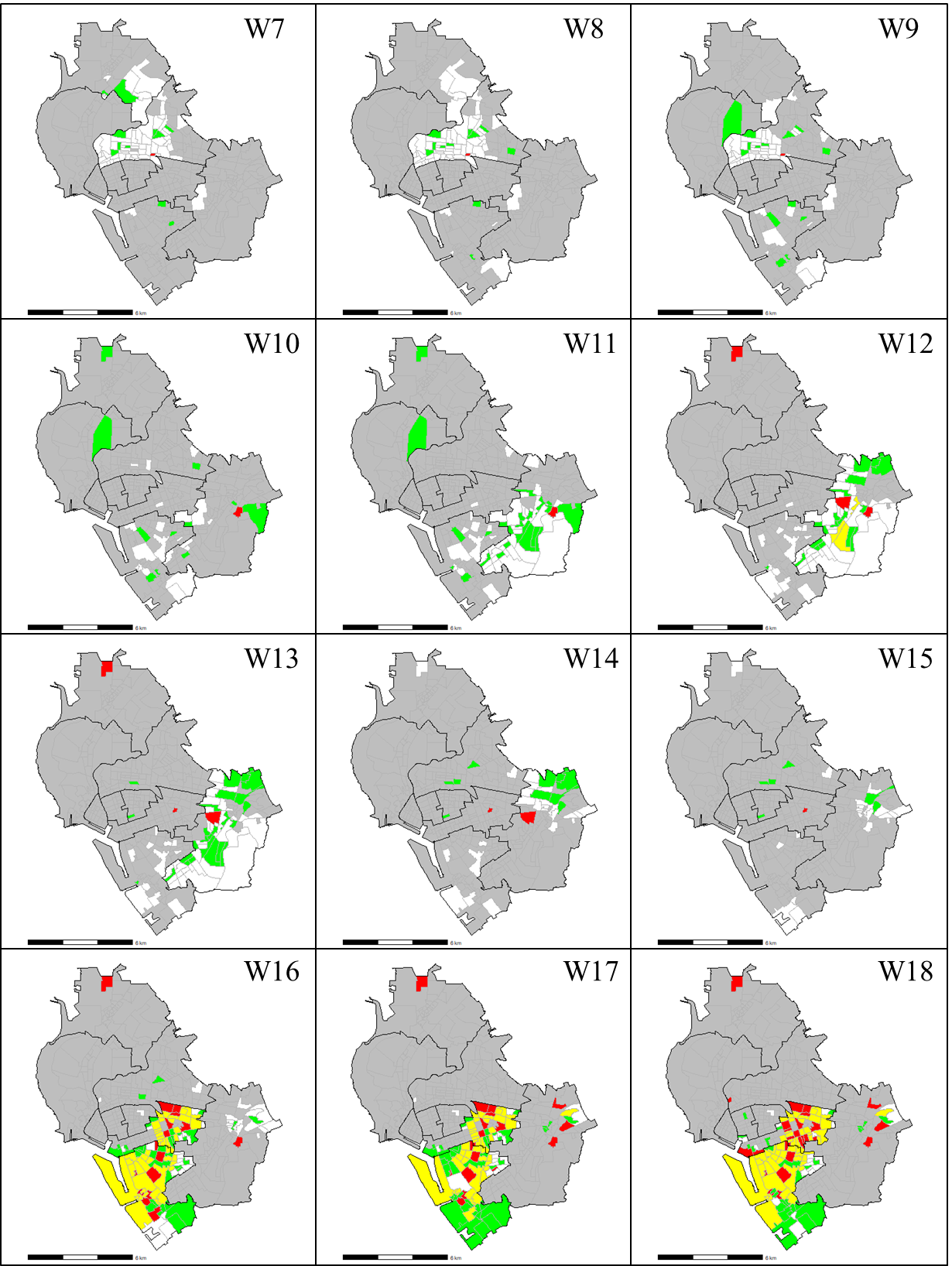
**Figure A1. Risk maps for *Aedes* population densities computed by the AI method** **with the second-order neighborhood structure.**


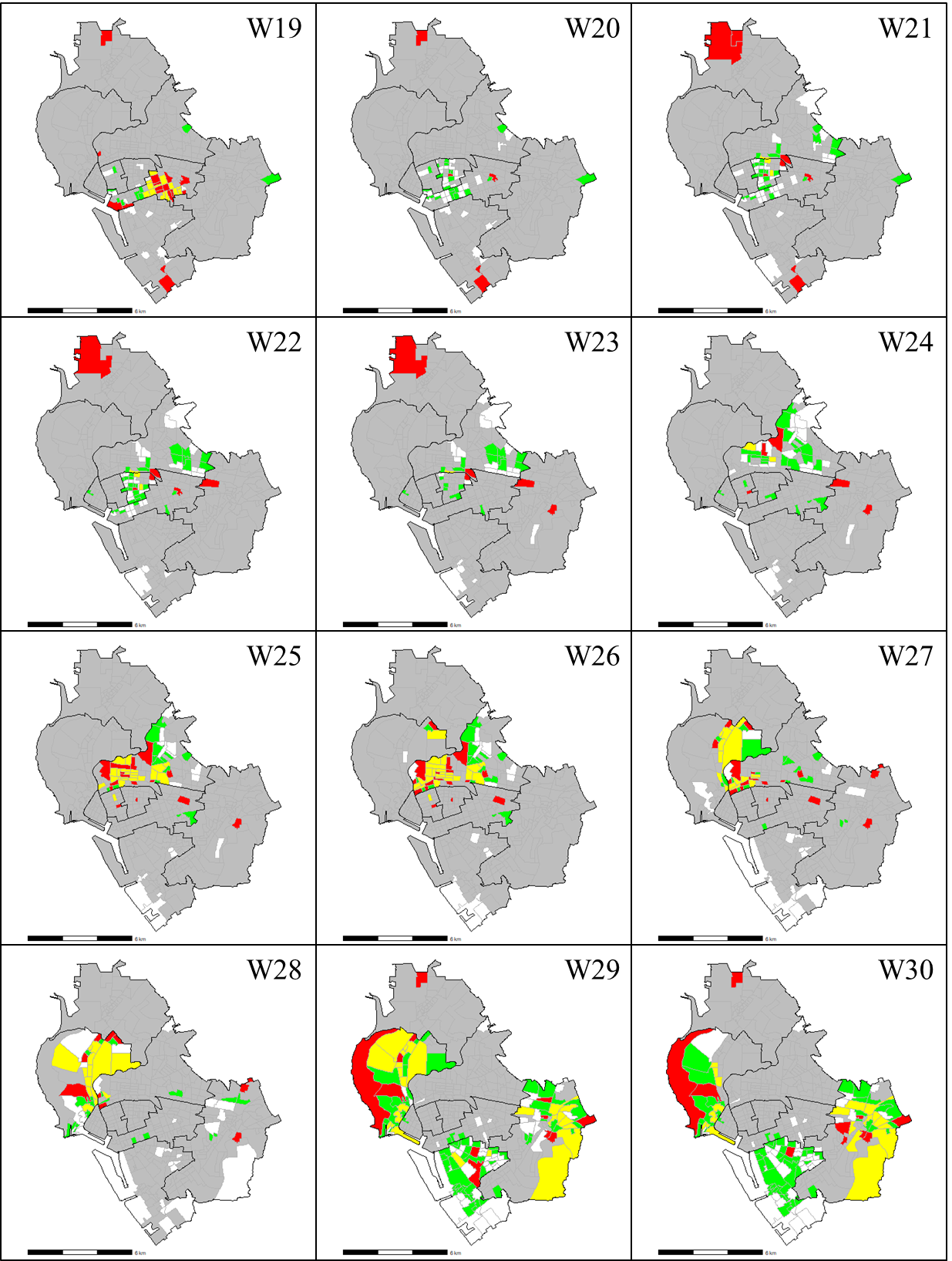


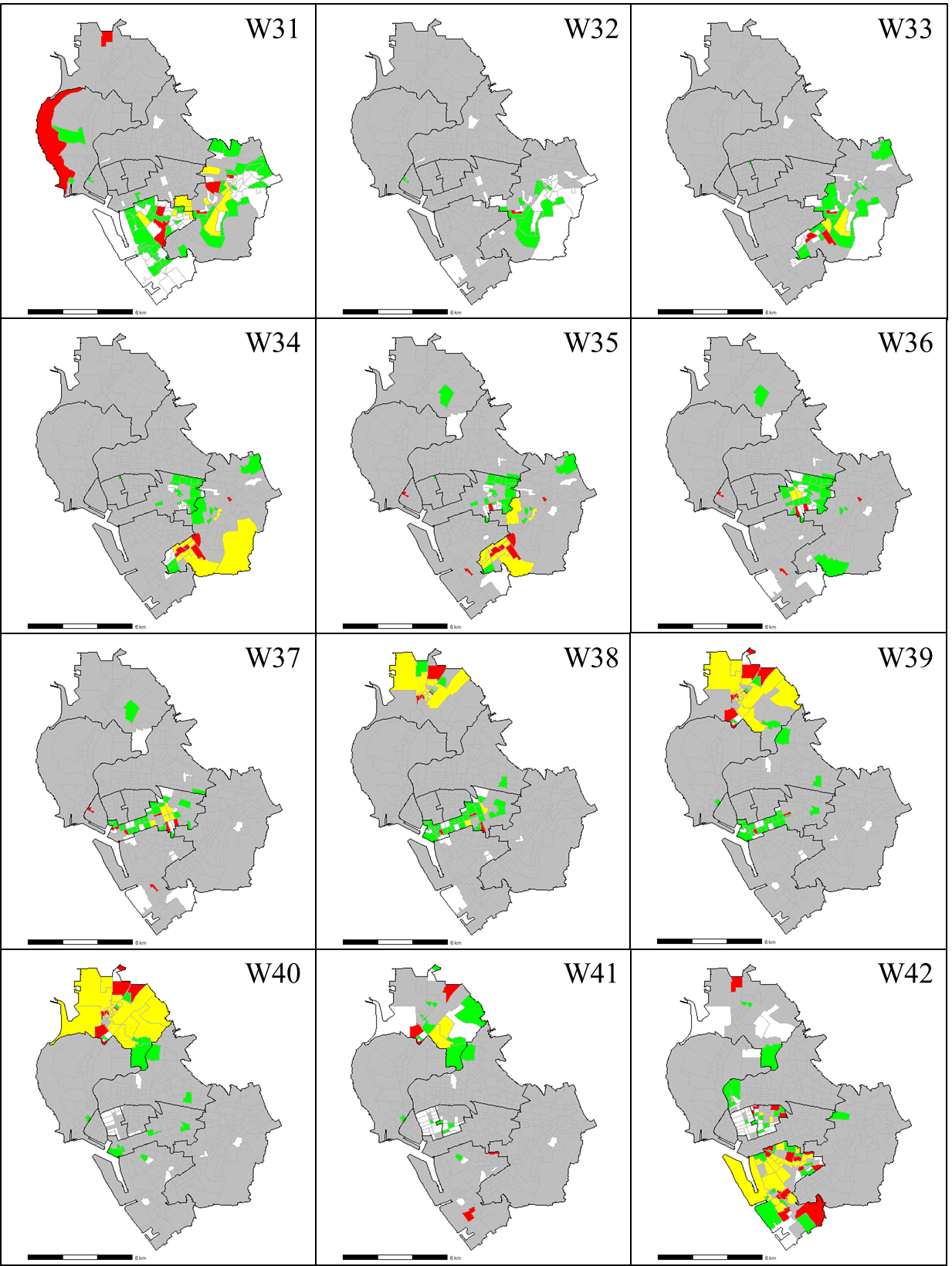


**Fig A2. Comparisons of risk maps for *Aedes* population densities computed by the AI, HK, and KDE methods**.


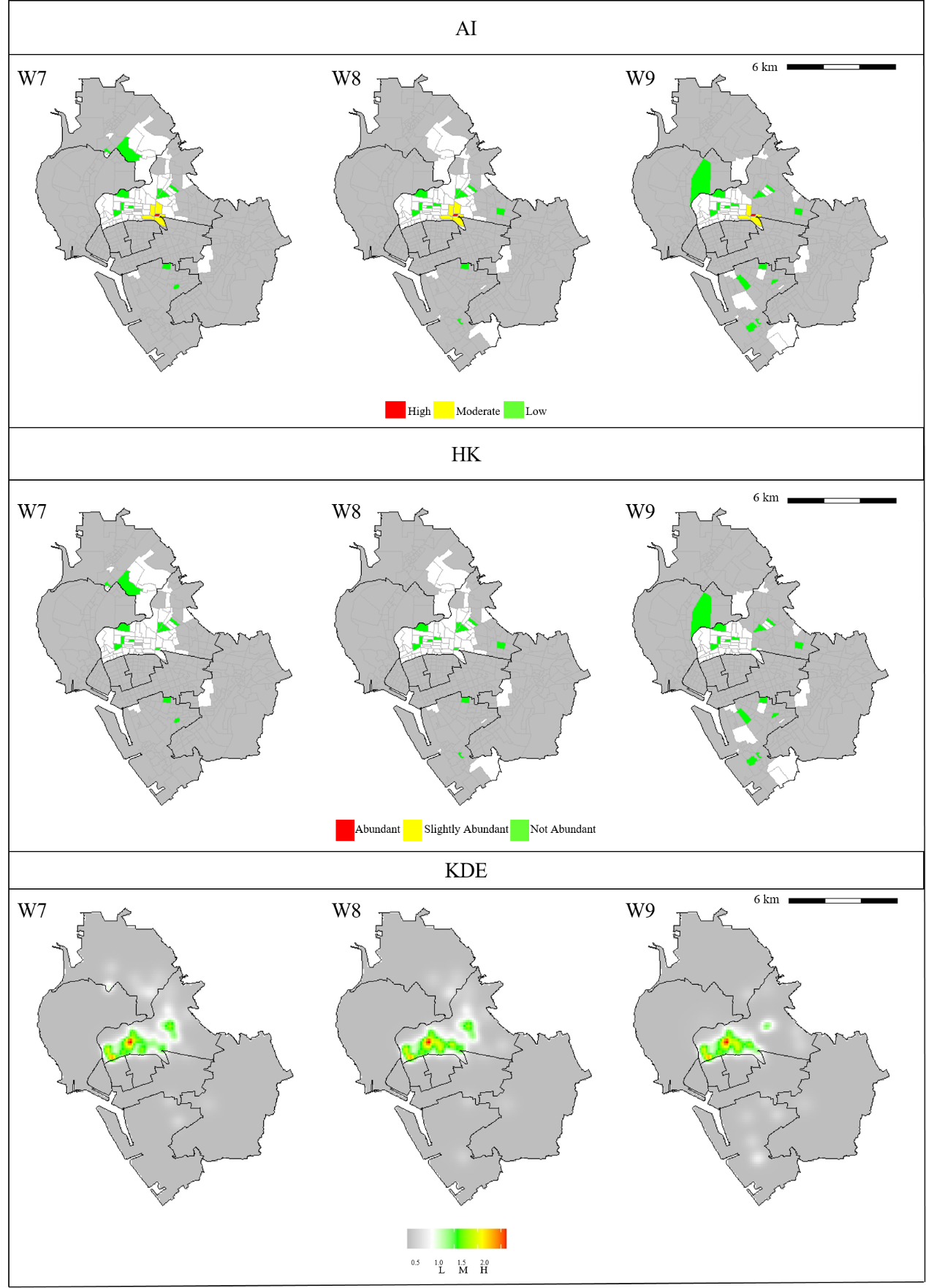


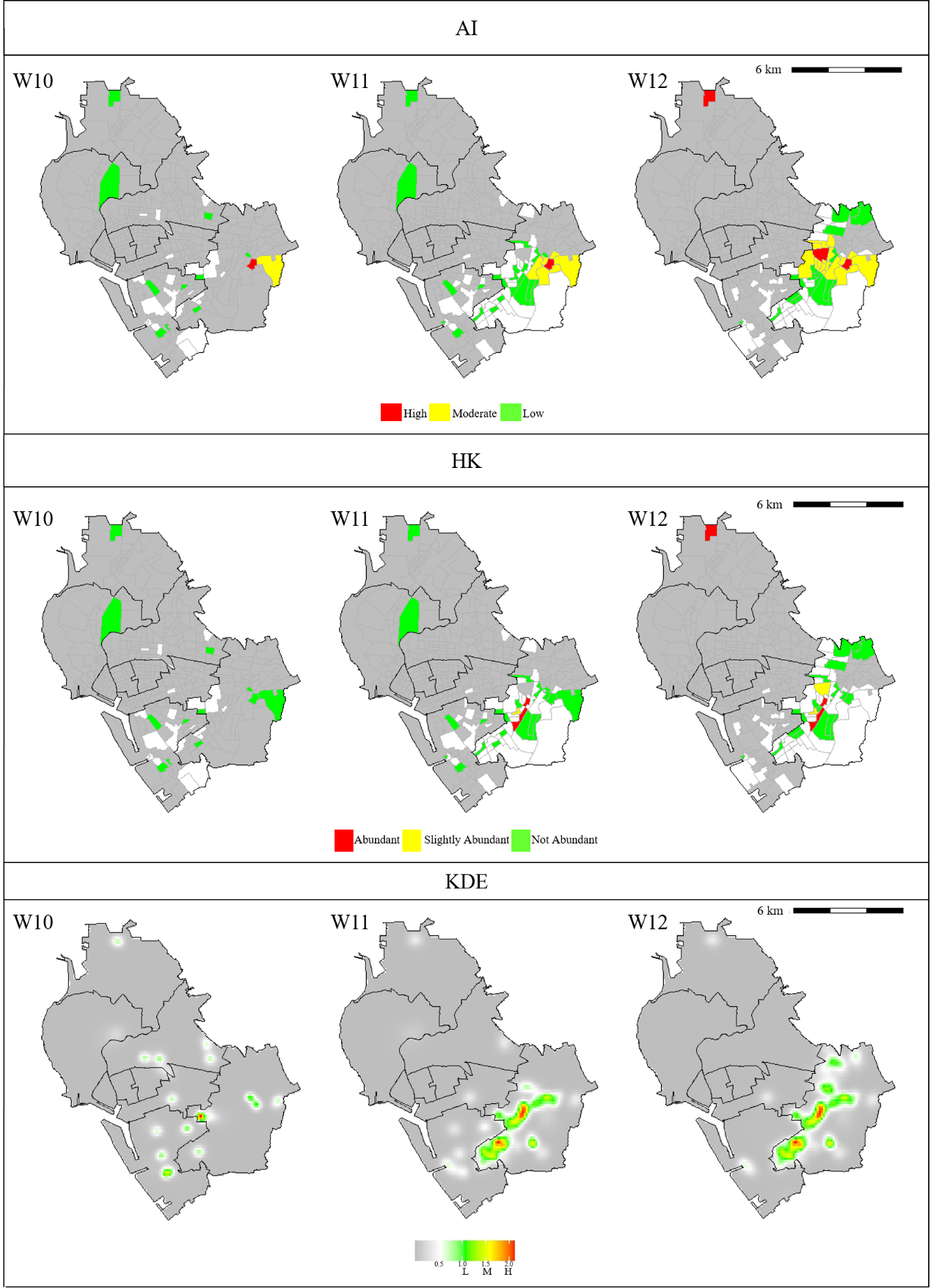


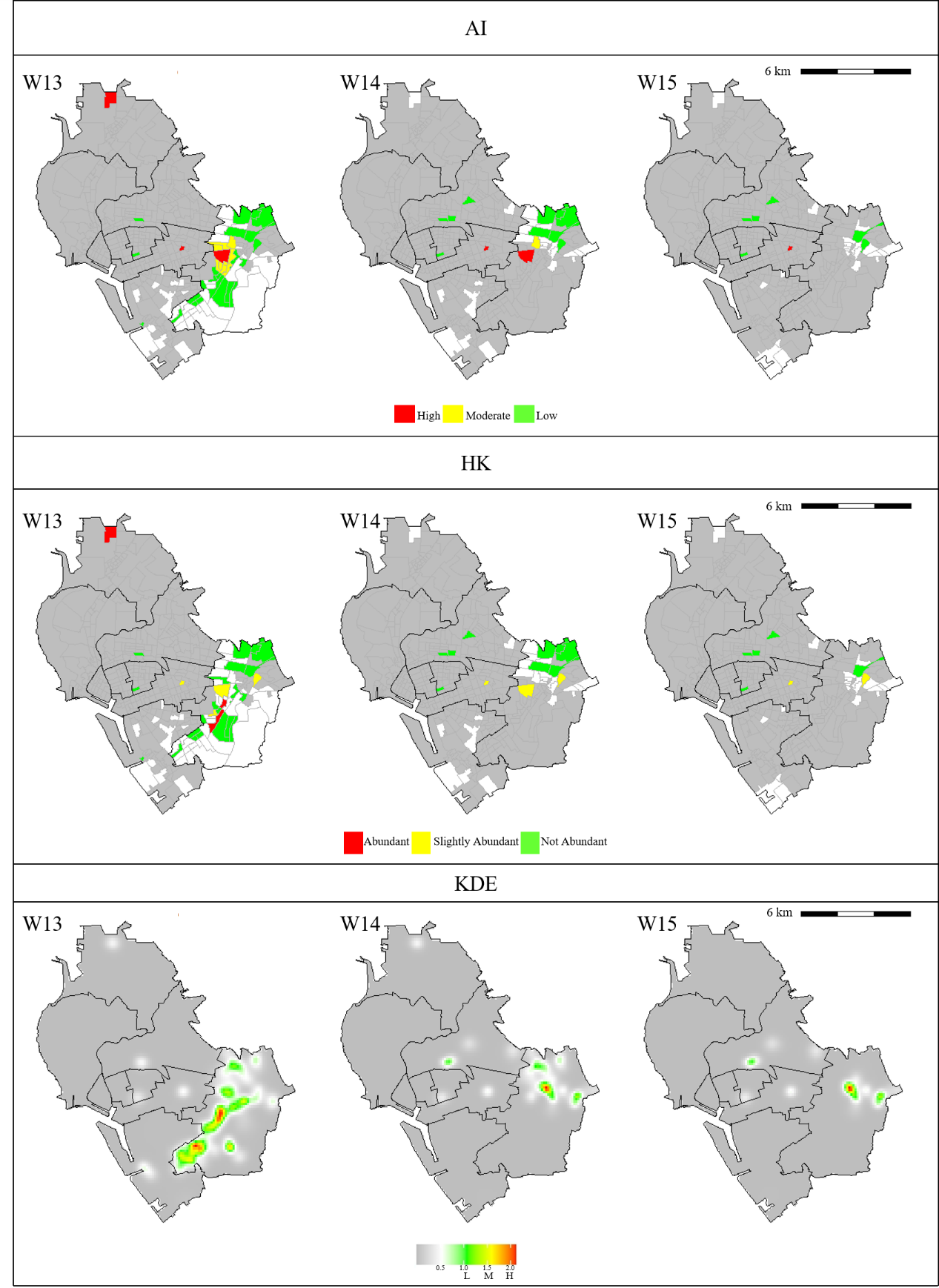


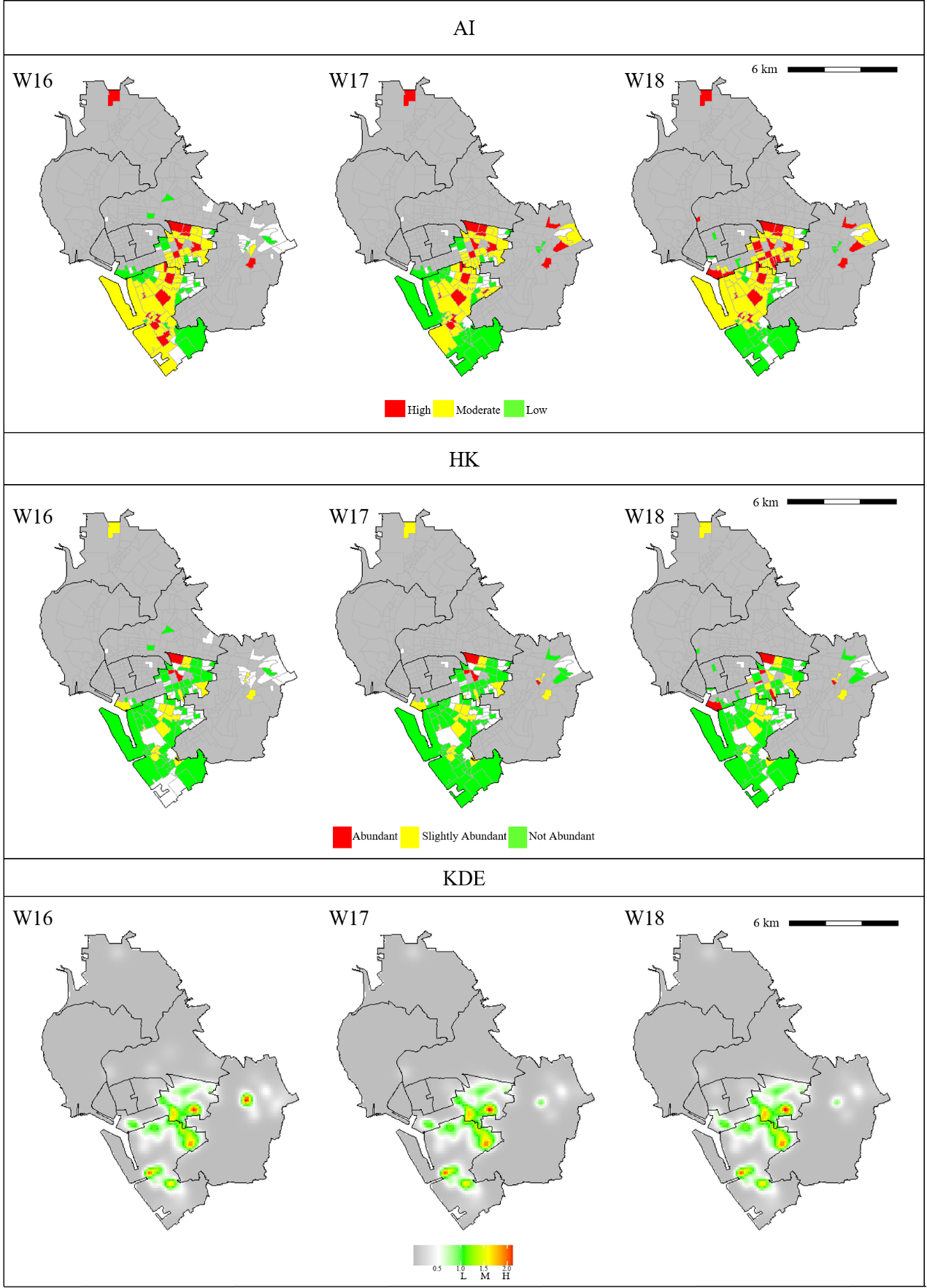


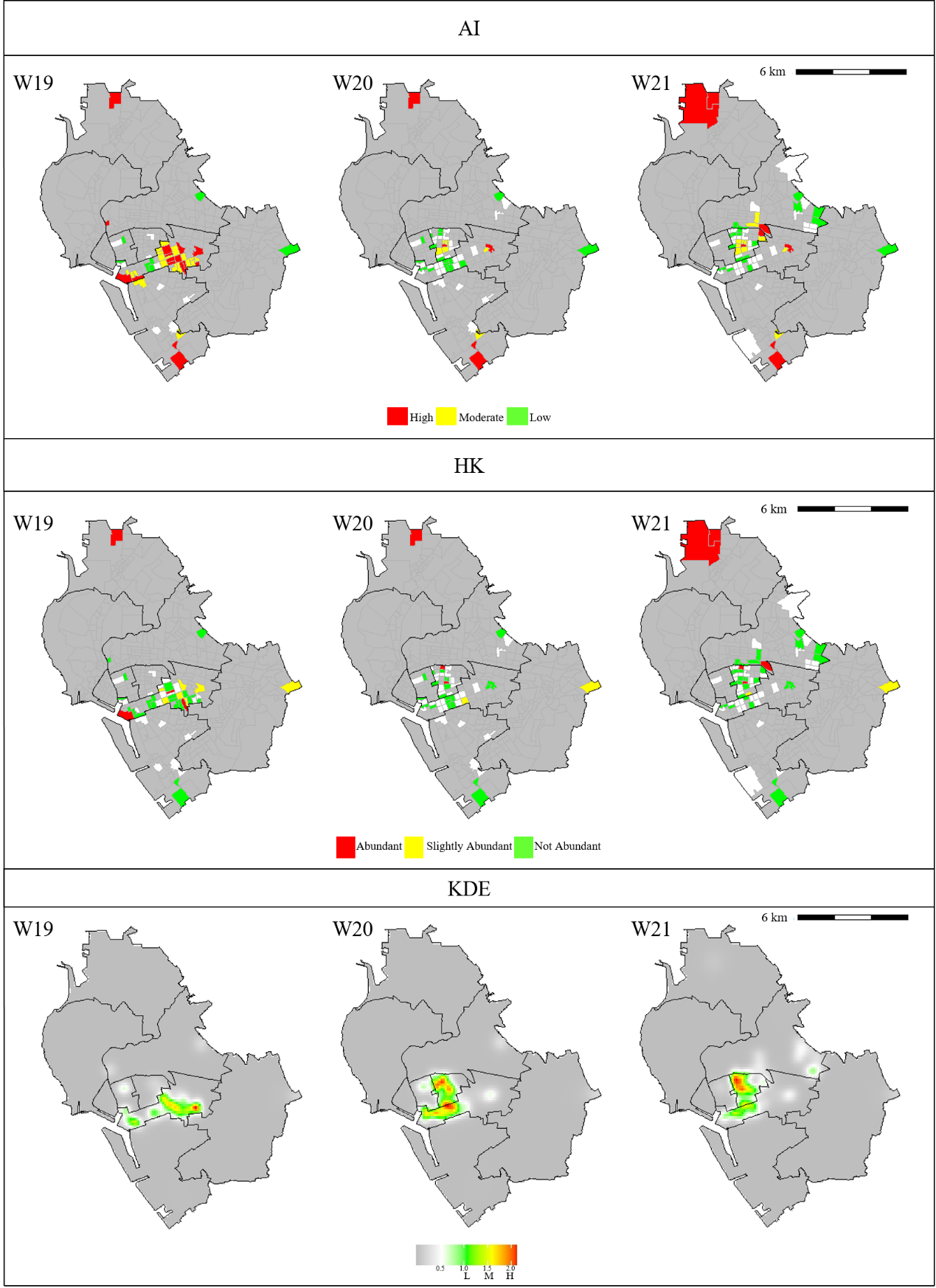


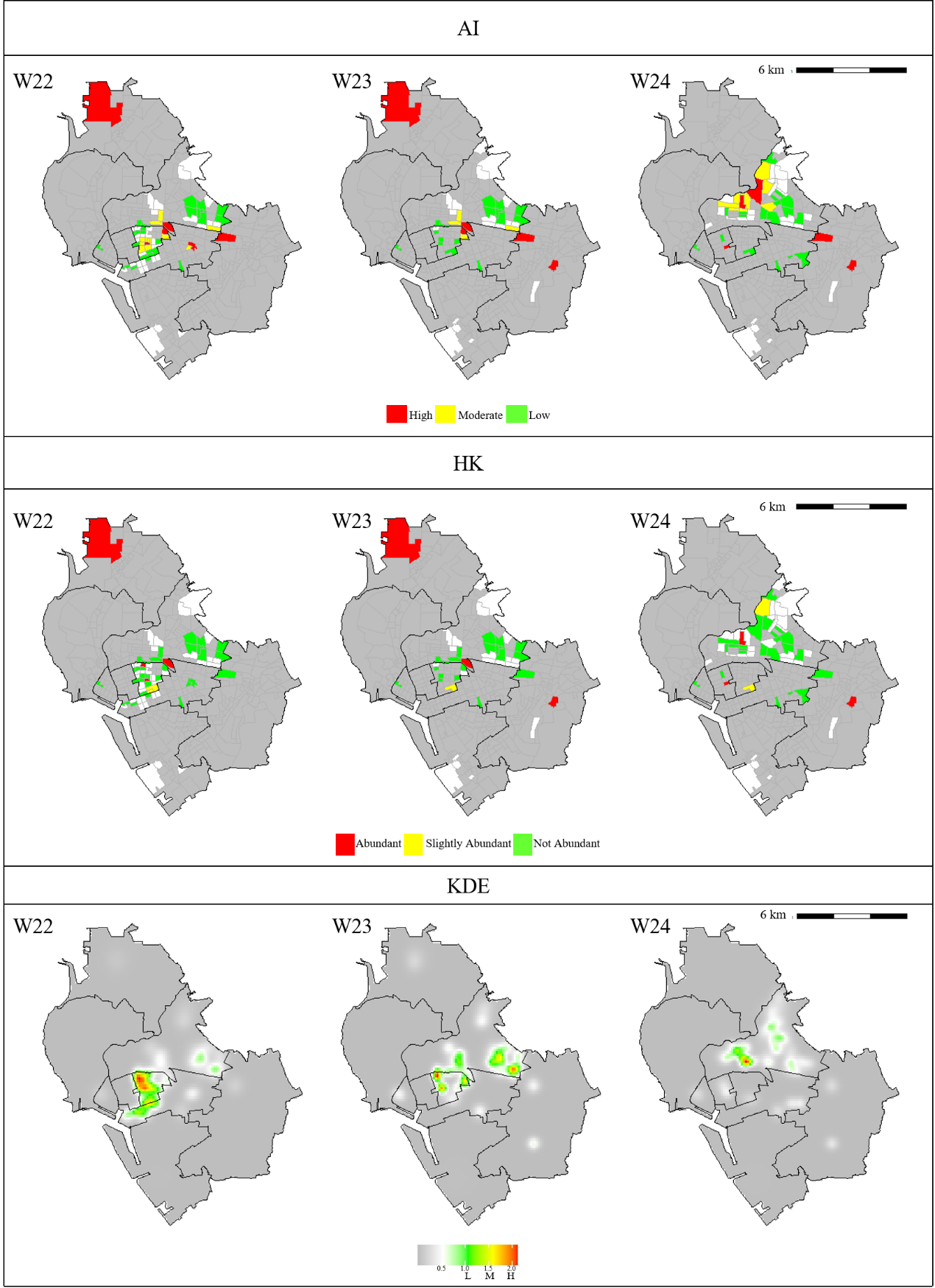


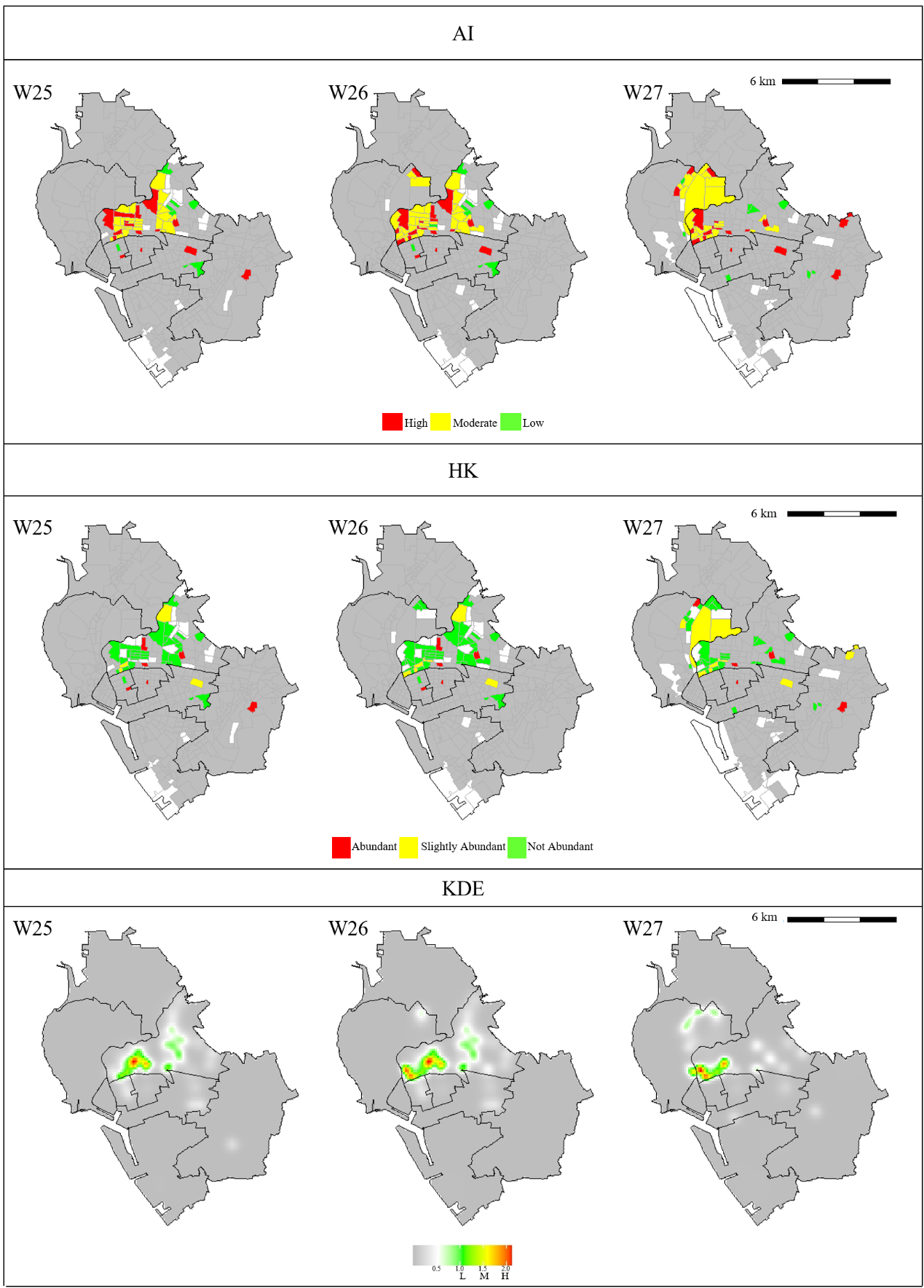


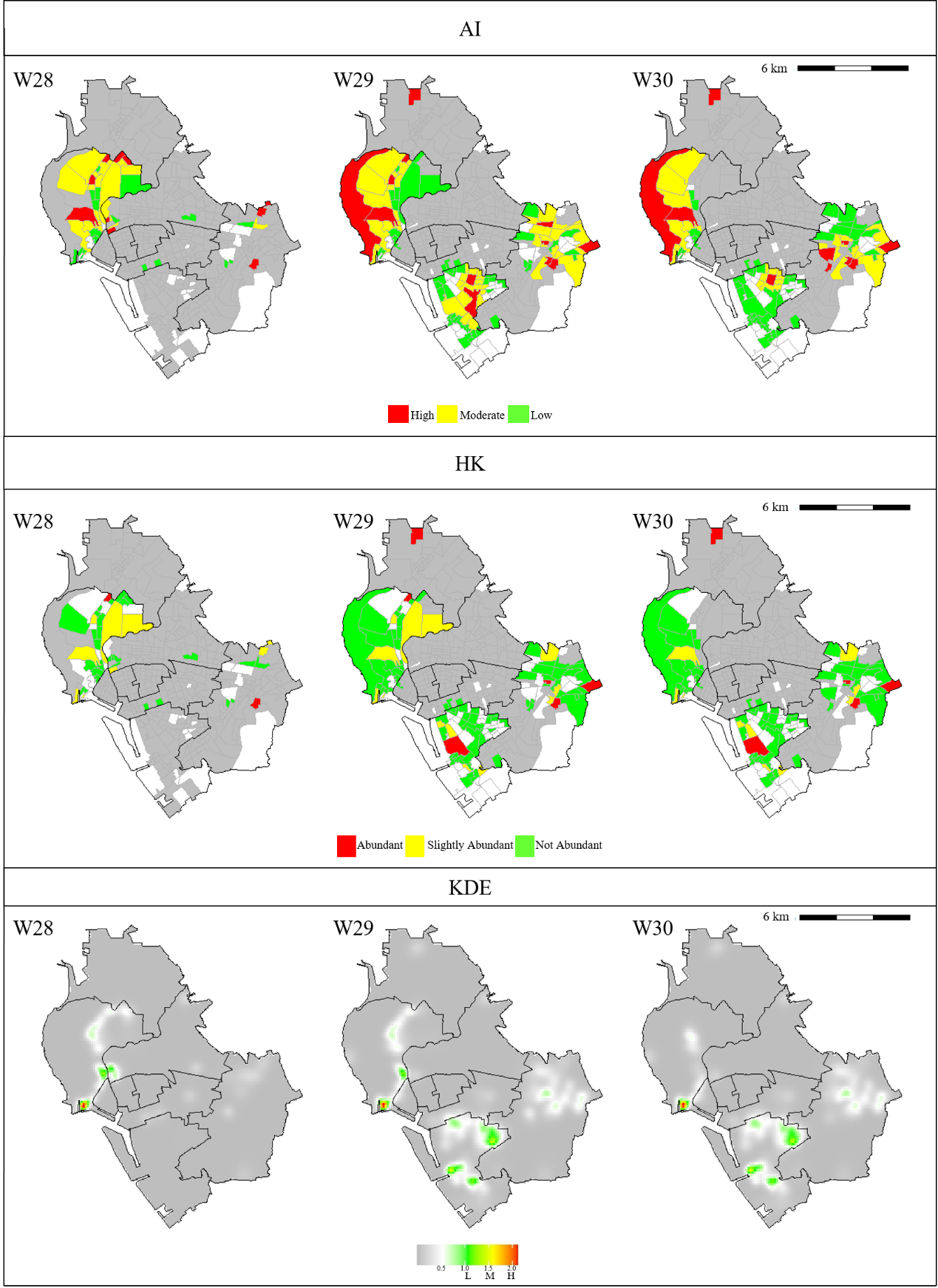


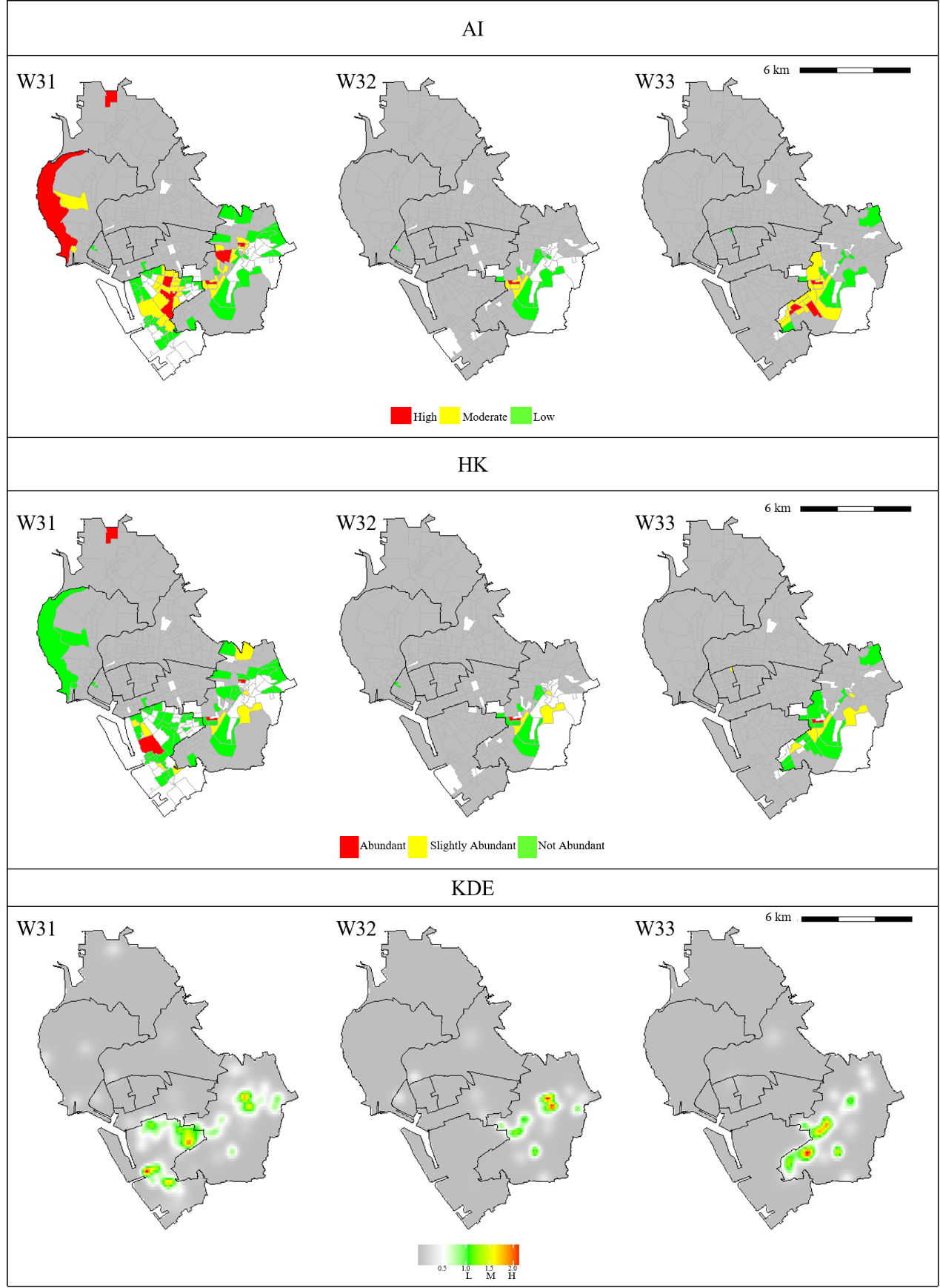


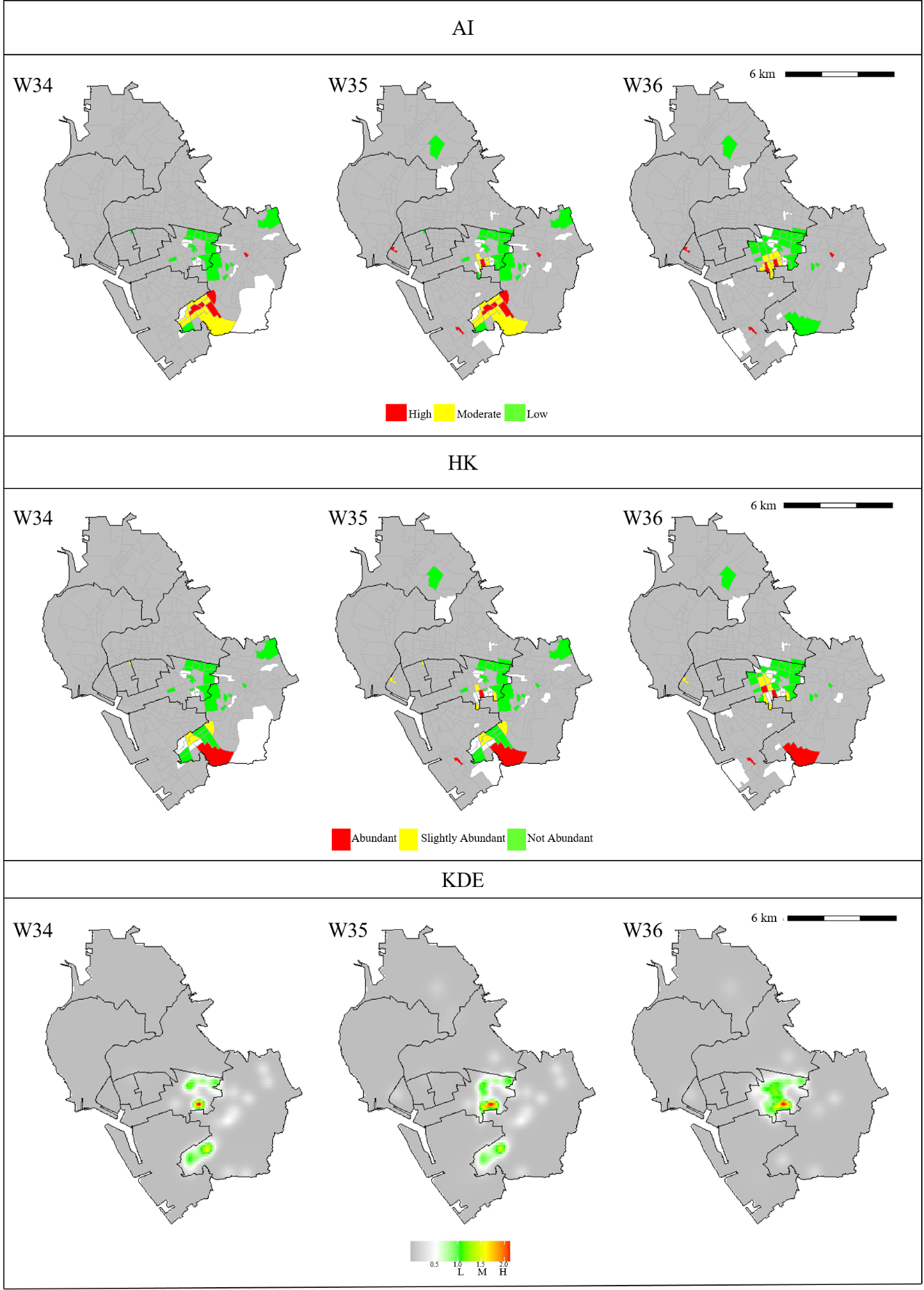


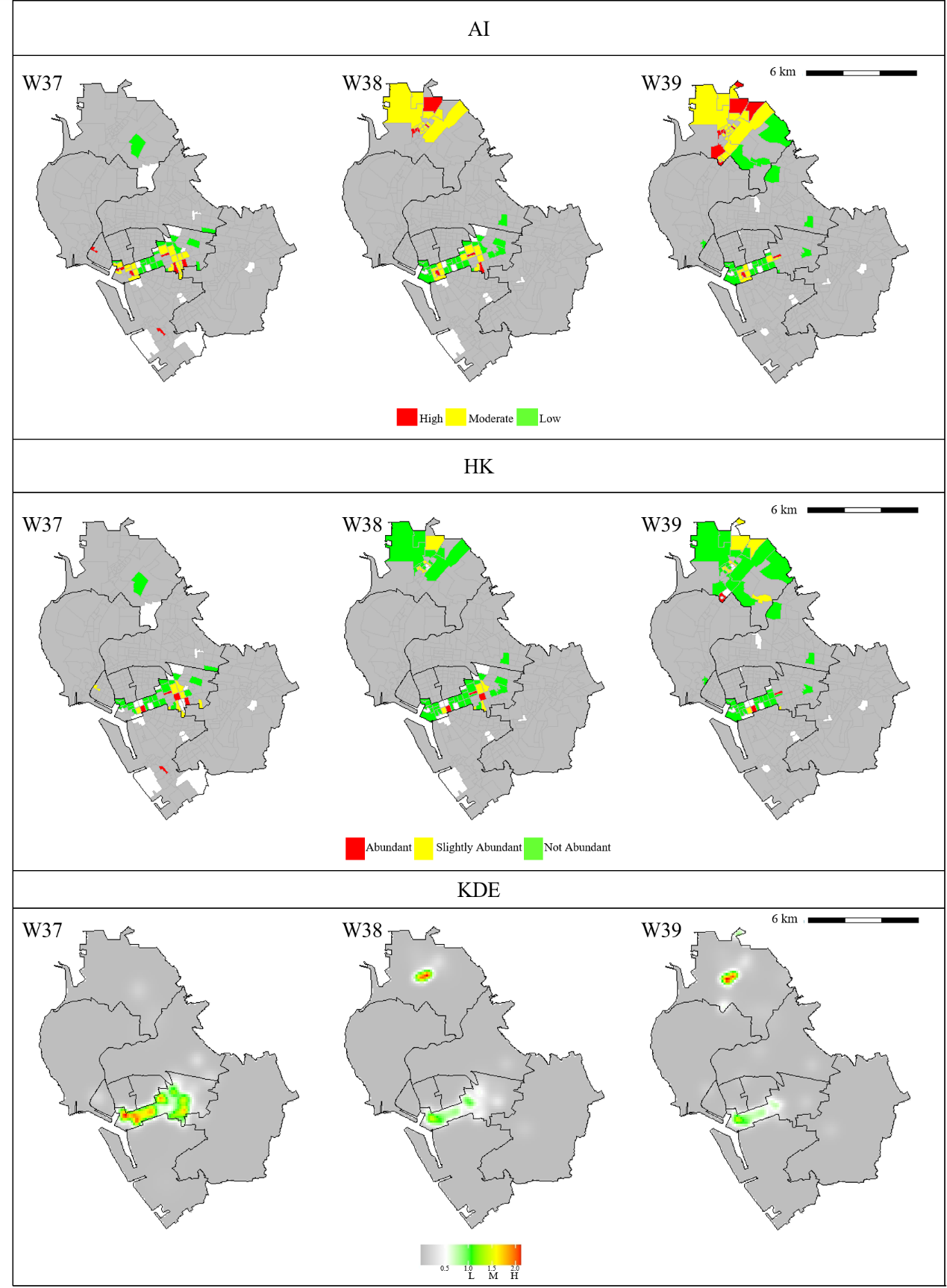


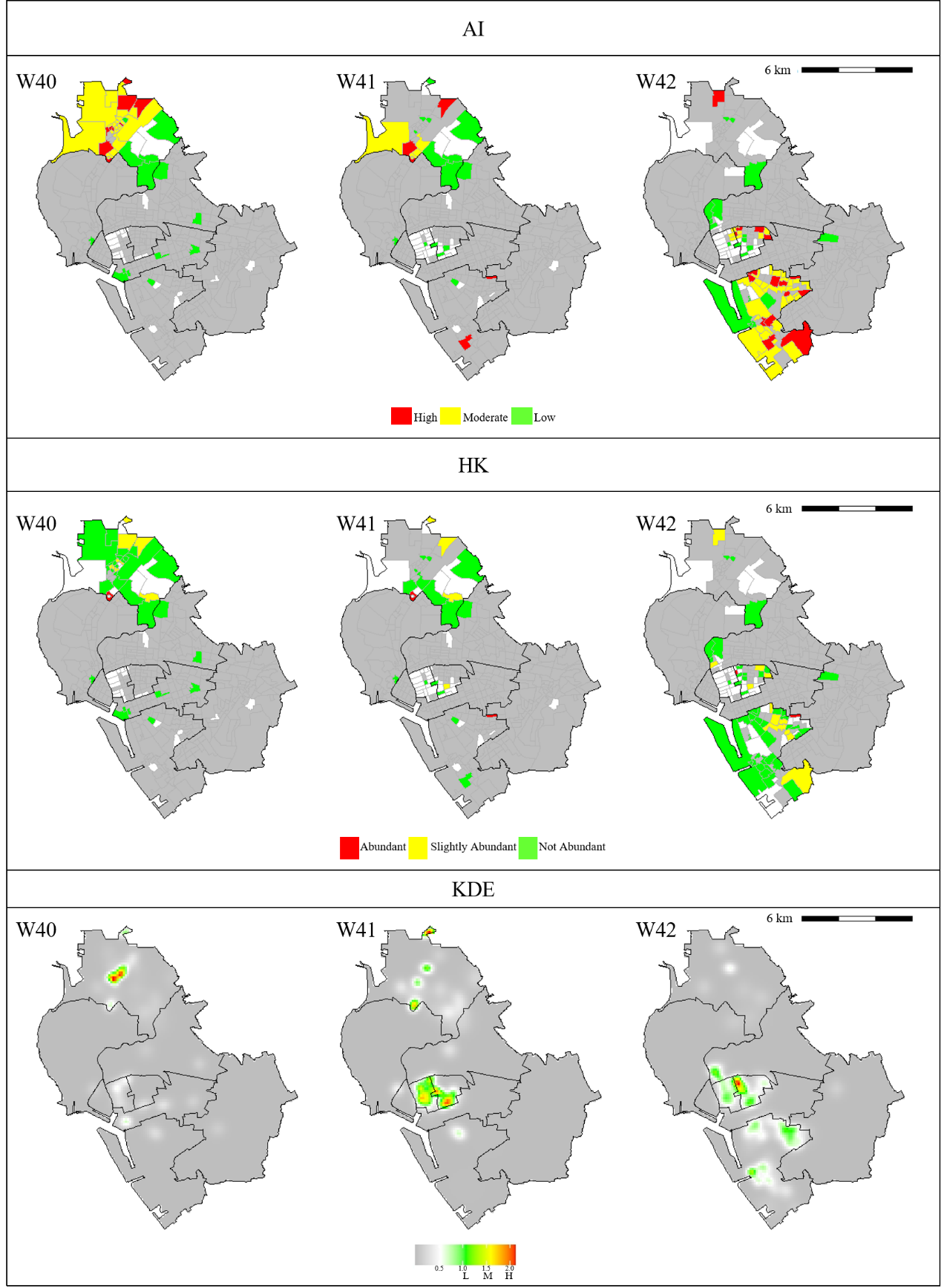

Supplement: Supplementary file 1 — Supplementary material 1. [file 12942_2025_403_MOESM1_ESM.docx]
